# International Multidisciplinary Consensus Report on Definitions, Diagnostic Criteria, and Management of Fatty Pancreas: A Joint Statement Endorsed by EPC, APA, EASD, EASL, ESGAR, ESGE, ESP, ESPCG, ESPEN, ESPGHAN, IAP, JPS, KPBA, LAPSG, and UEG

**DOI:** 10.1002/ueg2.70185

**Published:** 2026-02-14

**Authors:** Miroslav Vujasinovic, Ihsan Ekin Demir, Giovanni Marchegiani, Peter Hegyi, Livia Archibugi, Roberto Valente, Gabriele Capurso, Heiko Witt, Stefanos Bonovas, Daniele Piovani, Jonas Rosendahl, Patrick Maisonneuve, Caroline S. Verbeke, Muşturay Karçaaltıncaba, J. Enrique Dominguez‐Muñoz, Isabelle Scheers, Laszlo Czako, Robert Wagner, Vinciane Rebours, Daniel Öhlund, Ilkay S. Idilman, Kasper Overbeek, Natalya Gubergrits, Trond Engjom, Albrecht Neesse, Minoti Apte, Mihailo Bezmarević, Rickmer Braren, Stefania Bunduc, Güralp Onur Ceyhan, Manil Dinesh Chouhan, Anne Couvelard, Jérôme Cros, Daniel de la Iglesia, Enrique de‐Madaria, Joost P. H. Drenth, Asbjørn Mohr Drewes, Arantza Fariña Sarasqueta, Pierluigi Fracasso, Sven Francque, Jens Brøndum Frøkjær, Julio Iglesias‐Garcia, Pramod Garg, Felicia Gerst, Antanas Gulbinas, Ibrahim Halil Gürcinar, Martin Heni, Jong Jin Hyun, Eduard Jonas, Mariia Kiriukova, Masayuki Kitano, Aleksander Krag, Johanna Laukkarinen, Mónika Lipp, Martin Lovecek, Marc Martignoni, Etna Masip, Ryotaro Matsumoto, Anders Molven, Tetiana Mozhyna, Lenka Nosakova, Verena Obmann, Johann Ockenga, Sanjay Pandanaboyana, Nikola Panić, Georgios Papachristou, Analia Verónica Pasqua, Katarzyna M. Pawlak, Mario Pelaez‐Luna, Ivonne Regel, Sara Regnér, Stuart Robinson, Andrada Seicean, Vijay Singh, Mark M. Smits, Min Je Sung, Matteo Tacelli, Roy Taylor, Brigitta Teutsch, Mihaela Udrescu, Michael Wilschanski, Aslihan Yavas, Giulia A. Zamboni, J. Matthias Löhr

**Affiliations:** ^1^ Department of Upper Abdominal Diseases Karolinska University Hospital Stockholm Sweden; ^2^ Department of Medicine Huddinge Karolinska Institutet Stockholm Sweden; ^3^ Department of Surgery TUM University Hospital Klinikum Rechts der Isar Munich Germany; ^4^ Department of General Surgery HPB‐Unit School of Medicine Acibadem Mehmet Ali Aydinlar University Istanbul Turkey; ^5^ Department of Surgery Oncology and Gastroenterology (DiSCOG) Hepato Biliary Pancreatic (HPB) and Liver Transplant Surgery Padova University Padova Italy; ^6^ Centre for Translational Medicine Semmelweis University Budapest Hungary; ^7^ Institute for Translational Medicine Medical School University of Pécs Pécs Hungary; ^8^ Institute of Pancreatic Diseases Semmelweis University Budapest Hungary; ^9^ Translational Pancreatology Research Group Interdisciplinary Centre of Excellence for Research Development and Innovation University of Szeged Szeged Hungary; ^10^ Pancreato‐Biliary Endoscopy and Endosonography Division Pancreas Translational and Clinical Research Center IRCCS San Raffaele Scientific Institute Milan Italy; ^11^ Department of Diagnostics and Intervention Surgery Umeå University Umeå Sweden; ^12^ Division of Surgical Oncology Department of Surgery University of Colorado Aurora Colorado USA; ^13^ “Vita‐Salute” San Raffaele University Milan Italy; ^14^ Paediatric Nutritional Medicine Else Kröner‐Fresenius‐Centre for Nutritional Medicine Technical University of Munich (TUM) Freising Germany; ^15^ Department of Biomedical Sciences Humanitas University Milan Italy; ^16^ IRCCS Humanitas Research Hospital Milan Italy; ^17^ University Clinic Halle Clinic for Internal Medicine I Halle Germany; ^18^ Division of Epidemiology and Biostatistics IEO European Institute of Oncology IRCCS Milan Italy; ^19^ Department of Pathology Institute of Clinical Medicine University of Oslo and Oslo University Hospital Oslo Norway; ^20^ Department of Radiology Hacettepe University School of Medicine Ankara Turkey; ^21^ Department of Gastroenterology and Hepatology Health Research Institute (IDIS) University Hospital of Santiago de Compostela Santiago de Compostela Spain; ^22^ Department of Pediatrics Pediatric Gastroenterology and Hepatology Cliniques Universitaires Saint‐Luc Université Catholique de Louvain Brussels Belgium; ^23^ Department of Medicine Center of Gastroenterology University of Szeged Szeged Hungary; ^24^ Department of Endocrinology and Diabetology Medical Faculty and University Hospital Düsseldorf Heinrich Heine University Düsseldorf Düsseldorf Germany; ^25^ Institute for Clinical Diabetology German Diabetes Center Leibniz Center for Diabetes Research at Heinrich Heine University Düsseldorf Düsseldorf Germany; ^26^ German Center for Diabetes Research (DZD e.V.) Neuherberg Oberschleißheim Germany; ^27^ Pancreatology and Digestive Oncology Department Beaujon Hospital Université Paris‐Cité Clichy France; ^28^ Department of Diagnostics and Intervention Oncology Umeå University Umea Sweden; ^29^ Wallenberg Centre for Molecular Medicine (WCMM) Umeå University Umeå Sweden; ^30^ Department of Gastroenterology and Hepatology Erasmus MC University Medical Center Rotterdam the Netherlands; ^31^ Multidisciplinary Clinic Into Sana Odessa Ukraine; ^32^ Department of Medicine Haukeland University Hospital Bergen Norway; ^33^ Gastroenterology Gastrointestinal Oncology and Endocrinology University Medical Center Göttingen Göttingen Germany; ^34^ Faculty of Medicine and Health Pancreatic Research Group South Western Sydney Clinical Campuses School of Clinical Medicine University of New South Wales Sydney Australia; ^35^ Ingham Institute for Applied Medical Research Liverpool Hospital Liverpool Australia; ^36^ Department of Hepatobiliary and Pancreatic Surgery Clinic for General Surgery Military Medical Academy University of Defense Belgrade Serbia; ^37^ Institute for Diagnostic and Interventional Radiology TUM School of Medicine and Health TUM Klinikum Technical University of Munich (TUM) Munich Germany; ^38^ Department of Diagnostic and Interventional Radiology and Nuclear Medicine University Medical Center Hamburg‐Eppendorf Hamburg Germany; ^39^ Digestive Disease and Liver Transplant Center Fundeni Clinical Institute Bucharest Romania; ^40^ Carol Davila University of Medicine and Pharmacy Bucharest Romania; ^41^ Department of Diagnostic Radiology Princess Alexandra Hospital Brisbane Australia; ^42^ School of Medicine University of Queensland Brisbane Australia; ^43^ Department of Pathology Université Paris Cité APHP Bichat Hospital INSERM Paris France; ^44^ Department of Pathology Université Paris Cité APHP Beaujon Hospital INSERM Clichy France; ^45^ Department of Gastroenterology Pancreas Unit University Hospital of Puerta de Hierro Madrid Spain; ^46^ Department of Gastroenterology Dr. Balmis General University Hospital‐ISABIAL Alicante Spain; ^47^ Centro de Investigación Biomédica en Red de Enfermedades Hepáticas y Digestivas (CIBEREHD) Instituto de Salud Carlos III Madrid Spain; ^48^ Department of Gastroenterology and Hepatology Amsterdam UMC Amsterdam the Netherlands; ^49^ Department of Gastroenterology and Hepatology Center for Pancreatic Diseases and Mech‐Sense Aalborg University Hospital Aalborg Denmark; ^50^ Amsterdam UMC Location University of Amsterdam Department of Pathology The Netherlands Cancer Center Amsterdam Amsterdam the Netherlands; ^51^ Centro Medico Santagostino Milan Italy; ^52^ Department of Gastroenterology and Hepatology Antwerp University Hospital Edegem Belgium; ^53^ Laboratory for Experimental Medicine and Paediatrics Translational Sciences in Inflammation and Immunology Faculty of Medicine and Health Sciences InflaMed Centre of Excellence University of Antwerp Wilrijk Belgium; ^54^ Department of Radiology Radiology Research Center Aalborg University Hospital Aalborg Denmark; ^55^ Department of Clinical Medicine Aalborg University Aalborg Denmark; ^56^ Department of Gastroenterology All India Institute of Medical Sciences New Delhi India; ^57^ Institute for Diabetes Research and Metabolic Diseases of the Helmholtz Center Munich at the Eberhard Karls University of Tübingen (IDM) Tübingen Germany; ^58^ German Center for Diabetes Research (DZD e.V.) Neuherberg Oberschleißheim Germany; ^59^ Internal Medicine IV Endocrinology Diabetology and Nephrology University Hospital Tübingen Tübingen Germany; ^60^ Faculty of Health Sciences Klaipėda University Klaipėda Lithuania; ^61^ Department of Surgery Klinikum Rechts der Isar Technical University of Munich School of Medicine Munich Germany; ^62^ Division of Endocrinology and Diabetology Department of Internal Medicine I Ulm University Hospital Ulm Germany; ^63^ Institute for Clinical Chemistry and Pathobiochemistry University Hospital Tübingen Tübingen Germany; ^64^ Department of Internal Medicine Korea University Ansan Hospital Ansan Korea; ^65^ Department of Surgery Surgical Gastroenterology/Hepatopancreatobiliary Unit University of Cape Town and Groote Schuur Hospital Cape Town South Africa; ^66^ Department of Pancreatic, Biliary, and Upper GI Diseases Moscow Clinical Research Center A.S. Loginov Moscow Russia; ^67^ Second Department of Internal Medicine Wakayama Medical University Wakayama Japan; ^68^ Department of Gastroenterology and Hepatology Centre for Liver Research Odense University Hospital Odense Denmark; ^69^ Institute of Clinical Research University of Southern Denmark Odense Denmark; ^70^ Department of Gastroenterology and Alimentary Tract Surgery Tampere University Hospital Tampere Finland; ^71^ Faculty of Medicine and Health Technology Tampere University Tampere Finland; ^72^ Department of Surgery University Hospital Olomouc Olomouc Czech Republic; ^73^ Gastroenterology and Hepatology Pediatric Unit Hospital Universitari I Politecnic La Fe Valencia Spain; ^74^ Division of Gastroenterology Tohoku University Graduate School of Medicine Sendai Japan; ^75^ Gade Laboratory for Pathology Department of Clinical Medicine University of Bergen Bergen Norway; ^76^ Section for Cancer Genomics Haukeland University Hospital Bergen Norway; ^77^ Dr Krakhmalova Center for the Healthy Heart Kharkiv Ukraine; ^78^ Internal Gastroenterology Clinic University Hospital Martin Jessenius Faculty of Medicine in Martin Comenius University Bratislava Bratislava Slovakia; ^79^ Department of Radiology Zuger Kantonsspital Baar Switzerland; ^80^ Department of Diagnostic Interventional and Pediatric Radiology Inselspital Bern University Hospital University of Bern Bern Switzerland; ^81^ Department of Gastroenterology Hepatology and Clinical Nutrition Klinikum Bremen Mitte Bremen Germany; ^82^ Freeman Hospital Newcastle Upon Tyne UK; ^83^ University Clinic Dr Dragisa Misovic‐Dedinje Belgrade Serbia; ^84^ Division of Gastroenterology, Hepatology, and Nutrition Ohio State University Wexner Medical Center Columbus Ohio USA; ^85^ Hospital Italiano de Buenos Aires and LAPSG Latin American Pancreatic Study Group Buenos Aires Argentina; ^86^ Centre for Therapeutic Endoscopy and Endoscopic Oncology and Division of Gastroenterology St Michael's Hospital Toronto Ontario Canada; ^87^ Department of Physiology—Pomeranian Medical University Szczecin Szczecin Poland; ^88^ Research Division School of Medicine Universidad Nacional Autonoma de México Gastroenterology Department Instituto Nacional de Ciencias Médicas y Nutrición “Salvador Zubiran” Latin American Pancreas Study Group Mexico Mexico; ^89^ Department of Medicine II University Hospital LMU Munich München Germany; ^90^ Institute of Tumor Immunology Martin‐Luther‐University Halle‐Wittenberg Halle (Saale) Germany; ^91^ Department of Clinical Sciences Malmö Section for Surgery Lund University Malmö Sweden; ^92^ Department of Surgery Skåne University Hospital Malmö Sweden; ^93^ Newcastle Upon Tyne Hospitals NHS Foundation Trust Newcastle UK; ^94^ Regional Institute of Gastroenterology and Hepatology University of Medicine and Pharmacy “Iuliu Hatieganu” Cluj‐Napoca Romania; ^95^ Department of Medicine Mayo Clinic Arizona Phoenix Arizona USA; ^96^ Department of Internal Medicine Radboud University Medical Center Nijmegen the Netherlands; ^97^ Department of Gastroenterology CHA Bundang Medical Center CHA University Seongnam‐si Korea; ^98^ Magnetic Resonance Centre Translational and Clinical Research Institute Newcastle University Tyne UK; ^99^ Department of Radiology Medical Imaging Centre Semmelweis University Budapest Hungary; ^100^ Individual Family Practice Bucharest Romania; ^101^ Pediatric Gastroenterology Hadassah Hebrew University Medical Center Jerusalem Israel; ^102^ Institute of Pathology University Hospital Duesseldorf Heinrich Heine University Duesseldorf Germany; ^103^ Department of Diagnostics and Public Health Section of Radiology University of Verona Verona Italy; ^104^ Department of Clinical Science Intervention and Technology Karolinska Institutet Stockholm Sweden

**Keywords:** acute pancreatitis, beta‐cell, chronic pancreatitis, diabetes mellitus, fatty, intraductal papillary mucinous neoplasms, metabolic syndrome, pancreas, pancreatic cancer, pancreatic exocrine insufficiency

## Abstract

This international, multidisciplinary consensus report represents the first effort to systematically define and characterize fatty pancreas. A key outcome of this endeavor was the recommendation to adopt “fatty pancreas” as the standardized and inclusive term to describe all forms of fat accumulation in the pancreas. This terminological consensus provides a critical foundation for unified reporting and clinical communication. Another major contribution of the report is the consensus on diagnostic imaging findings, which was based on radiological and endoscopic modalities. The proposed criteria aim to enhance consistency in clinical assessment and support the development of standardized research protocols. In addition to establishing terminology and diagnostic frameworks, the report also synthesizes current knowledge across a wide range of relevant domains. These include the etiology and epidemiology of fatty pancreas, as well as its associations with alcohol consumption, smoking, acute and chronic pancreatitis, pancreatic exocrine insufficiency, type 2 diabetes mellitus, and surgical outcomes. The potential links between fatty pancreas and neoplastic conditions such as intraductal papillary mucinous neoplasms and pancreatic cancer are also addressed, alongside the current understanding of its metabolic implications (beta‐cell function and glucose homeostasis) and treatment strategies. Throughout the consensus process, a consistent theme emerged: the limited availability of high‐quality, prospective clinical data. Therefore, many of the recommendations in this report are based on expert consensus rather than strong empirical evidence. As such, the statements require rigorous prospective validation before they can be adopted into routine clinical practice. This underscores a critical need for further research, particularly studies aimed at clarifying causal relationships, validating diagnostic tools, and determining the clinical relevance of fatty pancreas across diverse patient populations. This report serves as both a summary of our current understanding and a roadmap for future investigations, aiming to close existing knowledge gaps and guide evidence‐based clinical practice in this emerging field.

## Key Summary

- Summarise the established knowledge on this subject?
  ◦ Fatty pancreas is associated with pancreatitis, metabolic disorders, and possible neoplastic changes.
  ◦ Multiple, inconsistent terms and definitions have historically been used, hindering unified clinical and research communication.
  ◦ Diagnosis relies on imaging, including radiological and endoscopic techniques, but lacks standardized criteria.
  ◦ The clinical impact and causal mechanisms of fatty pancreas remain uncertain due to limited prospective evidence.
- What are the significant and/or new findings of this study?
  ◦ Establishes “fatty pancreas” as the standardized, inclusive term for all forms of pancreatic fat accumulation.
  ◦ Provides consensus‐based diagnostic imaging findings utilizing radiological and endoscopic modalities.
  ◦ Integrates current evidence on etiology, epidemiology, associated conditions, metabolic effects, and treatment considerations for fatty pancreas.
  ◦ Identifies major research gaps and outlines priorities for future studies to clarify its clinical relevance and guide evidence‐based practice.

## 1 Introduction

A fatty pancreas is characterized by the abnormal accumulation of fat within the pancreatic tissue [1, 2]. The concept of fat accumulation in the pancreas has been recognized since the early 20th century [3], although it was originally considered a largely pathological finding, mainly observed at autopsy. Advances in imaging techniques have allowed for the more widespread recognition of fatty pancreas, particularly in individuals with metabolic risk factors [1]. Historically, fatty pancreas was seen mainly as a pathological curiosity, but it has gained greater attention in recent years, especially due to its associations with metabolic diseases [1] and possible links to pancreatitis [4], and even pancreatic cancer [5]. Despite this growing awareness, the pathophysiology and long‐term consequences of fatty pancreas remain subjects of ongoing research. The nomenclature surrounding fatty pancreas, however, remains inconsistent, posing a significant challenge to both research and clinical practice [1, 2]. These challenges and gaps in understanding, particularly regarding nomenclature, diagnosis, associations with other diseases, management, and follow‐up, were the driving forces behind the development of this consensus report.

## 2 Methods

The development of this consensus report adhered to the UEG framework for the development of high‐quality clinical guidelines, as proposed by the UEG Quality of Care Taskforce [6].

### 2.1 Initiative and the Steering Committee

The initiative for the consensus report came from the European Pancreatic Club (EPC) at the 56^th^ EPC Annual Meeting in Santiago de Compostela in June 2024. The Steering Committee was composed of two consensus report co‐chairs from the EPC (MV, IED), one senior member of the EPC Quality of Care and Guidelines Committee (PH), one council member of the EPC Quality of Care and Guidelines Committee (RV), the general secretary of the EPC (GC), the treasurer of the EPC (HW) and three EPC representatives of United European Gastroenterology (UEG)—the President (JML), Quality of Care Committee Chair (GM), and a Quality of Care Committee Member (LA). Members of the Steering Committee were selected based on their expertise in the field, ability to contribute to the work process, and their personal experience as effective team members familiar with the methodology of several previous guidelines [7, 8, 9, 10].

### 2.2 Participating Societies

The EPC invited other specialist member societies to join and endorse this project, with the aim of developing transversal international multidisciplinary consensus report to be adopted by all specialties. The following societies delegated participants and endorsed the project: EPC, American Pancreatic Association (APA), European Association for the Study of Diabetes (EASD), European Association for the Study of the Liver (EASL), European Society of Gastrointestinal and Abdominal Radiology (ESGAR), European Society of Gastrointestinal Endoscopy (ESGE), European Society of Pathology (ESP), European Society for Primary Care Gastroenterology (ESPCG), European Society for Clinical Nutrition and Metabolism (ESPEN), European Society for Pediatric Gastroenterology Hepatology and Nutrition (ESPGHAN), International Association of Pancreatology (IAP), Japan Pancreas Society (JPS), Korean Pancreatobiliary Association (KPBA), Latin American Pancreas Study group (LAPSG), and UEG (Table S1). Several patient associations also endorsed this project: Digestive Cancers Europe, Pancreatic Cancer Europe, Arbeitskreis der Pankreatektomierten e.V. (Germany), and PALEMA (Sweden).

### 2.3 Participants

All participants (*n* = 84) signed the Conflict of Interest Form (Supporting Information S1).

### 2.4 Statements, Grades of Evidence, and Outcome Reporting

The first meeting of the group was held online in January 2025. The working groups (Table S2) were finalized, and a leader responsible for each group (*n* = 16) was appointed. After the first meeting, each group performed a systematic review of the literature by searching PubMed, Web of Science, Cochrane, and Google Scholar (Supporting Information S2). The Steering Committee consulted experts in methodology and research synthesis, who were actively involved in all stages of the process. The statement format included the question, statement, Oxford level of evidence, the proportion of the global consensus group who agreed, and the comment in the final version. Statements were formulated in the context of the population/problem, intervention, comparison, and outcome (PICO) [11], where applicable. The PICO framework defines the population investigated, intervention in question, comparator for assessing an alternative option, and outcome(s) used to assess the intervention [6, 11]. The quality of evidence was appraised according to the Oxford Center for Evidence‐Based Medicine system, where the evidence was graded from 1 to 5, with 1 being the highest and 5 being the lowest [6, 12]. The Oxford Center for Evidence‐Based Medicine system was selected to grade the evidence and support the consensus statements, as it accommodates both empirical data and expert judgment in areas where high‐quality evidence is limited [12]. In cases where insufficient evidence was available to support a definitive position, the Oxford level of evidence was designated as “not applicable” (N/A). In such instances, consensus was based on the panel's collective judgment and the current state of knowledge, acknowledging gaps in the evidence base.

### 2.5 Delphi Process

Questions, statements, and related comments were uploaded to the Delphi online platform at the end of April 2025 and voted on in May 2025. All participants were given the opportunity to vote on and comment on every statement included in the report. Each statement was evaluated using a modified 5‐point Likert scale [13, 14], with the following response options: “strongly disagree,” “disagree,” “agree,” “strongly agree,” and “not qualified to respond.” The inclusion of the “not qualified to respond” option was intended to reflect the varied backgrounds of the participants and to ensure that responses were based on areas of individual expertise (such responses were excluded from the final consensus calculations). Participants were encouraged to provide written comments to support their responses or suggest refinements to the statements. All votes and accompanying feedback were reviewed by the coordinating group. A level of agreement, defined as the combined proportion of “agree” and “strongly agree” responses of 80% or higher, was considered to indicate consensus (consensus threshold was defined a priori). Although two iterative Delphi rounds were originally planned, with discussion and revision of statements between rounds, only a single voting round was conducted, as all statements reached consensus in the first round. The statements were discussed at the 57th EPC Annual Meeting in Düsseldorf, Germany, in July 2025, to ensure agreement and make minor adjustments (which were mostly related to language editing/phrasing). Following the consensus reached after the EPC 2025 and a final round of adjustments, the manuscript was finalized.

#### 2.5.1 Chapter 1

*Question:* What is the appropriate term?

*Statement 1: The most suitable and encompassing term to describe all forms of fat in the pancreas is “fatty pancreas,” and we recommend its adoption for general use.*

*Level of evidence:* N/A ‐ this is a consensus‐based terminology decision, not a clinical question evaluated through evidence.

*Consensus agreement:* 87%

*Comment:* Since it was first described, more than a century ago, several synonyms for fat in the pancreas have been used in the literature [1, 2], including pancreatic lipomatosis [15], pancreatic steatosis [16], intrapancreatic fatty infiltration [17], fatty pancreas [2, 18], lipomatous pseudohypertrophy of the pancreas [19], non‐alcoholic fatty pancreas disease [20], intrapancreatic fat deposition [21], ectopic fat in the pancreas [22], fat deposition in the pancreas [23], fat replacement of the pancreas [24], adipose atrophy of the pancreas [25], pancreatic fat accumulation [26], and non‐alcoholic fatty steatopancreatitis [2]. After thorough discussion and consideration, we propose “fatty pancreas” as the most appropriate and universally acceptable term to describe the condition of fat within the pancreas. While the term “non‐alcoholic fatty pancreas disease” has gained some popularity, particularly due to its similarity to “non‐alcoholic fatty liver disease” within the context of metabolic syndrome, it is crucial to emphasize the significant differences between both organs and their respective pathological processes. These distinctions make it inappropriate to directly compare both diseases or to draw nomenclature inspiration from fatty liver disease. Another argument against the use of “non‐alcoholic fatty pancreas disease” is that it creates a nomenclature based on negations, which is generally undesirable in medical terminology. The term “non‐alcoholic” is a negative construction, which adds unnecessary complexity and does not accurately describe the nature of this specific pancreatic condition, which is distinct and unrelated to alcohol‐related damage. Furthermore, while there is a clear justification for the term “non‐alcoholic fatty liver disease” (recently changed to “metabolic dysfunction‐associated steatotic liver disease” [27]) ‐ due to the near‐identical histology observed in both alcoholic and non‐alcoholic liver steatosis; this similarity does not extend to the pancreas. The etiology and histopathology of fatty pancreas are described in questions and statements 2 and 5 of this consensus report, and we refer readers to those sections for a more comprehensive understanding of this specific topic.

#### 2.5.2 Chapter 2

*Question:* What is the etiology of fatty pancreas?

*Statement 2: Various factors, including aging, obesity, inflammation from diverse causes, genetic disorders, and chronic obstruction, contribute to a fatty pancreas. Multiple intra‐ and extra‐pancreatic cell types seem to have the potential to serve as precursors of intrapancreatic adipocytes.*

*Level of evidence:* 4.

*Consensus agreement:* 89%

*Comment:* The etiology of fatty pancreas is multifactorial, involving processes such as aging, inflammation, fibrosis, and as a consequence of neoplasia [28]. Obesity is also a significant contributor to this condition, as excessive systemic lipid levels lead to an increase in both the number and size of adipocytes, which can infiltrate various organs, including the pancreas, potentially replacing normal pancreatic tissue [2, 15, 29]. A correlation with type 2 diabetes mellitus (DM) has been reported [30]. In patients with type 2 DM, liver fat export via very low‐density lipoprotein 1 (VLDL1)‐triglycerides influences pancreatic fat accumulation, a process that seems to be reversible upon diabetes remission [31]. Additionally, obstruction of the pancreatic duct (due to conditions such as malignancy or chronic pancreatitis) can lead to atrophy and fatty pancreas [32]. Cystic fibrosis also frequently leads to the fatty pancreas, a change that becomes more pronounced in older patients [33, 34]. In some rare inherited disorders, such as those involving mutations in carboxyl ester lipase (CEL), or syndromes like Johanson–Blizzard or Shwachman–Diamond, fat can accumulate in the pancreas without inflammatory processes, partly preceding clinical signs of impaired function [35, 36].

While lipid droplet accumulation has been observed in pancreatic exocrine and endocrine cells, most of these findings are primarily derived from animal models [37, 38, 39]. Physiologically, both adipocytes and lipid droplets play a role in maintaining cellular homeostasis by serving as energy reservoirs, and, for lipid droplets, by regulating intracellular lipid levels. However, disruptions in this balance may induce cytotoxic effects or promote inflammation [40]. Nonetheless, the precise cellular origin of intrapancreatic adipocytes remains unclear. Hypotheses for this include the transdifferentiation of exocrine pancreatic cells, differentiation of tissue‐resident stem cells, and infiltration by adipocyte precursors from external sources, such as peripancreatic fat [28, 36].

Recent animal studies utilizing high‐fat diet models, experimental pancreatitis, and various genetic manipulations targeting acinar and ductal cells involved in pancreatic morphogenesis have revealed different mechanisms of fatty pancreas alteration [28]. These findings suggest that multiple cell types may serve as precursors to intrapancreatic adipocytes. Generally, two distinct scenarios may underlie the development of a fatty pancreas: one that parallels measures of adiposity [41, 42], and the other resulting from exocrine pancreatic tissue loss [43]. A proposal for the classification of fatty pancreas by etiology is presented in Table 1.

**TABLE 1:** Proposal for the classification of fatty pancreas by etiology.

Metabolic causes	Anthropometric/anatomic causes	Common exocrine pancreatic diseases^a^	Rare genetic disorders
Metabolic syndrome (all components) Metabolic dysfunction‐associated steatotic liver disease (MASLD) Adiposity	Aging Sex hormones Extremely low birthweight Pancreatic duct obstruction	Chronic pancreatitis Pancreatic adenocarcinoma Other pancreatic neoplasms	Cystic fibrosis Shwachman–Diamond syndrome Johanson–Blizzard syndrome Pearson syndrome Maturity‐onset diabetes of the young, type 8 (CEL‐MODY) β‐thalassemia Diamond–Blackfan anemia Hereditary hemochromatosis Hereditary chronic pancreatitis

*Note:* Adapted from: [1, 2, 249, 250, 251].

^a^ fatty pancreas most likely results from acinar atrophy secondary to pancreatic duct obstruction.

#### 2.5.3 Chapter 3

*Question:* What is the role of alcohol consumption and smoking in the development and progression of fatty pancreas?

*Statement 3: Currently, there is no convincing evidence of an increased prevalence or a causal relationship between alcohol consumption and cigarette smoking in the etiopathogenesis of fatty pancreas.*

*Level of evidence:* 3.

*Consensus agreement:* 97%

*Comment:* The role of alcohol consumption in the development of fatty pancreas has been suggested by preclinical studies in rat models [44, 45]. Beyond animal models, only a limited number of heterogeneous human studies have examined the association between alcohol consumption and fatty pancreas development, with most exploring this relationship (alongside factors like cigarette smoking) as part of broader investigations rather than as a primary focus. While most current evidence does not support a causal relationship between alcohol consumption or smoking and the development or progression of fatty pancreas, a few observational studies have suggested potential associations. However, these findings are often based on heterogeneous or cross‐sectional data, and prospective, well‐powered studies are lacking. A cross‐sectional magnetic resonance imaging (MRI) study of 119 individuals after acute pancreatitis found that fatty pancreas was more common in these patients than in healthy controls, regardless of alcohol or smoking [46]. Similarly, in a cross‐sectional study of 8097 individuals, including 1297 with fatty pancreas and 6800 without, researchers found no significant differences in the prevalence of smoking or alcohol consumption between the two groups [47]. Moreover, a five‐year follow‐up study of 320 Japanese adults without metabolic syndrome found that higher pancreatic fat levels were linked to a greater proportion of smokers and individuals consuming at least 20 g of alcohol per day [48]. A study of 685 healthy volunteers without metabolic syndrome used MRI to diagnose fatty pancreas, applying a fat content threshold of 10.4%; it found no significant difference in smoking rates between those with and without fatty pancreas (8.2% vs. 12.7%). However, current alcohol consumption was slightly higher in individuals with fatty pancreas compared to those without (28.2% vs. 19.5%), showing borderline significance (*p* = 0.04) [49]. Furthermore, several studies have consistently found no significant association between alcohol consumption or smoking and the presence or progression of fatty pancreas. In a prospective study of 250 patients undergoing endoscopic ultrasound (EUS), fatty pancreas was detected in 27.8% of cases, but neither alcohol nor smoking was linked to increased risk [50]. Similarly, a large cohort study involving over 9900 individuals found no significant differences in smoking or alcohol use between those with and without fatty pancreas [51]. A retrospective study with a 2‐year follow‐up further supported these findings, showing no association between alcohol or smoking and pancreatic parenchymal changes in patients with fatty pancreas [52]. Although modification of lifestyle risk factors is a fundamental component in the management of patients with fatty pancreas, no studies to date have systematically evaluated the potential benefits of reducing or ceasing alcohol consumption and/or smoking as preventive strategies for the onset and progression of fatty pancreas.

#### 2.5.4 Chapter 4

*Question:* What is the epidemiology of fatty pancreas?

*Statement 4: The epidemiology of fatty pancreas is poorly understood. Due to the lack of standardized definitions and diagnostic criteria, prevalence estimates vary widely across studies. However, fatty pancreas is more common with increasing age, body mass index, waist circumference, metabolic syndrome, and diabetes.*

*Level of evidence:* 2.

*Consensus agreement:* 99%

*Comment:* A recent systematic review and meta‐analysis of the prevalence, clinical characteristics, and outcomes of fatty pancreas reported an overall prevalence of 21%, with significant differences across countries, geographical regions, and variations according to diagnostic modality, but no significant differences based on study settings or sample size. Moreover, sex, mean age, mean body mass index (BMI), and the percentage of patients with DM or fatty liver diseases had no effect on heterogeneity [18].

**FIGURE 1:**
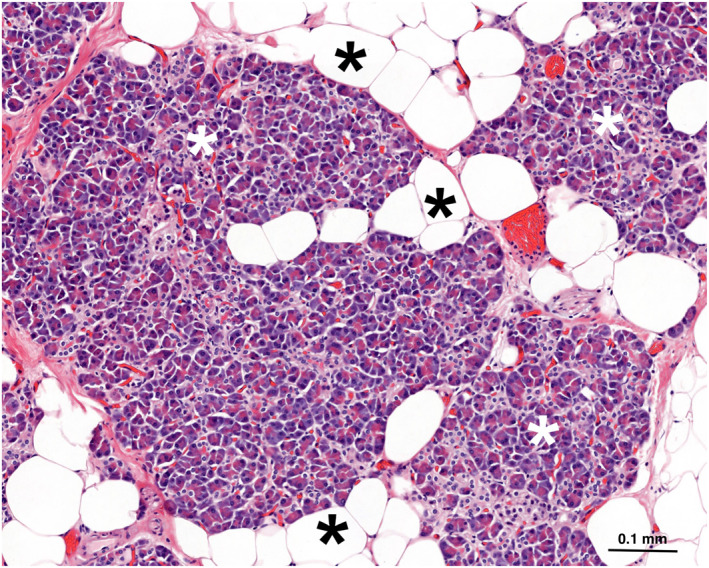
Mature adipocytes (*black asterisks*) around and within parenchymal lobules (*white asterisks*). Note the absence of lipid vacuoles in acinar cells.

**FIGURE 2:**
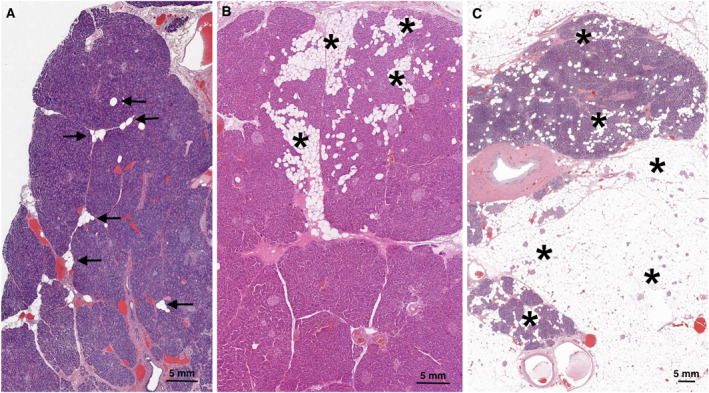
The degree of fatty pancreas‐related changes (*arrows, asterisks*) may range from discrete (a) to moderate (b) and severe (c).

**FIGURE 3:**
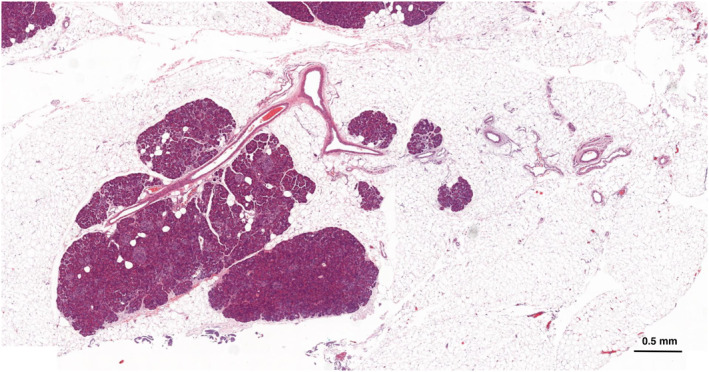
Non‐uniform distribution of fatty pancreas‐related changes resulting in mild alteration in some lobules (left) and advanced changes in flanking parenchyma (right).

An updated systematic review using multiple literature databases (performed for the purposes of the present consensus report) led to the identification of 26 studies reporting on the prevalence of fatty pancreas in the adult population or screened subjects free of pancreatic diseases, with a pooled prevalence of 27% (range, 1.2%–70.8%) [15, 25, 47, 49, 50, 51, 53, 54, 55, 56, 57, 58, 59, 60, 61, 62, 63, 64, 65, 66, 67, 68, 69, 70, 71, 72] (Supporting Information S3: Figures 1–3). Diagnosis of fatty pancreas was based on transabdominal ultrasound (US), with or without elastography, in 14 studies; MRI in four studies; EUS and computed tomography (CT) in three studies each; and autopsy specimens in two older studies. The variability across studies (in terms of population characteristics, age distribution, and differences in diagnostic methods or criteria) prevents a reliable estimation of overall prevalence based on specific demographic or clinical factors.

#### 2.5.5 Chapter 5

*Question:* What is the histopathology of fatty pancreas?

*Statement 5.1: The hallmark of fatty pancreas is the presence of adipocytes, intralobularly and/or extralobularly. Extensive adipocyte accumulation is usually associated with loss of acinar parenchyma.*

*Level of evidence:* 5.

*Consensus agreement:* 100%

*Statement 5.2: The distribution of fatty pancreas‐related changes may be patchy.*

*Level of evidence:* 5.

*Consensus agreement:* 100%

*Statement 5.3: The histology of fatty pancreas beyond the presence of adipocytes has been poorly studied.*

*Level of evidence:* N/A.

*Consensus agreement:* 100%

*Statement 5.4:* There is currently no universally accepted, validated objective method for the histological assessment of the severity of fatty pancreas.

*Level of evidence:* N/A.

*Consensus agreement:* 100%

*Comment:* The presence of non‐neoplastic adipocytes in the pancreas is the hallmark of fatty pancreas. In this context, the adipocytes are morphologically mature and contain a single, large lipid vacuole (Figure 1). They may be located within pancreatic lobules (intralobular) and/or interlobular spaces (extralobular) (Figure 2), and in exceptional instances, inside islets of Langerhans (Figure 3). In contrast to hepatic steatosis, intracytoplasmic lipid vacuoles in acinar cells are not readily seen upon hematoxylin and eosin staining. Histochemical, immunohistochemical, and ultrastructural examinations have revealed acinar intracytoplasmic lipid accumulation in rodent models of fatty pancreas [37, 73, 74, 75, 76]. While this phenomenon has been less documented in human pancreas [77], a recent study indicates that intracellular lipid accumulation in human pancreas may be seen in both acinar cells and endocrine cells of Langerhans islets [78]. The number of adipocytes may vary from a few scattered fat cells to confluent sheets (Figure S4). Extensive adipocyte accumulation is usually associated with loss of acinar parenchyma, ultimately resulting in vast stretches of adipose tissue containing scattered islets, ducts, or sparse clusters of residual acinar cells (Figure S5). Macroscopically, when changes are advanced, the pancreas may exhibit a marbled appearance and a markedly soft texture [79]. According to autopsy studies, the distribution of fatty pancreas‐related changes may be patchy [29, 42] (Figure S6), a finding supported by imaging studies [80]. More recent histological studies are primarily based on the examination of surgical specimens, which precludes the assessment of the distribution of fatty pancreas‐related changes. However, the frequent involvement of superficial lobules, that is, lobules at the interface with the peripancreatic tissues, has been described [81].

The histology of fatty pancreas beyond the presence of adipocytes has been poorly studied. While inflammatory changes and fibrosis are not usually conspicuous in and around the adipocytes and the neighboring parenchyma, these changes have not been systematically characterized. Currently, there is only sparse published evidence from a small number of human and animal studies [82, 83, 84]. Nonetheless, current published evidence does not suggest that increasing adipocyte accumulation is associated with inflammation and fibrosis. Hence, a sequence similar to that seen in the liver, that is, steatosis progressing to steatohepatitis, is not generally observed in the pancreas. Furthermore, data and insight are lacking regarding possible variations in histomorphological features with respect to the underlying cause of fatty pancreas. There is also currently no universally accepted, validated objective method for the histological assessment of the severity of fatty pancreas. Proposed grading systems vary from subjective manual semiquantification (based on visual inspection) [85], to the percentage of pancreatic tissue area occupied by adipocytes [86, 87, 88], or the number of adipocytes per microscopy field [89], to automated morphometric analysis of images or digital sections [90, 91, 92, 93]. Intra‐ and extralobular fat is assessed separately; however, distinction may be impossible in advanced fatty pancreas. As the amount of peripancreatic fat contained in a specimen may depend on the surgical procedure, it is excluded from the assessment. A line connecting the most peripheral (remnants of) acinar cells and/or islets defines the border between extralobular and peripancreatic fat (Figure S7). Because peripancreatic fat is typically absent at the pancreatic neck, tissue samples from this location are commonly analyzed [88, 92, 94]. Given the potential non‐uniform distribution of fatty pancreas‐related changes, various recommendations have been made regarding the number of tissue blocks to be examined; however, none of these have been validated. A systematic assessment of the degree of heterogeneity may provide valuable information about the minimum number of tissue blocks needed for the reproducibility of findings [95]. While the scoring of fatty pancreas‐related changes is currently not part of routine diagnostic procedures, evaluation of the presence of adipocytes at the transection margin may be considered, given its correlation with the risk for postoperative pancreatic fistula [96, 97].

As only a small number of studies have systematically analyzed the histology of human fatty pancreas, insight is currently limited. With surgical specimens accounting for most of the study material available, potential interference by pancreatic disease that necessitates resection represents a significant limitation, even if tissue samples are taken from upstream or at least 1 cm away from the tumor [98, 99]. Due to the dearth of evidence and, consequently, the lack of consensus regarding the histomorphological features and diagnostic criteria of fatty pancreas, currently available study findings are difficult to compare.

#### 2.5.6 Chapter 6

*Question:* How should fatty pancreas be diagnosed from a radiological standpoint?

The presence and severity of fatty pancreas can be evaluated using various imaging modalities in both clinical practice and research, including transabdominal US, CT, and MRI.

*Statement 6.1: Transabdominal ultrasonography could suggest the presence of fatty pancreas; however, its diagnostic reliability and utility for assessing severity remain limited due to operator dependency, technical variability, and inconsistent criteria.*

*Level of evidence:* 3.

*Consensus agreement:* 87%

**FIGURE 4:**
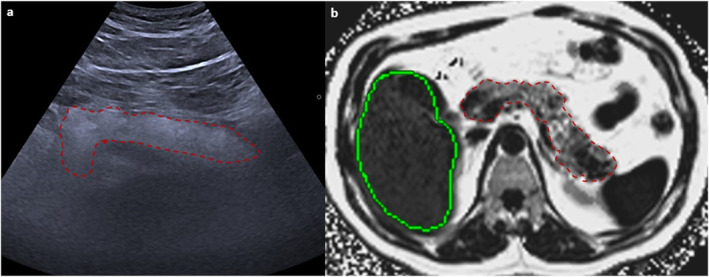
Transabdominal ultrasonography image (a) of a patient with fatty pancreas showing increased echogenicity, which was confirmed via magnetic resonance imaging‐proton density fat fraction (b).

*Comment:* Transabdominal US is one of the most common imaging modalities for evaluating abdominal organs. Increased echogenicity can indicate the presence of fat in the pancreas (Figure 4). However, echogenicity can be affected by other pathological processes, including fibrosis, inflammation, and calcification [100]. Moreover, the retroperitoneal location of the pancreas poses a limitation for its evaluation, especially in individuals with obesity; evaluating the whole organ can be difficult and may result in significant interobserver variation. Echogenicity depends on the body habitus of the patient, as well as the frequency of the transducer, B‐mode gain presets, and the angle of insonation. Comparisons of pancreatic echogenicity with other organs, such as the liver, spleen, kidney, and retroperitoneal fat, have been proposed; however, they lack validation and consistent application [23, 53, 101, 102]. Moreover, no established validated quantitative criteria exist; however, most semi‐quantitative methods have four grades (non, mild, moderate, or severe). Therefore, a cautious approach is advisable when comparing the data, given the possibility of methodological variations. A four‐tier grading system for fatty pancreas based on echogenicity relative to surrounding organs has been proposed [103]. However, if the reference organ (e.g., liver) is itself steatotic, this comparison becomes unreliable. Furthermore, this grading system was not compared to other imaging modalities; therefore, the sensitivity for severity grading and detecting mild fatty pancreas could be questionable.

*Statement 6.2: CT could assist in the diagnosis of fatty pancreas, particularly in advanced cases, but its sensitivity is limited for detecting milder forms.*

*Level of evidence:* 5.

*Consensus agreement:* 97%

**FIGURE 5:**
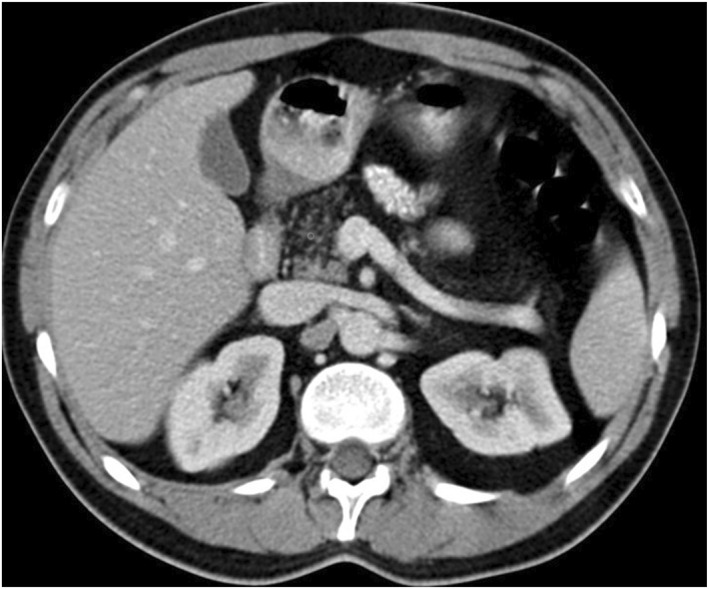
Diffuse fatty pancreas on computed tomography is more evident in the tail and the body than in the head.

*Comment:* CT is a cross‐sectional imaging modality for whole organ evaluation of the pancreas, showing decreased attenuation values in cases of fatty pancreas [104] (Figure 5). Measuring the Hounsfield unit (HU) of the tissue of interest has the potential to quantitatively grade the degree of fatty pancreas. However, attenuation values may differ between various CT scanners, depending on the scan parameters and contrast dosing, which can reduce the reproducibility of the technique. Moreover, a mild degree of fat can cause a hypodense appearance on CT (the so‐called “invisible fat” on CT), which is not enough to confidently diagnose the presence of fat [105]. Radiation exposure is another limitation of the technique. The difference in attenuation between the spleen (in the absence of splenic pathology) and pancreas, as well as the ratio of these values on non‐enhanced CT, has been shown to be accurate for evaluating fatty pancreas in comparison with histopathological analyses [90]. A pancreas‐to‐spleen density ratio of < 0.7 is proposed to be a diagnostic criterion for the presence of fatty pancreas [106]. Additionally, the combination of pancreatic surface lobularity and pancreatic attenuation on CT has demonstrated good accuracy for detecting fatty pancreas [107].

*Statement 6.3: MRI, particularly the use of proton density fat fraction (PDFF) sequences, is the most reliable technique for accurate pancreatic fat quantification.*

*Level of evidence:* 3.

*Consensus agreement:* 100%

*Comment:* MRI is the preferred choice of imaging for the quantitative assessment of pancreatic fat, even though it is costly and often has limited availability. The principle of MRI in detecting and quantifying fat mainly depends on the chemical shift effect, which can be defined as the difference in resonance frequencies between hydrogen protons bound to triglycerides and water [108]. Chemical shift imaging, Dixon MRI‐PDFF, and MR spectroscopy (MRS) are the best options for both detecting and quantifying fatty pancreas. In‐phase and opposed‐phase imaging exploits the time‐of‐echo‐dependent phase interference effect between gradient echo signals of water and fat. However, it has some disadvantages, as it only evaluates the main fat peak in the tissue, and is also subject to biases from T1 and T2* relaxation. MRS is another method that can also measure the fat fraction of the pancreas; however, it has some technical challenges, including limited voxel size, which can be a problem in heterogeneous fat infiltration.

**FIGURE 6:**
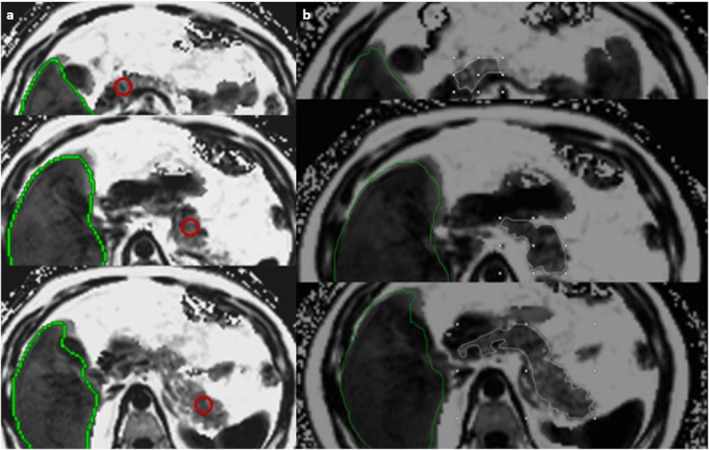
Fat fraction quantification with magnetic resonance imaging‐proton density fat fraction. It is both feasible to quantify pancreatic fat with three regions of interest (ROIs) placed on the pancreatic head, body, and tail to report the average (a) and to measure the entire pancreas using free‐hand ROIs (b).

Due to the technical limitations of the in‐phase and opposed‐phase methods and MRS, MRI‐PDFF is recognized as the most reliable method for quantifying pancreatic fat, as it enables the evaluation of the entire pancreas with shorter scan times, as opposed to a voxel‐limited technique like MRS. The histological pancreatic fat fraction is highly correlated with MRI‐PDFF [92, 109]. Commonly, measurement of the averages of three regions of interest (ROIs) of the head, body, and tail of the pancreas should be conducted with due care to avoid non‐parenchymal structures, such as ducts and vessels (Figure 6). Because of significant variations in organ volume [110, 111] and on the principle that smaller ROIs are more susceptible to noise, maximally sized ROIs are advisable for measurement. Pancreatic organ margins can become physiologically and pathologically indistinct over time due to senile and pathological changes. Different MRI studies evaluating the fat distribution between pancreatic regions have demonstrated conflicting results. The most comprehensive study revealed an unequal distribution of pancreatic fat in 1367 volunteers, with pancreatic fat fractions of 4.6% in the head, 4.9% in the body, and 3.9% in the tail (*p* < 0.001) [112] which can be a result of confounding factors like BMI, age, and sex [113, 114]. Other studies with a limited number of patients did not demonstrate such differences [68, 80, 115]. A cutoff for pancreatic steatosis of 6.2% on MRI was suggested [116].

**FIGURE 7:**
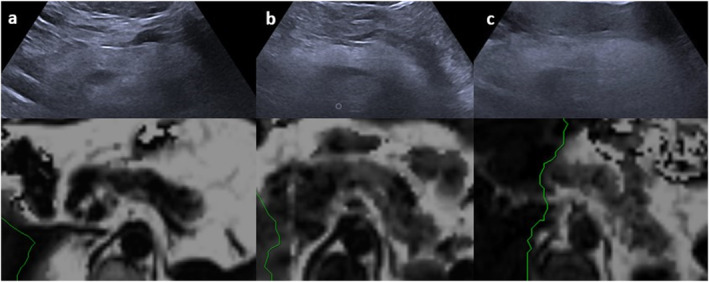
Three patients with varying grades of fatty pancreas, as assessed via transabdominal ultrasound and corresponding magnetic resonance imaging‐proton density fat fraction (MRI‐PDFF). Mild fatty pancreas with 7% pancreatic fat on MRI‐PDFF (a), moderate fatty pancreas with 20% pancreatic fat on MRI‐PDFF (b), and severe fatty pancreas with 35% pancreatic fat on MRI‐PDFF (c).

Based on clinical observations and expert opinion, fatty pancreas can be classified into three grades: mild fatty pancreas (6%–15% PDFF), moderate fatty pancreas (16%–30% PDFF), and severe fatty pancreas (over 30% PDFF) (Figure 7).

*Statement 6.4: MRI could be used for further characterization of pancreatic fat in patients with uncertain findings and suspected focal fatty pancreas* that is *not located in a characteristic area of the pancreas on ultrasonography and CT.*

*Level of evidence:* 5.

*Consensus agreement:* 86%

*Comment:* In cases where the findings on CT or US are uncertain or suggestive of potential serious focal pathology, pancreatic MRI may be helpful. Fatty pancreas can be heterogeneous and may mimic focal lesions [117] (Figures S8 and S9). This heterogeneous appearance can be further characterized by MRI with demonstration of a relatively high fat fraction in this region. Focal fatty replacement of the pancreas may occur due to intraductal calculi or tumors that obstruct the pancreatic duct [30] (Figure S10). Lipomatous pseudohypertrophy is characterized by an expansion of the pancreas in contrast to fatty infiltration or atrophy of parenchyma [118] (Figure S11). While the pathological consensus is that lipomatous pseudohypertrophy represents an extreme form of fatty pancreas, the term persists in clinical radiology to describe a morphologically distinct variant characterized by expansion of the gland, differentiating it from simple fatty pancreas or atrophy of the pancreatic parenchyma. Distal pancreatic agenesis is another entity that should be differentiated from distal fatty pancreas. In the absence of the distal pancreas, the distal pancreatic bed may be occupied by the stomach or intestine (referred to as dependent stomach or dependent intestine signs), which are adjacent to the splenic vein [119] (Figure S12). Beyond that, some pancreatic lesions may contain fat (e.g., lipoma and pancreatoblastoma) and should be evaluated comprehensively for a possible solid component [120] (Figure S13). Specific genetic disorders may also cause fatty pancreas, including cystic fibrosis, Shwachman–Diamond syndrome, and Johanson–Blizzard syndrome, the most well‐known of which is cystic fibrosis [121] (Figure S14). Indeed, further clinical research is needed to establish evidence‐based diagnostic algorithms to support clinical patient management.

*Statement 6.5: Automated artificial intelligence (AI)‐based measurement of pancreatic fat* via *MRI may enable objective, reproducible quantification, benefiting time management and large‐scale research studies. However, there is currently a lack of sufficient evidence on this subject.*

*Level of evidence:* 4.

*Consensus agreement:* 100%

*Comment:* Automated AI‐based measurement of pancreatic fat with MRI offers a promising advancement in the objective and reproducible quantification of pancreatic fat. A recently published study demonstrated that a deep learning radiomics model outperformed radiologists in providing a more efficient, accurate, and stable method for monitoring fatty pancreas [122]. Another study showed that 3D dual‐contrast nnU‐Net segmentation of the pancreas on Dixon images fully automated the assessment of pancreatic fat distribution with high reliability [123]. Moreover, the application of adapted deep convolutional neural networks for automatic measurement has shown potential for large‐scale clinical research, facilitating time‐efficient assessments across diverse populations [123, 124].

#### 2.5.7 Chapter 7

*Question:* How can fatty pancreas be diagnosed using EUS?

*Statement 7.1: EUS can be used to diagnose fatty pancreas primarily through qualitative echogenicity assessment, typically characterized by hyperechoic pancreatic parenchyma compared to the spleen or adjacent organs. Nonetheless, a correlation between hyperechogenicity and histological fat accumulation is not established, and tissue acquisition cannot be recommended.*

*Level of evidence:* 4.

*Consensus agreement:* 97%

*Comment:* Fatty pancreas can be detected via EUS as hyperechogenicity (increased echogenicity) when compared to other organs, that is, the spleen, which is less affected by fatty infiltration [50, 125, 126]. This is related to the fact that fat accumulation can increase the reflection of sound waves, making the pancreas appear brighter on the EUS view [50]. Nonetheless, echogenicity can be affected by other pathological processes, depending on the patient's body habitus and machine settings, such as the frequency, gain, and angle of insonation. Furthermore, interobserver reproducibility has not been addressed, and the technique itself cannot currently be recommended for investigating fatty pancreas due to its cost and invasiveness. Only one preliminary study has investigated fatty pancreas with EUS‐guided tissue acquisition [127]. Nonetheless, EUS‐guided tissue acquisition carries significant risks and is generally limited to differentiating malignant from benign pancreatic lesions. Its utility in fatty pancreas has not been established, and, therefore, cannot be proposed in this setting.

*Question:* How is fatty pancreas graded using EUS?

*Statement 7.2: To date, no validated EUS classification to grade the severity or the extent of fatty pancreas disease is available.*

*Level of evidence:* 4.

*Consensus agreement:* 97%

*Comment:* Currently, there is no universally accepted or validated objective method for the histological assessment of fatty pancreas, making it difficult to establish a definitive classification for grading its severity or extent using EUS. However, the current consensus panel considers this an important area for future research, as the evaluation of pancreatic fatty change is often subjective and can vary based on the endosonographer's experience and interpretation. Several attempts to classify fatty pancreas based on EUS have been proposed [50, 126, 128, 129]. In order to facilitate the development of consistent and comparable studies, we propose a preliminary classification system for fatty pancreas based on EUS findings (Table 2 and Figure S15a–Se).

**TABLE 2:** Proposal for the classification system for fatty pancreas based on endoscopic ultrasound (EUS) findings.

Extent of fatty pancreas	
No fatty pancreas	No evidence of fatty pancreas.
Segmental fatty pancreas—head	Fatty pancreas limited to the head of the pancreas; the body and tail are spared.
Segmental fatty pancreas—body and tail	Fatty pancreas involving the body and tail; the head is spared.
Diffuse fatty pancreas	Fatty pancreas involving the entire pancreas.
Severity of fatty pancreas	
No fatty pancreas	No hyperechogenicity or loss of surrounding structure definition.
Mild/moderate fatty pancreas	Hyperechoic pancreas with mild posterior acoustic attenuation; partial obscuration of anatomical landmarks (e.g., left kidney or spleen for body and tail of the pancreas, mesenteric vessels or distal duodenal wall for head of the pancreas); main pancreatic duct (MPD) mildly or moderately obscured.
Severe fatty pancreas	Markedly hyperechoic pancreas with complete loss of adjacent structure definition; MPD severely obscured, suggesting extensive fat infiltration and shadowing.

The use of a standardized EUS protocol in terms of machine settings should aim to enhance reproducibility in clinical studies. However, conventional EUS has limitations in quantifying fat content, as it is mostly a qualitative evaluation rather than a quantitative one. To minimize subjectivity and improve reliability, standardized quantitative assessments using ROI measurements of pancreatic echogenicity, compared to reference organs such as the spleen or kidney, are recommended. This approach calculates the average echogenicity ratio, enhancing reproducibility and diagnostic accuracy [126]. Furthermore, it should be noted that reduced focal fat content in the embryological ventral region is a physiological finding. Moreover, some parts of the pancreas can be spared from fat accumulation and look hypoechoic compared to the rest of the parenchyma, resembling a nodule or a tumor, which is a false positive finding.

*Question:* Can advanced imaging techniques applied in EUS be of use in fatty pancreas evaluation?

*Statement 7.3: EUS imaging analysis* via *AI and advanced imaging techniques, such as shear wave elastography (SWE), show promising potential for evaluating fatty pancreas. However, no validation on the application of these techniques has been performed.*

*Level of evidence:* 4.

*Consensus agreement:* 95%

*Comment:* Conventional EUS evaluation of fatty pancreas relies heavily on subjective qualitative judgments of tissue echogenicity, which are operator‐dependent and prone to variability. The integration of quantitative methods, such as elastography and AI, may offer measurable parameters, thereby significantly improving diagnostic accuracy. Recent studies suggest that these methods can effectively distinguish normal tissue from fatty pancreas, offering more objective assessments than traditional qualitative approaches.

SWE quantifies tissue stiffness by assessing the velocity of shear wave propagation through pancreatic tissue. Preliminary results of a recent study have suggested its feasibility and reproducibility, associating higher stiffness measurements with increased pancreatic fat infiltration [130]. Specifically, median EUS‐SWE Versus and elasticity values were significantly elevated in patients with fatty pancreas compared to those without. Furthermore, SWE Versus measurements independently predicted fatty pancreas even after adjustments for confounding factors like BMI, age, gender, race, alcohol use, and smoking history. In a subsequent study, a significant poor‐moderate correlation between median EUS‐SWE Versus values and pancreatic fat fraction assessed via MRI was observed (Pearson correlation coefficient 0.42; *p* = 0.025) [131].

The integration of AI, particularly computer‐aided detection (CADe) systems, automates the evaluation of pancreatic echogenicity, thus reducing interobserver variability and operator dependency. A recent study demonstrated that an AI model accurately detected and segmented abnormal pancreatic tissue in patients with fatty pancreas, achieving an overall accuracy of 0.93 (95% CI, 0.90–0.97) and an area under the receiver operating characteristic curve of 0.89 (95% CI, 0.85–0.93) [132].

#### 2.5.8 Chapter 8

*Question:* Is fatty pancreas a risk factor for acute pancreatitis?

*Statement 8.1: Fatty pancreas may be a risk factor for acute pancreatitis.*

*Level of evidence:* 3.

*Consensus agreement:* 88%

*Comment:* Data from cross‐sectional studies indicate that patients with acute pancreatitis (AP) are more likely to have a fatty pancreas compared to individuals without AP [46, 133, 134]. While several risk factors [4, 71, 116, 135, 136] can lead to the development of both fatty pancreas and AP, a retrospective cohort study [71] of UK Biobank participants showed that fatty pancreas was associated with a higher risk of AP compared to patients without fatty pancreas. A cohort study linked fatty pancreas to a higher risk of exocrine pancreatic disorders [137]. Moreover, a large study using Mendelian randomization suggested an association between genetically predicted fatty pancreas and AP [138]. Additionally, fatty pancreas was associated with post‐endoscopic retrograde cholangiopancreatography (ERCP) pancreatitis [72, 139, 140]. However, two retrospective cohort studies investigating AP after pancreatoduodenectomy did not observe increased adjusted odds of AP associated with fatty pancreas [141, 142].

*Statement 8.2: Fatty pancreas may be associated with increased severity of AP.*

*Level of evidence:* 4.

*Consensus agreement:* 92%

*Comment:* Many studies have reported a positive correlation between fatty pancreas and AP severity [41, 133, 143, 144, 145]. One study reported higher systemic inflammatory response scores during the first 48 h after admission in AP patients with fatty pancreas compared to those without fatty pancreas, but found no significant differences in complication rates, in‐hospital mortality, or length of hospital stay [146]. However, in post‐ERCP pancreatitis, there does not appear to be a link between fatty pancreas and the severity of AP [147, 148]. Regarding AP due to other etiologies, the available data are scarce—two studies specifically reported on acute biliary pancreatitis and suggested a positive correlation between fatty pancreas and AP severity [144, 146].

#### 2.5.9 Chapter 9

*Question:* Is there an increased risk of pancreatic exocrine insufficiency or reduced exocrine pancreatic secretion in individuals with fatty pancreas?

*Statement 9.1: The evidence regarding the association between fatty pancreas and pancreatic exocrine function is inconclusive and may be influenced by underlying pancreatic disease.*

*Level of evidence:* 4.

*Consensus agreement:* 92%

*Comment:* According to the European guidelines for the diagnosis and treatment of pancreatic exocrine insufficiency (PEI), PEI is defined as a reduction in exocrine pancreatic secretion and/or intraluminal activity of pancreatic enzymes below the level that allows the normal digestion of nutrients [6]. Only one small study has reported on the digestive capacity of the pancreas in patients with fatty pancreas using the combined ^13^C‐mixed triglyceride (MTG) breath test and dietary assessment [69]. This study reported normal breath test results and the absence of nutritional deficiencies in all cases, indicating that PEI is not present in patients with fatty pancreas. This is supported by a study reporting that the level of pancreatic fat is not associated with the digestive capacity of the pancreas as assessed using the N‐benzoyl‐L‐tyros‐p‐aminobenzoic acid test [149]. In a study of 49 insulin‐naïve patients with type 2 DM, pancreatic fat content (as measured using MRI) was not associated with reduced pancreatic function [as determined by fecal elastase‐1 (FE‐1), fecal chymotrypsin, and ^13^C‐MTG breath tests] [150]. A systematic review of studies on fatty pancreas reported an inverse correlation between the amount of pancreatic fat and FE‐1 test results [69]. A similar inverse correlation between pancreatic secretion and the amount of pancreatic fat has been reported in a more recently published German study of 1458 healthy volunteers [151] and a recent Turkish nationwide multicenter study [70]. However, the prevalence of abnormally low FE‐1 concentrations (< 200 μg/g) was similar in patients with and without fatty pancreas [70]. Finally, in a recent Indian study, all patients with chronic pancreatitis (*n* = 8) and what the authors called “total pancreatic lipomatosis” had low FE‐1 test results [152]. In patients with cystic fibrosis, a clear correlation between fat content and PEI has been described [33, 153]. These findings highlight the importance of understanding the causes of fatty pancreas in relation to pancreatic function. In summary, the evidence on the association between fatty pancreas and pancreatic function is inconclusive and should be considered in the context of the underlying pancreatic disease.

*Question:* Is there an increased risk of chronic pancreatitis (CP) in individuals with a fatty pancreas?

*Statement 9.2: Despite the evidence being limited at present, genome‐wide association studies and cohort studies suggest that a fatty pancreas is associated with the risk of chronic pancreatitis.*

*Level of evidence:* 4.

*Consensus agreement:* 94%

*Comment:* In a recent retrospective matched cohort study, patients with fatty pancreas exhibited a significantly higher incidence of CP compared with matched controls (6.1% vs 0.6%) over a mean follow‐up period of 4 years [137]. According to a further study, features of CP were found in all eight patients with severe fatty pancreas, 87% of whom had ductal dilatation and calcifications, and 62% had metabolic syndrome [150]. However, there may be a reverse causation bias as CP‐induced pancreatic atrophy may lead to fatty pancreas. In a Turkish nationwide multicenter study of 1700 volunteers, transabdominal ultrasonography for fatty pancreas and ultrasonographic SWE for pancreatic stiffness were performed [70]. Fatty pancreas was associated with increased pancreatic stiffness, which may be a sign of pancreatic fibrosis [70]; however, pancreatic stiffness alone is insufficient to diagnose CP. Recently, a study using genetic variants from genome‐wide association studies of fatty pancreas showed that genetically predicted fatty pancreas was significantly associated with AP and CP [138]. This finding supports a potential causal role of fatty pancreas in pancreatitis but causality should be proven via prospective cohort studies.

#### 2.5.10 Chapter 10

*Question:* Is there a correlation between fatty pancreas and the prevalence of intraductal papillary mucinous neoplasms (IPMN)?

*Statement 10.1: Low‐quality evidence indicates that the prevalence of fatty pancreas may be higher in patients with IPMN than in patients without IPMN.*

*Level of evidence:* 4.

*Consensus agreement:* 94%

*Comment:* The prevalence of fatty pancreas among patients with IPMN varies widely across reporting studies, ranging from 47% to 88% [96, 126, 154]. One retrospective study with a relatively large sample size reported a higher prevalence of fatty pancreas in patients with IPMN (60%) compared to those without (39%) [126]. Additionally, some retrospective studies have found that pancreatic fat content is higher in patients with IPMN than in those without [128, 155]. However, one study indicated that this difference was significant only when comparing malignant IPMN cases to benign IPMN or non‐IPMN cases, as they found no significant difference between benign IPMN and non‐IPMN cases [156]. A Japanese retrospective matched cohort study that evaluated the association between IPMN and fatty pancreas in patients undergoing MRI and CT scans for various indications also reported that the CT attenuation indices in each of the evaluated pancreatic regions (head/body/tail) were lower in patients with IPMN than in patients without pancreatic cysts [155]. In terms of IPMN subtype, a cross‐sectional study evaluating the associations between pancreato‐hepato‐biliary disorders and fatty pancreas, diagnosed via EUS, revealed that main‐duct IPMN, but not mixed‐type or branch‐duct IPMN, was significantly associated with fatty pancreas [128]. These findings should be interpreted with caution for two reasons. First, fatty replacement impacts the background attenuation on imaging, potentially leading to a higher sensitivity for cystic lesions. This detection bias may have resulted in a higher prevalence. Second, due to the retrospective design of the available studies, it is not possible to determine whether the observed association between the two entities reflects causation or correlation.

*Question:* Does fatty pancreas increase the risk of progression in IPMN?

*Statement 10.2: While fatty pancreas may be associated with an increased risk of IPMN progression, there is insufficient evidence for a definite conclusion or establishment of a causal relationship.*

*Level of evidence:* 4.

*Consensus agreement:* 88%

*Comment:* Although limited, the available data suggest that in patients with IPMN, pancreatic fat content is higher in malignant or high‐grade dysplastic lesions compared to low‐risk IPMN [155, 156, 157, 158]. Additionally, pancreatic fat content appeared to increase over time when IPMN progressed to malignancy, while remaining stable in cases without IPMN progression [158]. This is in line with studies reporting an association between metabolic syndrome and obesity and an increased risk of IPMN progression [159]. Regarding specific worrisome features and high‐risk stigmata associated with IPMN, a retrospective, single‐center study identified a correlation between fatty pancreas and main pancreatic duct diameter, cyst diameter, mural nodule size, and CA19‐9 serum levels [157].

The method of evaluating fatty pancreas varied greatly across studies. Furthermore, many published reports have focused on selected cases of resected IPMN lesions only, introducing a selection bias [154, 157]. Overall, the level of evidence is very low and insufficient to draw a definitive conclusion regarding whether fatty pancreas increases the risk of IPMN progression, and if this concerns causality rather than correlation. Currently, there is also insufficient evidence to support tailoring IPMN follow‐up strategies based on fatty pancreas.

#### 2.5.11 Chapter 11

*Question:* Is fatty pancreas a risk factor for pancreatic cancer?

*Statement 11.1: Fatty pancreas is associated with pancreatic cancer. While direct causality and the exact mechanisms remain under investigation, a growing body of evidence suggests that patients with fatty pancreas are at an increased risk of developing pancreatic cancer.*

*Level of evidence:* 3.

*Consensus agreement:* 99%

*Comment:* Several studies have explored the relationship between fatty pancreas and pancreatic cancer (PC). Although much of the existing research is based on small cohort studies and retrospective analyses, three systematic reviews have synthesized these findings. A systematic review [160] found that fatty pancreas significantly increased the risk of PC or pre‐malignant lesions (relative risk, 2.78; 95% confidence interval, [CI], 1.56–4.94; *p* < 0.001). Another article [161] reported that the likelihood of fatty pancreas among patients with PC was more than six times higher (odds ratio [OR], 6.13; 95% CI, 2.61–14.42). A recent meta‐analysis [5] estimated a pooled OR of 3.23 (95% CI, 1.86–5.60) for fatty pancreas in patients with PC compared to controls. These reviews suggest that individuals with fatty pancreas face a notably higher risk of developing PC than those without. Further supporting evidence comes from a large‐scale prospective cohort study using UK Biobank data [71]. This study found that fatty pancreas was associated with an increased hazard ratio (HR) of 1.976 (95% CI, 1.054–3.704), strengthening the argument that fatty pancreas may be an independent risk factor for PC. Another study [162] adds to the growing literature linking fatty pancreas to PC, emphasizing the need to investigate the biological mechanisms underlying this association. Nonetheless, despite these findings, the causal relationship between fatty pancreas and PC remains uncertain. Most studies establish correlation rather than direct causation. However, a recent Mendelian randomization study [163] provides compelling evidence suggesting a potential causal link. By leveraging genetic variants as instrumental variables, this study minimized confounding factors inherent in observational research, further supporting the hypothesis that fatty pancreas may contribute to PC development. Several studies have explored the mechanistic links between fatty pancreas and PC. Proposed pathophysiological mechanisms include lipotoxicity, chronic inflammation, oxidative stress, altered metabolic signaling, dysregulated autophagy, immune modulation, and activation of cellular processes such as pancreatic stellate cell activation followed by fibrosis. Collectively, these factors may contribute to a tumor‐promoting microenvironment that facilitates cancer development, progression, and metastasis [164, 165, 166].

#### 2.5.12 Chapter 12

*Question:* Is fatty pancreas a risk factor for surgical complications?

*Statement 12.1: A fatty pancreas increases the risk of surgical complications.*

*Level of evidence:* 3.

*Consensus agreement:* 98%

*Comment:* The presence of excessive fat around or within the pancreas is associated with an increased risk of adverse postoperative events following pancreatoduodenectomy (PD) [167]. A fatty pancreas may elevate the risk of surgical complications in three ways: by making surgical tissue handling more challenging, by promoting inflammation, and, indirectly, by increasing the risk of diabetes. Fat accumulation around or within the pancreas can complicate surgical procedures and impair healing, contributing to a higher incidence of severe complications such as postoperative pancreatic fistula (POPF) [89, 98, 168, 169, 170, 171]. In patients with elevated BMI, fat accumulation in the tissues surrounding the pancreas may impede the healing of the pancreatic anastomosis [91, 96, 98, 172, 173, 174, 175, 176].

Although the pancreatic attenuation index can be used to estimate fat content, its predictive value for specific outcomes, such as POPF, remains limited [177]. Histopathological analyses have shown that acinar cells play a critical role in the risk of complications. A high acinar cell count at the resection margin is predictive of postoperative complications, such as pancreatic fistulas and acute pancreatitis [142, 178, 179]. Further histological investigations on the fat content of the resection margin will be helpful in determining the impact of fatty pancreas on POPF.

A fatty pancreas is often associated with metabolic syndrome and insulin resistance, conditions that further increase surgical risk [84, 109, 180]. These systemic complications are exacerbated by heightened inflammation and elevated risk of thrombotic events, both of which can significantly impede postoperative recovery. Therefore, preoperative screening for obesity and fatty pancreas is essential [65, 181]. Moreover, effective preoperative management strategies, including glycemic control and weight reduction, may help reduce the surgical risks associated with a fatty pancreas [88, 182, 183, 184, 185, 186, 187].

#### 2.5.13 Chapter 13

*Question:* What is the prevalence and clinical significance of fatty pancreas in the pediatric population?

*Statement 13.1: The prevalence of fatty pancreas is increased in children with obesity, type 2 DM, metabolic dysfunction‐associated steatotic liver disease (MASLD), as well as in children with cystic fibrosis, Shwachman–Diamond syndrome, or Pearson syndrome.*

*Level of evidence:* 3.

*Consensus agreement:* 98%

*Comment:* Fatty pancreas has been described in children in two distinct settings. First, in association with several congenital disorders (Table 1). In patients with cystic fibrosis, clinical studies have shown that the extent of fatty pancreas was correlated to a decline in exocrine function [153]. This correlation has also been shown in animal models of Shwachman‐Diamond syndrome [188]. Clinical and translational research is needed to further investigate the pathophysiology and impact on exocrine function. Second, fatty pancreas has been reported in children with overweight or type 2 DM. Studies have highlighted varying prevalence rates of fatty pancreas in pediatric populations. In a cohort of Chilean adolescents aged 13–18 years, 4% exhibited ultrasonographic signs of fatty pancreas [189]. A South Korean study found 26.5% of children aged 5–18 years had an echogenic pancreas on abdominal ultrasound [190]. In an American pediatric tertiary care center, 11.5% of children aged 2–18 years showed signs of fatty pancreas on CT scans performed in emergency or inpatient settings [191]. Higher prevalence rates were observed in at‐risk populations. Among obese adolescents with MASLD, the prevalence of fatty pancreas was 74.5% in a South Korean study, 52% in an Italian study [192], and 50% in a Hong Kong cohort [193] using MRI to assess pancreas fat fraction. Additionally, the prevalence of fatty pancreas was 51%–75% in children with cystic fibrosis [194], 57.1% in a systematic review of patients with Shwachman–Diamond syndrome [195], and 94% of children with Pearson syndrome [196]. These studies underscore the increasing recognition of fatty pancreas in pediatric populations, particularly in those with obesity, MASLD, or specific syndromes.

*Question:* What are the clinical consequences of fatty pancreas in children?

*Statement 13.2: Fatty pancreas in children is increasingly recognized as a potential marker for metabolic syndrome, with its presence correlating with higher levels of abdominal adiposity.*

*Level of evidence:* 3.

*Consensus agreement:* 95%

*Comment:* The growing prevalence of fatty pancreas in children, particularly in the context of rising childhood obesity and metabolic disorders, has raised significant clinical concerns. Studies have shown that fatty pancreas, assessed through MRI, is an independent predictor of metabolic syndrome [193]. In children, fatty pancreas is more common among those with obesity, with its prevalence ranging from 18% to 50% [191, 193, 197]. Notably, the degree of pancreatic fat correlates more with the absolute amount of abdominal adiposity rather than its distribution [198]. A study of Chinese children revealed that the odds of metabolic syndrome were significantly higher in children with both obesity and fatty pancreas [193]. Although pancreatic fat is located near insulin‐secreting beta‐cells and forms part of the total ectopic fat [197], the association between fatty pancreas and the development of type 2 DM in children remains inconclusive. Some studies suggest that children with both obesity and fatty pancreas may be at an increased risk for insulin resistance and beta‐cell dysfunction, while others find no significant link to glucose intolerance or prediabetes [190, 193, 197, 198, 199, 200]. Moreover, the presence of fatty pancreas in children may indicate a higher lifetime risk of cardiovascular diseases [201, 202]. While fatty pancreas may not cause immediate symptoms, studies in adults suggest it could predispose individuals to pancreatitis, PEI, or PC. However, no pediatric studies have yet confirmed these risks.

#### 2.5.14 Chapter 14

*Question:* Is fatty pancreas associated with MASLD?

*Statement 14.1: Fatty pancreas is associated with the presence of MASLD.*

*Level of evidence:* 1.

*Consensus agreement:* 97%

*Comment:*. Fatty pancreas was strongly associated with MASLD when evaluated with transabdominal sonography [70, 203], CT [204], or MRI [205, 206]. The presence of fatty pancreas was associated with an increased risk of MASLD in two meta‐analysis^172136,^ Furthermore, fatty pancreas had a strong association with advanced fibrosis in patients with MASLD [207]. Fatty pancreas was a significant predictor for the presence of MASLD in a histological study [86], and this relationship seems to be mediated by general obesity. However, a recent MRI study has found no correlation between fatty pancreas and MASLD [208]. Furthermore, fatty pancreas did not fully accompany MASLD. Despite having severe fatty pancreas, more than 25% of cases had normal liver echogenicity. Patients with fatty pancreas and those with fatty liver have partially different demographic characteristics [209].

*Question:* Is fatty pancreas associated with metabolic syndrome?

*Statement 14.2: Fatty pancreas is associated with metabolic syndrome and its components, such as hypertension, hyperlipidemia, DM, and central overweight/obesity. As a consequence, fatty pancreas is associated with cardiovascular disease linked with metabolic syndrome.*

*Level of evidence:* 2.

*Consensus agreement:* 97%

*Comment:* In a meta‐analysis, fatty pancreas was associated with a significantly increased risk of having metabolic syndrome, hypertension, type 2 DM, and central obesity [180]. The association between fatty pancreas and hyperlipidemia was not significant [180]. In cross‐sectional surveys, fatty pancreas was also associated with the occurrence of metabolic syndrome, type 2 DM, and hypertension [203]. The association of fatty pancreas with hyperlipidemia [57, 64, 204, 210], type 2 DM [57, 64, 206], obesity [57, 64, 204, 205], hypertension [64, 204], and metabolic syndrome [205] was confirmed in various retrospective and prospective cohort studies. A meta‐analysis showed that fatty pancreas was significantly associated with increased aortic and carotid intima‐media thickness and increased vascular stiffness [211], which are all markers of subclinical cardiovascular disease and established risk factors for developing future clinical cardiovascular disease‐related events.

#### 2.5.15 Chapter 15

*Question:* Can fatty pancreas affect endocrine function and glucose homeostasis?

*Statement 15.1: Fatty pancreas impacts the endocrine pancreas and may contribute to impaired insulin secretion and increased diabetes risk.*

*Level of evidence:* 2.

*Consensus agreement:* 97%

*Comment:* Among 80 cross‐sectional studies identified, 80% report a significant positive association between fatty pancreas and impaired beta‐cell function, reduced insulin secretion, or the presence of prediabetes/diabetes. This association was evident across various methodologies for pancreatic fat quantification, including MRI [212], CT [213], and histological analysis [39]. Ten studies report a lack of a significant association that could not be explained by the reported study parameters [214]. One contributor to this heterogeneity is that beta‐cell susceptibility to fat‐induced suppression appears to be genetically determined [87, 215]. The association between fatty pancreas and beta‐cell function or the presence of (pre)diabetes appears to be modulated by several factors. First, stronger associations were observed in Asian populations [216], while weaker or absent associations were reported in populations with an African/Black ethnicity [217]. Second, both hepatic fat and measures of body weight are associated with fatty pancreas in most studies [26, 218]. Adjusting for these parameters reduces, but does not abolish, the relationship between fatty pancreas and beta‐cell function and (pre)diabetes, suggesting an independent impact [219]. Finally, stronger associations have been reported in individuals with a family history of DM and in males [87]. Several prospective observational studies with 3–5 years of follow‐up suggest that higher baseline pancreatic fat levels predict an increased risk of type 2 DM or accelerated glycemic deterioration, although these associations were often attenuated after adjusting for total adiposity. In a UK Biobank study, each quintile of increased pancreatic fat was associated with a 22% higher risk of developing DM over 4.6 years in more than 40,000 individuals [71]. A Japanese study found a similar association only in lean persons (BMI < 25 kg/m^2^), independent of hepatic fat and other confounders [213]. A Mendelian randomization analysis of UK Biobank data did not find a causal link between fatty pancreas and type 2 DM risk [220]. However, rare genetic syndromes have demonstrated a co‐occurrence of markedly elevated pancreatic fat with hyperglycemia or DM [221, 222, 223]. Several prospective interventional studies assessed whether a reduction of pancreatic fat is associated with improved glycemia or beta‐cell function. Overall, interventions achieving substantial weight loss, whether through dietary or bariatric surgical means, were effective in reducing fatty pancreas, which was accompanied by improvements in fasting glucose levels and insulin secretion. One study demonstrated that the reaccumulation of pancreatic fat was associated with reduced insulin secretion [31]. To date, no adequately powered trials with long follow‐up periods have specifically addressed the impact of pharmacotherapy on fatty pancreas and its metabolic consequences. In summary, while the number of confounding factors makes it difficult to assess the independent effect of pancreatic fat on beta‐cell function, multiple lines of evidence from human epidemiologic and intervention studies, alongside mechanistic studies using animal models [223, 224, 225, 226, 227, 228] and human pancreatic adipose tissue [84, 229, 230, 231] suggest that fatty pancreas impacts beta‐cell function and may contribute to DM in the context of other risk factors.

#### 2.5.16 Chapter 16

*Question:* What is the recommended approach for the treatment and monitoring of patients with fatty pancreas?

*Statement 16.1: There is no specific recommendation to treat fatty pancreas. Treatment relies on the underlying etiology. In case of metabolic syndrome, targeted lifestyle changes, bariatric surgery, and glucose‐lowering medications are effective in reducing fat in the pancreas and total pancreatic volume.*

*Level of evidence:* 1.

*Consensus agreement:* 95%

*Statement 16.2: There are currently no specific recommendations for the follow‐up of individuals with fatty pancreas. Monitoring is tailored to the underlying pancreatic diseases and associated metabolic risk factors.*

*Level of evidence:* 4.

*Consensus agreement:* 93%

*Comment:* Significant reductions in pancreatic fat (from 2% to 42%) were observed in patients administered restrictive diets, physical activities, or medications. Observational series and five randomized controlled trials confirmed the benefits of restrictive diets (low‐calorie and low‐fat diets) and physical training on fatty pancreas [232, 233, 234, 235, 236, 237, 238, 239]. The minimum duration of intervention to obtain a significant result was 6 weeks.

The types of medications tested to reduce the risk of fatty pancreas in randomized controlled trials were glucose‐lowering medications [thiazolidinediones, dipeptidyl peptidase‐4 (DPP‐4) inhibitors, glucagon‐like peptide‐1 receptor (GLP‐1) agonists, sodium‐glucose cotransporter‐2 (SGLT2) inhibitors and somatostatin receptor agonists]. In contrast, insulin, known to promote the storage of excess glucose as fat, was not expected to be beneficial in patients with pancreatic fat. One randomized placebo‐controlled trial of thiazolidinediones showed a significant reduction of fatty pancreas in preliminary results. Four randomized controlled trials investigated the potential role of GLP‐1 receptor agonists in individuals with type 2 DM. A relative reduction of fat between 10% and 23% was observed. The administration of a DPP‐4 inhibitor in a randomized placebo‐controlled trial of individuals with type 2 DM showed a relative reduction of fat of 16%. Regarding the physiopathological mechanisms, these results were associated with a decrease of the endoplasmic reticulum stress. A double‐blind, placebo‐controlled trial showed that empagliflozin, a SGLT2 inhibitor, decreases hepatic fat and fasting glucose in overweight and obese individuals with prediabetes, while pancreatic fat remains unchanged, indicating that its metabolic benefits are independent of alterations in pancreatic fat [240]. Similarly, dapagliflozin has been shown to reduce both hepatic and pancreatic fat and to improve markers of liver inflammation, including serum alanine aminotransferase, tumor necrosis factor‐α, and interleukin‐6, in individuals with type 2 diabetes [241]. A 12‐week, randomized, double‐blind, parallel‐group study comparing liraglutide and sitagliptin reported only transient, modest increases in plasma pancreatic enzyme concentrations, with pancreatic exocrine function largely preserved, apart from a minimal sitagliptin‐induced increase in intraduodenal fluid secretion [242]. However, the results of these trials are debatable because the sample sizes were small and the results were controversial [239, 242]. No effects were observed with thiazolidinediones, fibrates or sulfonylureas.

Metabolic (or bariatric) surgery has shown a significant decrease in pancreatic fat post‐surgery (from 26% to 67%) in all investigated observational studies [237, 243, 244, 245, 246]. The median follow‐up periods were 6–12 months. Nonetheless, the relationship between weight loss and fat reduction remains questionable as their association has not yet been clearly demonstrated, despite fat deposition being associated with obesity and despite all patients losing weight after surgery [243, 244, 245, 246]. No data dealing with treatment approaches for fatty pancreas as a consequence of hereditary diseases or pancreatic tumors exists. Therefore, we can assume that the pathophysiological process surrounding this procedure is different and that the rationale for using medical and surgical approaches is not valid.

Furthermore, the role of fatty pancreas in oncogenesis was also investigated, and a significant relationship was confirmed between pancreatic fat and both pancreatic inflammation and PC or pre‐malignant lesions (PanIN, intraductal papillary mucinous neoplasm). However, this potential risk factor and the relative risk of cancer associated with fatty pancreas have not yet been determined to be indications for cancer screening [1, 2, 17, 99, 160, 247, 248].

## 3 Conclusion

This international multidisciplinary consensus report represents the first comprehensive effort to define and characterize fatty pancreas. In summary, fatty pancreas may be defined as an accumulation of fat due to various etiologies but an unknown pathophysiology, and may be diagnosed via imaging and EUS. The hallmark of fatty pancreas is the presence of intralobular and/or extralobular adipocytes. However, there is currently no universally accepted, validated objective method for histologically assessing the severity of fatty pancreas. A major achievement of this initiative was the agreement on standardized terminology, with “fatty pancreas” being established as the preferred and inclusive term. Consensus was also reached on key diagnostic imaging findings, including radiological and endoscopic features, providing a necessary framework for consistent clinical evaluation and future research. Both the MRI‐PDFF‐based severity grading and the proposed EUS classification represent consensus‐based expert recommendations rather than evidence‐validated thresholds. These grading systems should be considered preliminary and hypothesis‐generating tools that aim to provide a foundation for future studies, rather than definitive clinical criteria at this stage.

Beyond terminology and diagnostics, this report reviews current evidence across a wide spectrum of topics. These include the etiology and epidemiology of fatty pancreas, as well as its associations with alcohol use and smoking. The panel also addressed its potential role in AP and CP, PEI, and surgical complications. Moreover, the relationship between fatty pancreas and IPMN, PC, and metabolic conditions (such as MASLD, metabolic syndrome, and impaired beta‐cell function) was evaluated. The prevalence and implications of fatty pancreas in pediatric populations were also considered.

Importantly, this report highlights substantial gaps in evidence, particularly a lack of prospective, high‐quality clinical studies (Table 3). These findings underscore the urgent need for further research to clarify the clinical significance of fatty pancreas and to develop evidence‐based strategies for the treatment and monitoring of patients with this condition. We hope that this consensus will serve as a foundational reference to guide future research and promote the development of evidence‐based clinical guidelines in the field of fatty pancreas.

**TABLE 3:** Summary of key knowledge gaps and unmet research needs.

Domain	Knowledge gaps and unmet research needs
Etiology	• The precise cellular origin of intrapancreatic adipocytes remains unclear, with competing hypotheses (e.g., cellular transdifferentiation, stem cell differentiation, adipocyte precursor infiltration) requiring further validation in human and animal models. • The relative contributions of metabolic, genetic, inflammatory, and obstructive factors in the pathogenesis of fatty pancreas are not well quantified, especially in non‐obese individuals. • The current understanding of the mechanistic links between fatty pancreas and type 2 diabetes mellitus is limited, particularly regarding reversibility following metabolic improvement. • Most etiological data are derived from animal studies; there is a need for longitudinal human studies to confirm the proposed mechanisms of adipocyte accumulation and fat replacement in the pancreas. • A standardized etiological classification (distinguishing metabolic, anatomical, and genetic contributors) is needed to guide research and clinical practice.
Alcohol and smoking	• Current evidence from human studies is limited, heterogeneous, and primarily cross‐sectional; robust longitudinal studies are needed to clarify causal relationships. • The independent and combined effects of alcohol consumption and smoking on fatty pancreas remain poorly defined, especially in populations without metabolic syndrome. • There is a lack of mechanistic studies exploring how alcohol or smoking might influence the development of fatty pancreas or fat redistribution at the cellular level. • No studies have systematically assessed whether reducing or stopping alcohol and smoking can prevent the onset or progression of fatty pancreas. • Further research is needed to determine whether alcohol and smoking act as modifiers in specific subgroups (e.g., those with pancreatitis, genetic predisposition, or high visceral adiposity).
Epidemiology	• The absence of standardized diagnostic criteria across imaging modalities and studies leads to substantial variability in reported prevalence rates. • Reliable population‐level prevalence estimates are lacking due to heterogeneity in study design, patient selection, and regional data gaps. • The influence of demographic and metabolic risk factors on prevalence (e.g., age, sex, body mass index, metabolic syndrome) remains inconsistently reported and inadequately stratified. • Few studies have systematically assessed the epidemiology of fatty pancreas in non‐obese, younger, or ethnically diverse populations. • There is a need for large, prospective, population‐based studies using harmonized diagnostic definitions and imaging protocols to better characterize the associations between prevalence and risk factors.
Histopathology	• A systematic characterization of the histomorphology of fatty pancreas, including the distribution of fatty infiltration and changes other than adipocyte infiltration (e.g., fibrosis and inflammation), in patients with various underlying conditions is lacking. • There is no commonly accepted or validated histological grading system for fatty pancreas severity. • Most human histological data are derived from surgical specimens, which may be biased by underlying pathology or surgical technique. • There is an unmet need for automated, quantitative histological analysis tools to enable reproducible assessment and to correlate findings with imaging, clinical, and prognostic features.
Radiology	• Standardized diagnostic criteria for transabdominal ultrasound are lacking. Current grading systems lack validation, and interobserver variability remains high due to technical and anatomical limitations. • Optimal computed tomography (CT) protocols for detecting mild fatty pancreas are undefined. While hounsfield unit (HU) measurements show promise, variations in scanner parameters and contrast dosing reduce reproducibility, and “invisible fat” remains a diagnostic challenge. • Magnetic resonance imaging‐proton density fat fraction (MRI‐PDFF) is the gold standard; however, it lacks universal cutoff values for clinical significance. Proposed thresholds require validation in diverse populations and correlation with long‐term outcomes. • The clinical utility of artificial intelligence (AI)‐based quantification tools is not yet established. Despite promising results in automated fat measurement, multicenter validation and integration into routine practice are needed.
Endoscopic ultrasound	• No validated endoscopic ultrasound (EUS) classification system exists for staging fatty pancreas severity. Current qualitative assessments (e.g., hyperechogenicity) lack standardization, and proposed grading systems require multicenter validation. • Interobserver variability in EUS‐based diagnosis remains unaddressed. Subjective echogenicity interpretation is influenced by operator experience and machine settings, limiting reproducibility. • Advanced EUS technologies [e.g., shear wave elastography (SWE) and AI] lack clinical validation. • The role of EUS in differentiating focal fat from neoplasms is undefined. False‐positive hypoechoic “lesions” due to heterogeneous fat distribution warrant evidence‐based imaging algorithms.
Acute pancreatitis (AP)	• The causal relationship between fatty pancreas and AP remains unproven. While observational studies and mendelian randomization suggest an association, confounding metabolic factors require further adjustment in prospective cohorts. • The impact of fatty pancreas on AP severity is inconsistently reported. Discrepancies exist across etiologies (e.g., post‐ERCP vs. biliary AP), and data on complications/mortality are limited. • Mechanisms linking the development of fatty pancreas to AP pathogenesis are unclear. Potential roles of lipotoxicity, inflammation, or adipokine dysregulation need experimental validation. • The influence of fat distribution (diffuse vs. focal) on AP susceptibility is unstudied. Heterogeneous patterns of fatty pancreas may differentially affect ductal obstruction or local inflammation.
Chronic pancreatitis and pancreatic exocrine insufficiency (PEI)	• The relationship between fatty pancreas and PEI remains unclear. • The impact of fat quantity and distribution on exocrine function is undefined. Thresholds of pancreatic fat content associated with clinically significant PEI are lacking. • Longitudinal data on fatty pancreas progression to chronic pancreatitis are absent. • Development of non‐invasive diagnostic tests for detecting fatty pancreas. • Development of clinical scoring systems to predict the presence or risk of fatty pancreas.
Intraductal papillary mucinous neoplasms (IPMN)	• The nature of the association between fatty pancreas and IPMN remains unclear. • The role of fatty pancreas in IPMN progression is not established. Limited data suggest that fat content may increase with malignant progression; however, the currently available evidence is conflicting. Longitudinal studies tracking fat quantity and dysplasia grade over time are missing. • Mechanistic links (e.g., lipotoxicity, inflammation) are unexplored. Hypotheses include adipokine‐driven tumorigenesis or ductal obstruction by fat, but experimental models are lacking. • Clinical implications of fatty pancreas for IPMN surveillance are undefined. Current data are insufficient to justify modifying follow‐up strategies based on fatty pancreas.
Pancreatic cancer	• The causal relationship between fatty pancreas and pancreatic cancer remains unproven. Experimental models are necessary to investigate lipotoxicity, chronic inflammation, or adipokine‐driven carcinogenesis. • Thresholds of fatty pancreas that confer cancer risk are undefined. • The role of fat distribution (diffuse vs. focal) in pancreatic cancer pathogenesis is unstudied. Regional fat accumulation near ducts or lesions may differentially promote tumorigenesis. • Confounding by metabolic comorbidities is incompletely addressed. Large cohorts with adjusted analyses are required to isolate fat‐specific effects. • The clinical utility of fatty pancreas as a pancreatic cancer screening marker remains uncertain. Prospective validation of its predictive value alongside established risk factors is warranted.
Surgical complications	• The impact of fatty pancreas on specific surgical complications requires clarification. While fatty pancreas is associated with postoperative pancreatic fistula (POPF) and anastomotic leaks, the relative contributions of fat infiltration versus acinar cell loss remain undefined. • Standardized preoperative imaging criteria for predicting surgical risk are lacking. • The mechanisms linking fatty pancreas to poor healing are unproven. Hypotheses include impaired tissue integrity, metabolic dysfunction (e.g., insulin resistance), and chronic inflammation, but experimental validation is needed. • Optimal preoperative optimization strategies for fatty pancreas patients are undefined. While weight loss and glycemic control are proposed, evidence‐based protocols are absent. • The role of fat distribution (peripancreatic vs. intraparenchymal) in complications is unstudied. Regional fatty pancreas may differentially affect anastomotic healing.
Pediatric fatty pancreas	• Standardized diagnostic criteria for pediatric fatty pancreas are lacking. It is important to establish age‐specific imaging cutoffs. • The long‐term metabolic consequences of childhood fatty pancreas remain undefined. While associated with metabolic syndrome and abdominal adiposity, causal links to insulin resistance, type 2 diabetes mellitus, and cardiovascular disease require longitudinal studies. • Syndrome‐specific risk stratification is absent. The clinical significance of fatty pancreas in cystic fibrosis, Shwachman–Diamond syndrome, and Pearson syndrome (where prevalence exceeds 50%) is poorly characterized. Prospective cohorts tracking pancreatic function and comorbidities are necessary. • Mechanisms driving fat accumulation are unstudied. Developmental differences in adipogenesis, inflammation, or genetic susceptibility may exist, but lack experimental validation.
Metabolic dysfunction‐associated steatotic liver disease (MASLD) and metabolic syndrome	• The directionality of the fatty pancreas‐MASLD association remains unclear. While strong correlations exist, it is unknown whether fatty pancreas drives MASLD progression or vice versa. • Demographic differences in pancreas‐liver fat discordance are unexplained. • The clinical utility of fatty pancreas as a metabolic syndrome marker is unproven. Prospective validation is necessary to determine whether pancreatic fat quantification enhances risk prediction beyond established criteria.
Beta‐cell function and glucose homeostasis	• The causal role of fatty pancreas in beta‐cell dysfunction remains debated. Large‐scale genetic studies using tissue‐specific instruments are needed to clarify causality. • Ethnic and sex‐specific susceptibility to fat‐induced beta‐cell impairment is unexplained. Stronger associations in Asian populations and males suggest the presence of genetic or hormonal modifiers that require further investigation. • Long‐term data on pharmacotherapy for fatty pancreas are lacking. No trials have evaluated diabetes medications specifically targeting pancreatic fat reduction. • Mechanisms linking fat accumulation to beta‐cell failure are incompletely understood. Potential pathways (e.g., lipotoxicity, inflammation) require validation in human pancreatic samples or imaging‐biomarker studies.
Treatment and monitoring	• Evidence‐based guidelines for fatty pancreas management are lacking. Current approaches are extrapolated from metabolic syndrome treatments; however, pancreas‐specific protocols (e.g., fat‐reduction targets, monitoring intervals) remain undefined. • The efficacy of interventions varies widely across studies. While lifestyle changes and bariatric surgery reduce pancreatic fat (2%–67%), optimal duration/intensity and long‐term sustainability are unestablished. Studies comparing dietary, pharmacological, and surgical strategies are needed. • Standardized monitoring protocols are absent. Development of risk‐adapted algorithms based on etiology and comorbidities are needed.

Abbreviations: AP, Acute pancreatitis; IPMN, Intraductal papillary mucinous neoplasms; MASLD, Metabolic dysfunction‐associated steatotic liver disease; PEI, pancreatic exocrine insufficiency.

## Funding

Acibadem Mehmet Ali Aydinlar University, Istanbul, Turkey. Swedish Society for Development of Pancreatology (SweSUP), Stockholm, Sweden.

## Conflicts of Interest

Enclosed as a supplementary material.

## Supporting information


Supporting Information S1

Supporting Information S2

Supporting Information S3

**Figure S4:** Accumulation of adipocytes both within lobules (intralobular; *arrows*) and in the interlobular space (extralobular; *asterisks*).

**Figure S5:** Rare occurrence of adipocytes inside an islet of Langerhans (*asterisk*). Some intralobular adipocytes (*arrows*) are also present.

**Figure S6:** Sparse remnants of acinar parenchyma (a; *arrows*) and islets of Langerhans (b; *arrows*) amid sheets of adipocytes in advanced fatty pancreas.

**Figure S7:** Pancreatic parenchyma with fatty pancreas‐related changes and surrounding peripancreatic fat (a). A line connecting the most peripheral (remnants of) parenchyma demarcates peripancreatic fat (yellow) from extralobular fat (green; intralobular fat: red) (b).

**Figure S8:** Different types of fatty pancreas in some patients: (a) more pronounced at the head and body, (b) due to focal pancreatitis sequela in the body, (c) patchy nodular fat in the distal pancreas.

**Figure S9:** Focal fat in the pancreatic head (*) simulating a mass on computed tomography (a), in‐phase (b), and opposed‐phase (c) images. A signal drop is consistent with the presence of focal fat.

**Figure S10:** Distal fatty pancreas due to a proximal neuroendocrine tumor in the pancreatic body (white arrow).

**Figure S11:** Lipomatous pseudohypertrophy of the pancreas with enlargement of the pancreatic tissue with adipose tissue (white arrows).

**Figure S12:** Distal pancreatic agenesis and a dependent stomach. The splenic vein is shown touching the stomach.

**Figure S13:** Patient with a pancreatic fat‐containing mass (*) on computed tomography (a, b) with a solid component diagnosed with pancreatoblastoma. In in‐phase (c) and opposed‐phase (d) images, there is no signal drop due to the presence of microscopic fat.

**Figure S14:** Two patients with cystic fibrosis (a) and Schwachman–Diamond Syndrome (b) with diffuse fatty pancreas imaged using computed tomography.

**Figure S15**a: Shown are (a) endoscopic ultrasound (EUS)‐normal pancreas, (e) EUS‐mild‐to‐moderate fatty pancreas (body and tail).

**Figure S15b:** (b) EUS‐mild‐to‐moderate fatty pancreas (head).

**Figure S15c:** (c) EUS‐severe fatty pancreas (head).

**Figure S15d:** (d) EUS‐normal pancreas.

**Figure S15e:** (e) EUS‐mild‐to‐moderate fatty pancreas (body and tail).

**Figure S15f:** (f) EUS‐severe fatty pancreas (body and tail).

**Table S1:** Participating societies.

**Table S2:** Overview of the working groups.

## Data Availability

Data sharing not applicable to this article as no datasets were generated or analyzed during the current study.
