# Supplementary material for: International Multidisciplinary Consensus Report on Definitions, Diagnostic Criteria, and Management of Fatty Pancreas: A Joint Statement Endorsed by EPC, APA, EASD, EASL, ESGAR, ESGE, ESP, ESPCG, ESPEN, ESPGHAN, IAP, JPS, KPBA, LAPSG, and UEG
Source: United European Gastroenterol J. 2026 Feb 14;14(1):e70185. doi: 10.1002/ueg2.70185 (PMC12906299; doi:10.1002/ueg2.70185)
Supplement: Supplementary file 1 — Supporting Information S1 [file UEG2-14-e70185-s008.pdf]

DISCLOSURE STATEMENT

EPC POSITION STATEMENT ON FATTY PANCREAS

Type of affiliation / financial interest during the past 3 years: I

X I have no potential conflict of interests to report.

☐ I have the following potential conflict(s) of interest to report:

Receipt of grants/research supports:	
Receipt of advisory, honoraria or consultation fees:	
Participation in a company sponsored speaker's bureau:	
Stock shareholder:	
Spouse/partner:	
Other support (please specify):	

Name: Minoti Apte

Signature: Minoti Apte

Date: 4 April 2025

DISCLOSURE STATEMENT

EPC POSITION STATEMENT ON FATTY PANCREAS

Type of affiliation / financial interest during the past 3 years:

☑ I have no potential conflict of interests to report.

☐ I have the following potential conflict(s) of interest to report:

Receipt of grants/research supports:	
Receipt of advisory, honoraria or consultation fees:	
Participation in a company sponsored speaker's bureau:	
Stock shareholder:	
Spouse/partner:	
Other support (please specify):	

Name: Arantza Fariña Sarasqueta

Signature: [signature]

Date: 28 January 2025

DISCLOSURE STATEMENT

EPC POSITION STATEMENT ON FATTY PANCREAS

Type of affiliation / financial interest during the past 3 years:

☑ I have no potential conflict of interests to report.

☐ I have the following potential conflict(s) of interest to report:

Receipt of grants/research supports:	
Receipt of advisory, honoraria or consultation fees:	
Participation in a company sponsored speaker's bureau:	
Stock shareholder:	
Spouse/partner:	
Other support (please specify):	

Name: LIVIA ARCHIBUGI

Signature: Livia Archibugi

Date: JANUARY 29TH 2025

E·P·C
European Pancreatic Club
Founded 1965

DISCLOSURE STATEMENT

EPC POSITION STATEMENT ON FATTY PANCREAS

Type of affiliation / financial interest during the past 3 years:

☒ I have no potential conflict of interests to report.

☐ I have the following potential conflict(s) of interest to report:

Receipt of grants/research supports:	—
Receipt of advisory, honoraria or consultation fees:	—
Participation in a company sponsored speaker's bureau:	—
Stock shareholder:	—
Spouse/partner:	—
Other support (please specify):	—

Name: Aslihan Yavas

Signature: [signature]

Date: 28.01.25

DISCLOSURE STATEMENT

EPC POSITION STATEMENT ON FATTY PANCREAS

Type of affiliation / financial interest during the past 3 years:

☑ I have no potential conflict of interests to report.

☐ I have the following potential conflict(s) of interest to report:

Receipt of grants/research supports:	
Receipt of advisory, honoraria or consultation fees:	
Participation in a company sponsored speaker's bureau:	
Stock shareholder:	
Spouse/partner:	
Other support (please specify):	

Name: Mihailo Bezmarević

Signature: [signature]

Date: January 29, 2025

E•P•C
European Pancreatic Club
Founded 1965

DISCLOSURE STATEMENT

EPC POSITION STATEMENT ON FATTY PANCREAS

Type of affiliation / financial interest during the past 3 years:

☐ I have no potential conflict of interests to report.

☐ I have the following potential conflict(s) of interest to report:

Receipt of grants/research supports:	None.
Receipt of advisory, honoraria or consultation fees:	None.
Participation in a company sponsored speaker's bureau:	None.
Stock shareholder:	None.
Spouse/partner:	My spouse is an employee of Novartis
Other support (please specify):	None.

Name: Stefanos Bonovas

Signature: [signature]

Date: 30 JANUARY 2025

DISCLOSURE STATEMENT

EPC POSITION STATEMENT ON FATTY PANCREAS

Type of affiliation / financial interest during the past 3 years:

☒ I have no potential conflict of interests to report.

☐ I have the following potential conflict(s) of interest to report:

Receipt of grants/research supports:	
Receipt of advisory, honoraria or consultation fees:	
Participation in a company sponsored speaker's bureau:	
Stock shareholder:	
Spouse/partner:	
Other support (please specify):	

Name: Prof. Rickmer Braren, MD

Signature: Braren

Date: January 28th, 2025

E·P·C
European Pancreatic Club
Founded 1965

DISCLOSURE STATEMENT

EPC POSITION STATEMENT ON FATTY PANCREAS

Type of affiliation / financial interest during the past 3 years:

☐ I have no potential conflict of interests to report.

☒ I have the following potential conflict(s) of interest to report:

Receipt of grants/research supports:	/
Receipt of advisory, honoraria or consultation fees:	Boehringer Ingelheim, Chiesi/Amryt
Participation in a company sponsored speaker's bureau:	Lilly, Boehringer Ingelheim, Novo Nordisk Novartis, Sanofi, Astra Zeneca, Chiesi
Stock shareholder:	/
Spouse/partner:	/
Other support (please specify):	/

Name: Prof. Dr. M. Heni

Signature: [signature]

Date: 30.01.2025

European Pancreatic Club
Founded 1965

DISCLOSURE STATEMENT

EPC POSITION STATEMENT ON FATTY PANCREAS

Type of affiliation / financial interest during the past 3 years:

☑ I have no potential conflict of interests to report.

☐ I have the following potential conflict(s) of interest to report:

Receipt of grants/research supports:	
Receipt of advisory, honoraria or consultation fees:	
Participation in a company sponsored speaker's bureau:	
Stock shareholder:	
Spouse/partner:	
Other support (please specify):	

Name: BUNDUC STEFANIA

Signature:

Date: 30.01.2025

DISCLOSURE STATEMENT

EPC POSITION STATEMENT ON FATTY PANCREAS

Type of affiliation / financial interest during the past 3 years:

☐ I have no potential conflict of interests to report.

☑ I have the following potential conflict(s) of interest to report:

Receipt of grants/research supports:	
Receipt of advisory, honoraria or consultation fees:	Amgen, Viatris, Pangenix, Boston Sci, Dr Falk
Participation in a company sponsored speaker's bureau:	
Stock shareholder:	
Spouse/partner:	
Other support (please specify):	

Name: Gabriele Capurso

Signature: Gabriele capurso

Date: April 1st 2025

E·P·C
European Pancreatic Club
Founded 1965

DISCLOSURE STATEMENT

EPC POSITION STATEMENT ON FATTY PANCREAS

Type of affiliation / financial interest during the past 3 years:

☐ I have no potential conflict of interests to report.

☑ I have the following potential conflict(s) of interest to report:

Receipt of grants/research supports:	TÜBİTAK / TÜSEB
Receipt of advisory, honoraria or consultation fees:	–
Participation in a company sponsored speaker's bureau:	–
Stock shareholder:	–
Spouse/partner:	–
Other support (please specify):	–

Name: Güralp Onur Ceylan

Signature:

Date: 02.04.2025

European Pancreatic Club (EPC) | Wickenburggasse 1 | 1080 Vienna, Austria
General Secretary: Dr. Gabriele Capurso | Treasurer: Professor Heiko Witt | President 2025: Professor Irene Esposito
Email: info@europeanpancreaticclub.org | www.europeanpancreaticclub.org | Registered Austrian Association: ZVR 160 370 6974
Erste Bank: IBAN AT11 2011 1842 1568 3400 | BIC GIBAATWWXXX | Account name: European Pancreatic Club

European Pancreatic Club
Founded 1965

DISCLOSURE STATEMENT

EPC POSITION STATEMENT ON FATTY PANCREAS

Type of affiliation / financial interest during the past 3 years:

☑ I have no potential conflict of interests to report.

☐ I have the following potential conflict(s) of interest to report:

Receipt of grants/research supports:	
Receipt of advisory, honoraria or consultation fees:	
Participation in a company sponsored speaker's bureau:	
Stock shareholder:	
Spouse/partner:	
Other support (please specify):	

Name: Manil Dinesh Chouhan

Signature: [signature]

Date: 29th January 2025

European Pancreatic Club (EPC) | Wickenburggasse 1 | 1080 Vienna, Austria
General Secretary: Dr. Gabriele Capurso | Treasurer: Professor Heiko Witt | President 2025: Professor Irene Esposito
Email: info@europeanpancreaticclub.org | www.europeanpancreaticclub.org | Registered Austrian Association: ZVR 160 370 6974
Erste Bank: IBAN AT11 2011 1842 1568 3400 | BIC GIBAATWWXXX | Account name: European Pancreatic Club

E·P·C
European Pancreatic Club
Founded 1965

DISCLOSURE STATEMENT

EPC POSITION STATEMENT ON FATTY PANCREAS

Type of affiliation / financial interest during the past 3 years:

☒ I have no potential conflict of interests to report.

☐ I have the following potential conflict(s) of interest to report:

Receipt of grants/research supports:	
Receipt of advisory, honoraria or consultation fees:	
Participation in a company sponsored speaker's bureau:	
Stock shareholder:	
Spouse/partner:	
Other support (please specify):	

Name: Anne Couvelard

Pr A. COUVELARD
Département de Pathologie
Hôpital BICHAT
Paris 75877 Cedex 18
Tél. 01 40 25 80 03

Signature:

Date: 26/02/2025

DISCLOSURE STATEMENT

EPC POSITION STATEMENT ON FATTY PANCREAS

Type of affiliation / financial interest during the past 3 years:

☒ I have no potential conflict of interests to report.

☐ I have the following potential conflict(s) of interest to report:

Receipt of grants/research supports:	
Receipt of advisory, honoraria or consultation fees:	
Participation in a company sponsored speaker's bureau:	
Stock shareholder:	
Spouse/partner:	
Other support (please specify):	

Name: Pr Jerome Cros

Signature: Jerome Cros

Date: 08/02/2025

European Pancreatic Club
Founded 1965

DISCLOSURE STATEMENT

EPC POSITION STATEMENT ON FATTY PANCREAS

Type of affiliation / financial interest during the past 3 years:

☒ I have no potential conflict of interests to report.

☐ I have the following potential conflict(s) of interest to report:

Receipt of grants/research supports:	
Receipt of advisory, honoraria or consultation fees:	
Participation in a company sponsored speaker's bureau:	
Stock shareholder:	
Spouse/partner:	
Other support (please specify):	

Name: LASZLO CZAKO

Signature: [signature]

Date: Febr. 1., 2025

DISCLOSURE STATEMENT

EPC POSITION STATEMENT ON FATTY PANCREAS

Type of affiliation / financial interest during the past 3 years:

☒ I have no potential conflict of interests to report.

☐ I have the following potential conflict(s) of interest to report:

Receipt of grants/research supports:	
Receipt of advisory, honoraria or consultation fees:	
Participation in a company sponsored speaker's bureau:	
Stock shareholder:	
Spouse/partner:	
Other support (please specify):	

Name: Daniel de la Iglesia Garcia

Signature:

Date: 29/01/2025

DISCLOSURE STATEMENT

EPC POSITION STATEMENT ON FATTY PANCREAS

Type of affiliation / financial interest during the past 3 years:

☒ I have no potential conflict of interests to report.

☐ I have the following potential conflict(s) of interest to report:

Receipt of grants/research supports:	
Receipt of advisory, honoraria or consultation fees:	
Participation in a company sponsored speaker's bureau:	
Stock shareholder:	
Spouse/partner:	
Other support (please specify):	

Name: Daniel de la Iglesia Garcia

Signature:

Date: 29/01/2025

DISCLOSURE STATEMENT

EPC POSITION STATEMENT ON FATTY PANCREAS

Type of affiliation / financial interest during the past 3 years:

☐ I have no potential conflict of interests to report.

☑ I have the following potential conflict(s) of interest to report:

Receipt of grants/research supports:	Abbott
Receipt of advisory, honoraria or consultation fees:	Abbott, Abbvie, Janssen
Participation in a company sponsored speaker's bureau:	
Stock shareholder:	
Spouse/partner:	
Other support (please specify):	

Name: Enrique de-Madaria

Signature:

Date: January, 28th 2025

DISCLOSURE STATEMENT

EPC POSITION STATEMENT ON FATTY PANCREAS

Type of affiliation / financial interest during the past 3 years:

X⃠ I have no potential conflict of interests to report.

☐ I have the following potential conflict(s) of interest to report:

Receipt of grants/research supports:	DFG; German Cancer Aid, Sander Foundation
Receipt of advisory, honoraria or consultation fees:	None
Participation in a company sponsored speaker's bureau:	None
Stock shareholder:	None
Spouse/partner:	None
Other support (please specify):	Not applicable

Name: Ihsan Ekin Demir

Signature:

Date: 25th Feb 2025

DISCLOSURE STATEMENT

EPC POSITION STATEMENT ON FATTY PANCREAS

Type of affiliation / financial interest during the past 3 years:

☐ I have no potential conflict of interests to report.

☐ I have the following potential conflict(s) of interest to report:

Receipt of grants/research supports:	Unrestricted research grant from Viatris
Receipt of advisory, honoraria or consultation fees:	-
Participation in a company sponsored speaker's bureau:	Viatris, Abbott Pharmaceuticals
Stock shareholder:	-
Spouse/partner:	-
Other support (please specify):	

Name: J. Enrique Domínguez-Muñoz

Signature: JUAN ENRIQUE DOMINGUEZ MUÑOZ - 08962739F Firmado digitalmente por JUAN ENRIQUE DOMINGUEZ MUÑOZ - 08962739F Fecha: 2025.04.07 10:42:53 +02'00'

Date: 07/04/2025

DISCLOSURE STATEMENT

EPC POSITION STATEMENT ON FATTY PANCREAS

Type of affiliation / financial interest during the past 3 years:

☐ I have no potential conflict of interests to report.

■ I have the following potential conflict(s) of interest to report:

Receipt of grants/research supports:	
Receipt of advisory, honoraria or consultation fees:	I consult for CAMURUS , proceeds go to the Amsterdam UMC
Participation in a company sponsored speaker's bureau:	
Stock shareholder:	
Spouse/partner:	
Other support (please specify):	

Name: Joost PH Drenth

Signature:

Date: 18-2-2025

Erste Bank: IBAN AT11 2011 1842 1568 3400 | BIC GIBAATWWXXX | Account name: European Pancreatic Club

DISCLOSURE STATEMENT

EPC POSITION STATEMENT ON FATTY PANCREAS

Type of affiliation / financial interest during the past 3 years:

☒ I have no potential conflict of interests to report.

☐ I have the following potential conflict(s) of interest to report:

Receipt of grants/research supports:	
Receipt of advisory, honoraria or consultation fees:	
Participation in a company sponsored speaker's bureau:	
Stock shareholder:	
Spouse/partner:	
Other support (please specify):	

Name: ASBJØRN MOHR DREWES

Signature:

Date: 28.01.2025

E·P·C
European Pancreatic Club
Founded 1965

DISCLOSURE STATEMENT

EPC POSITION STATEMENT ON FATTY PANCREAS

Type of affiliation / financial interest during the past 3 years:

☐ I have no potential conflict of interests to report.

☒ I have the following potential conflict(s) of interest to report:

Receipt of grants/research supports:	Viatris, limited research grant
Receipt of advisory, honoraria or consultation fees:	Viatris, consultation fee
Participation in a company sponsored speaker's bureau:	Viatris, lecture fee
Stock shareholder:	
Spouse/partner:	
Other support (please specify):	

Name: Trond Engjom
Seksjonsoverlege, PhD
Seksjon for fordøyelsessjukdomar
Haukeland universitetssjukehus
HPR: 7070241

Signature: T. E[illegible]

Date: 26/2-25

E·P·C
European Pancreatic Club
Founded 1985

DISCLOSURE STATEMENT

EPC POSITION STATEMENT ON FATTY PANCREAS

Type of affiliation / financial interest during the past 3 years:

☑ I have no potential conflict of interests to report.

☐ I have the following potential conflict(s) of interest to report:

Receipt of grants/research supports:	
Receipt of advisory, honoraria or consultation fees:	
Participation in a company sponsored speaker's bureau:	
Stock shareholder:	
Spouse/partner:	
Other support (please specify):	

Name: PIERLUIGI FRACASSO

Signature: Pierluigi Fracasso

Date: 13. February 2025

DISCLOSURE STATEMENT

EPC POSITION STATEMENT ON FATTY PANCREAS

Type of affiliation / financial interest during the past 3 years:

☐ I have no potential conflict of interests to report.

☐ I have the following potential conflict(s) of interest to report:

Receipt of grants/research supports:	Research Foundation Flanders (FWO) (1802154N)<br>Astellas, Falk Pharma, Genfit, Gilead Sciences, GlympsBio, Janssens Pharmaceutica, Inventiva, Merck Sharp & Dome, Pfizer, Roche<br>Doctoral grant University of Antwerp, BOF, Antigoon ID: 44430<br>Research Grant Belgian Association for the Study of the Liver<br>Senior clinical investigator fellowship Research Foundation Flanders (FWO): 1802154N
Receipt of advisory, honoraria or consultation fees:	Abbvie, Actelion, Aelin Therapeutics, AgomAb, Aligos Therapeutics, Allergan, Alnylam, Astellas, Astra Zeneca, Bayer, Boehringer Ingelheim, Bristoll-Meyers Squibb, CSL Behring, Coherus, Echosens, dr. Falk Pharma, Eisai, Enyo, Galapagos, Galmed, Genetech, Genfit, Genflow Biosciences, Gilead Sciences, Intercept, Inventiva, Janssens Pharmaceutica, Pro. Med. CS Praha, Julius Clinical, Madrigal, Medimmune, Merck Sharp & Dome, Mursla, NGM Bio, Novartis, Novo Nordisk, Promethera, Roche, Siemens Healthineers, Weatherden
Participation in a company sponsored speaker's bureau:	Abbvie, Allergan, Bayer, Eisai, Genfit, Gilead Sciences, Janssens Cilag, Intercept, Inventiva, Merck Sharp & Dome, Novo Nordisk, Promethera, Siemens
Stock shareholder:	/
Spouse/partner:	/
Other support (please specify):	/

Name: Prof. dr. Sven Francque
Signature:
Date: 30/01/2025

E·P·C
European Pancreatic Club
Founded 1965

DISCLOSURE STATEMENT

EPC POSITION STATEMENT ON FATTY PANCREAS

Type of affiliation / financial interest during the past 3 years:

☑ I have no potential conflict of interests to report.

☐ I have the following potential conflict(s) of interest to report:

Receipt of grants/research supports:	
Receipt of advisory, honoraria or consultation fees:	
Participation in a company sponsored speaker's bureau:	
Stock shareholder:	
Spouse/partner:	
Other support (please specify):	

Name: Jens Brøndum Frøkjær

Signature:

Date: 29-JAN-2025

DISCLOSURE STATEMENT

EPC POSITION STATEMENT ON FATTY PANCREAS

Type of affiliation / financial interest during the past 3 years:

☑ I have no potential conflict of interests to report.

☐ I have the following potential conflict(s) of interest to report:

Receipt of grants/research supports:	
Receipt of advisory, honoraria or consultation fees:	
Participation in a company sponsored speaker's bureau:	
Stock shareholder:	
Spouse/partner:	
Other support (please specify):	

Name: Pramod Garg

Signature: [illegible]

Date: 28-01-2025

E·P·C
European Pancreatic Club
Founded 1965

DISCLOSURE STATEMENT

EPC POSITION STATEMENT ON FATTY PANCREAS

Type of affiliation / financial interest during the past 3 years:

☒ I have no potential conflict of interests to report.

☐ I have the following potential conflict(s) of interest to report:

Receipt of grants/research supports:	
Receipt of advisory, honoraria or consultation fees:	
Participation in a company sponsored speaker's bureau:	
Stock shareholder:	
Spouse/partner:	
Other support (please specify):	

Name: Natalya Gubergrits

Signature: [signature]

Date: 18 Feb 2025

DISCLOSURE STATEMENT

EPC POSITION STATEMENT ON FATTY PANCREAS

Type of affiliation / financial interest during the past 3 years:

☐ I have no potential conflict of interests to report.

[X] I have the following potential conflict(s) of interest to report:

Receipt of grants/research supports:	NA
Receipt of advisory, honoraria or consultation fees:	Consultant to Boston Scientific
Participation in a company sponsored speaker's bureau:	NA
Stock shareholder:	NA
Spouse/partner:	NA
Other support (please specify):	NA

Name: Roberto Valente

Signature: R.V.

Date: 15-02-2025

E·P·C

DISCLOSURE STATEMENT

EPC POSITION STATEMENT ON FATTY PANCREAS

Type of affiliation / financial interest during the past 3 years:

☐ I have no potential conflict of interests to report.

☒ I have the following potential conflict(s) of interest to report:

Receipt of grants/research supports:	HUMAiX AWARD 2024
Receipt of advisory, honoraria or consultation fees:	
Participation in a company sponsored speaker's bureau:	
Stock shareholder:	
Spouse/partner:	
Other support (please specify):	

Name: Felicia Gerst

Signature: [illegible]

Date: 02.04.2025

DISCLOSURE STATEMENT

EPC POSITION STATEMENT ON FATTY PANCREAS

Type of affiliation / financial interest during the past 3 years:

☑ I have no potential conflict of interests to report.

☐ I have the following potential conflict(s) of interest to report:

Receipt of grants/research supports:	
Receipt of advisory, honoraria or consultation fees:	
Participation in a company sponsored speaker's bureau:	
Stock shareholder:	
Spouse/partner:	
Other support (please specify):	

Name: Antanas Gulbinas

Signature:

Date: 29 - JAN - 2025

E•P•C
European Pancreatic Club
Founded 1965

DISCLOSURE STATEMENT

EPC POSITION STATEMENT ON FATTY PANCREAS

Type of affiliation / financial interest during the past 3 years:

☒ I have no potential conflict of interests to report.

☐ I have the following potential conflict(s) of interest to report:

Receipt of grants/research supports:	
Receipt of advisory, honoraria or consultation fees:	
Participation in a company sponsored speaker's bureau:	
Stock shareholder:	
Spouse/partner:	
Other support (please specify):	

Name: Ibrahim Halil Gurcinar

Signature:

Date: 28.01.2025

E·P·C
European Pancreatic Club
Founded 1965

DISCLOSURE STATEMENT

EPC POSITION STATEMENT ON FATTY PANCREAS

Type of affiliation / financial interest during the past 3 years:

☒ I have no potential conflict of interests to report.

☐ I have the following potential conflict(s) of interest to report:

Receipt of grants/research supports:	
Receipt of advisory, honoraria or consultation fees:	
Participation in a company sponsored speaker's bureau:	
Stock shareholder:	
Spouse/partner:	
Other support (please specify):	

Name: PETER HEGYI

Signature: [signature]

Date: 29/1/2025

E·P·C
European Pancreatic Club
Founded 1965

DISCLOSURE STATEMENT

EPC POSITION STATEMENT ON FATTY PANCREAS

Type of affiliation / financial interest during the past 3 years:

☐ I have no potential conflict of interests to report.

☒ I have the following potential conflict(s) of interest to report:

Receipt of grants/research supports:	/
Receipt of advisory, honoraria or consultation fees:	Boehringer Ingelheim, Chiesi/Amryt
Participation in a company sponsored speaker's bureau:	Lilly, Boehringer Ingelheim, Novo Nordisk Novartis, Sanofi, Astra Zeneca, Chiesi
Stock shareholder:	/
Spouse/partner:	/
Other support (please specify):	/

Name: Prof. Dr. M. Heni

Signature: [signature]

Date: 30.01.2025

E·P·C
European Pancreatic Club
Founded 1965

DISCLOSURE STATEMENT

EPC POSITION STATEMENT ON FATTY PANCREAS

Type of affiliation / financial interest during the past 3 years:

☑ I have no potential conflict of interests to report.

☐ I have the following potential conflict(s) of interest to report:

Receipt of grants/research supports:	None
Receipt of advisory, honoraria or consultation fees:	None
Participation in a company sponsored speaker's bureau:	None
Stock shareholder:	None
Spouse/partner:	None
Other support (please specify):	None

Name: Ilkey S. Idilman

Signature:

Date: 07 Feb 2025

DISCLOSURE STATEMENT

EPC POSITION STATEMENT ON FATTY PANCREAS

Type of affiliation / financial interest during the past 3 years:

☐ I have no potential conflict of interests to report.

☒ I have the following potential conflict(s) of interest to report:

Receipt of grants/research supports:	
Receipt of advisory, honoraria or consultation fees:	Fujifilm, Boston Scientific, Viatris, Mediglobe, MiTech
Participation in a company sponsored speaker's bureau:	
Stock shareholder:	
Spouse/partner:	
Other support (please specify):	

Name: Julio Iglesias-Garcia

Signature:

Date: 10-05-2025

DISCLOSURE STATEMENT

EPC POSITION STATEMENT ON FATTY PANCREAS

Type of affiliation / financial interest during the past 3 years:

☑ I have no potential conflict of interests to report.

☐ I have the following potential conflict(s) of interest to report:

Receipt of grants/research supports:	
Receipt of advisory, honoraria or consultation fees:	
Participation in a company sponsored speaker's bureau:	
Stock shareholder:	
Spouse/partner:	
Other support (please specify):	

Name: Patrick Jacquemin

Signature: PJacquemin

Date: 28/01/25

European Pancreatic Club (EPC) | Wickenburggasse 1 | 1080 Vienna, Austria
General Secretary: Dr. Gabriele Capurso | Treasurer: Professor Heiko Witt | President 2025: Professor Irene Esposito
Email: info@europeanpancreaticclub.org | www.europeanpancreaticclub.org | Registered Austrian Association: ZVR 160 370 6974
Erste Bank: IBAN AT11 2011 1842 1568 3400 | BIC GIBAATWWXXX | Account name: European Pancreatic Club

DISCLOSURE STATEMENT

EPC POSITION STATEMENT ON FATTY PANCREAS

Type of affiliation / financial interest during the past 3 years:

☒ I have no potential conflict of interests to report.

☐ I have the following potential conflict(s) of interest to report:

Receipt of grants/research supports:	
Receipt of advisory, honoraria or consultation fees:	
Participation in a company sponsored speaker's bureau:	
Stock shareholder:	
Spouse/partner:	
Other support (please specify):	

Name: Prof Eduard Jonas

Signature:

Date: 2025-05-09

E•P•C
European Pancreatic Club
Founded 1965

DISCLOSURE STATEMENT

EPC POSITION STATEMENT ON FATTY PANCREAS

Type of affiliation / financial interest during the past 3 years:

☑ I have no potential conflict of interests to report.

☐ I have the following potential conflict(s) of interest to report:

Receipt of grants/research supports:	
Receipt of advisory, honoraria or consultation fees:	
Participation in a company sponsored speaker's bureau:	
Stock shareholder:	
Spouse/partner:	
Other support (please specify):	

Name: Jong Jin Hyun

Signature: [signature]

Date: Feb. 06, 2025

DISCLOSURE STATEMENT

EPC POSITION STATEMENT ON FATTY PANCREAS

Type of affiliation / financial interest during the past 3 years:

☐ I have no potential conflict of interests to report.

X I have the following potential conflict(s) of interest to report:

Receipt of grants/research supports:	
Receipt of advisory, honoraria or consultation fees:	Bayer, GE, Canon
Participation in a company sponsored speaker's bureau:	
Stock shareholder:	
Spouse/partner:	
Other support (please specify):	

Name: Musturay Karçaaltıncaba

Signature:

Date: 01.04.2025

E·P·C
European Pancreatic Club
Founded 1965

DISCLOSURE STATEMENT

EPC POSITION STATEMENT ON FATTY PANCREAS

Type of affiliation / financial interest during the past 3 years:

√☐ I have no potential conflict of interests to report.

☑ I have the following potential conflict(s) of interest to report:

Receipt of grants/research supports:	
Receipt of advisory, honoraria or consultation fees:	
Participation in a company sponsored speaker's bureau:	
Stock shareholder:	
Spouse/partner:	
Other support (please specify):	

Name: Mariia Kiriukova

Signature: [signature]

Date: 05.02.2025

European Pancreatic Club (EPC) | Wickenburggasse 1 | 1080 Vienna, Austria
General Secretary: Dr. Gabriele Capurso | Treasurer: Professor Heiko Witt | President 2025: Professor Irene Esposito
Email: info@europeanpancreaticclub.org | www.europeanpancreaticclub.org | Registered Austrian Association: ZVR 160 370 6974
Erste Bank: IBAN AT11 2011 1842 1568 3400 | BIC GIBAATWWXXX | Account name: European Pancreatic Club

DISCLOSURE STATEMENT

EPC POSITION STATEMENT ON FATTY PANCREAS

Type of affiliation / financial interest during the past 3 years:

☑ I have no potential conflict of interests to report.

☐ I have the following potential conflict(s) of interest to report:

Receipt of grants/research supports:	
Receipt of advisory, honoraria or consultation fees:	
Participation in a company sponsored speaker's bureau:	
Stock shareholder:	
Spouse/partner:	
Other support (please specify):	

Name: Masayuki Kitano

Signature: Masayuki Kitano

Date: Jan 29, 2025

DISCLOSURE STATEMENT

EPC POSITION STATEMENT ON FATTY PANCREAS

Type of affiliation / financial interest during the past 3 years: ☐ I have no potential conflict of interests to report.

☐⃠ I have the following potential conflict(s) of interest to report:

Receipt of grants/research supports:	Astra, Siemens, Nordic Bioscience, Echosense
Receipt of advisory, honoraria or consultation fees:	Novo Nordisk, GSK, Boehringer Ingelheim
Participation in a company sponsored speaker's bureau:	Novo Nordisk, Norgine
Stock shareholder:	Evido
Spouse/partner:	
Other support (please specify):	

Name: Aleksander Krag

Signature: Aleksander Krag Digitalt signeret af Aleksander Krag

Date: Dato: 2025.01.31 09:54:34 +01'00'

DISCLOSURE STATEMENT

EPC POSITION STATEMENT ON FATTY PANCREAS

Type of affiliation / financial interest during the past 3 years:

X I have no potential conflict of interests to report.

☐ I have the following potential conflict(s) of interest to report:

Receipt of grants/research supports:	
Receipt of advisory, honoraria or consultation fees:	
Participation in a company sponsored speaker's bureau:	
Stock shareholder:	
Spouse/partner:	
Other support (please specify):	

Name: Johanna Laukkarinen

Signature: Johanna Laukkarinen

Date: February 5th, 2025

Document ID: 0ad7d40e-056d-46c3-918b-8cab73a7ba2c

DISCLOSURE STATEMENT

EPC POSITION STATEMENT ON FATTY PANCREAS

Type of affiliation / financial interest during the past 3 years:

☒ I have no potential conflict of interests to report.

☐ I have the following potential conflict(s) of interest to report:

Receipt of grants/research supports:	
Receipt of advisory, honoraria or consultation fees:	
Participation in a company sponsored speaker's bureau:	
Stock shareholder:	
Spouse/partner:	
Other support (please specify):	

Name: Monika Lipp

Signature: [signature]

Date: 15.02.2025

DISCLOSURE STATEMENT

EPC POSITION STATEMENT ON FATTY PANCREAS

Type of affiliation / financial interest during the past 3 years:

☐ I have no potential conflict of interests to report.

X I have the following potential conflict(s) of interest to report:

Receipt of grants/research supports:	n/a
Receipt of advisory, honoraria or consultation fees:	Abbott, Falk, Nordmark, Viatris
Participation in a company sponsored speaker's bureau:	n/a
Stock shareholder:	n/a
Spouse/partner:	nothing
Other support (please specify):	n/a

Name: J.-Matthias LÖHR

Signature:

Date: 14-MAR-2025

European Pancreatic Club (EPC) | Wickenburggasse 1 | 1080 Vienna, Austria
General Secretary: Dr. Gabriele Capurso | Treasurer: Professor Heiko Witt | President 2025: Professor Irene Esposito
Email: info@europeanpancreaticclub.org | www.europeanpancreaticclub.org | Registered Austrian Association: ZVR 160 370 6974
Erste Bank: IBAN AT11 2011 1842 1568 3400 | BIC GIBAATWWXXX | Account name: European Pancreatic Club

E·P·C
European Pancreatic Club
Founded 1965

DISCLOSURE STATEMENT

EPC POSITION STATEMENT ON FATTY PANCREAS

Type of affiliation / financial interest during the past 3 years: UNIVERSITY HOSPITAL OLOMOUC
PALACKY UNIVERSITY OLOMOUC

☒ I have no potential conflict of interests to report.

☐ I have the following potential conflict(s) of interest to report:

Receipt of grants/research supports:	MINISTRY OF HEALTH, CZECH REPUBLIC
Receipt of advisory, honoraria or consultation fees:	/
Participation in a company sponsored speaker's bureau:	/
Stock shareholder:	/
Spouse/partner:	/
Other support (please specify):	/

Name: MARTIN LOVEČEK

Signature: [signature]

Date: 29.1.2025

Skenováno pomocí CamScanner

DISCLOSURE STATEMENT

EPC POSITION STATEMENT ON FATTY PANCREAS

Type of affiliation / financial interest during the past 3 years:

☐ I have no potential conflict of interests to report.

☒ I have the following potential conflict(s) of interest to report:

Receipt of grants/research supports:	Italian ministry of university and research - Project on ampullary and periampullary cancers (PRIN_2022)
Receipt of advisory, honoraria or consultation fees:	Astellas
Participation in a company sponsored speaker's bureau:	Bayer / MSD
Stock shareholder:	X
Spouse/partner:	X
Other support (please specify):	X

Name: CLAUDIO LUCHINI

Signature: [signature]

Date: 02/18/'25

DISCLOSURE STATEMENT

EPC POSITION STATEMENT ON FATTY PANCREAS

Type of affiliation / financial interest during the past 3 years:

☒ I have no potential conflict of interests to report.

☐ I have the following potential conflict(s) of interest to report:

Receipt of grants/research supports:	
Receipt of advisory, honoraria or consultation fees:	
Participation in a company sponsored speaker's bureau:	
Stock shareholder:	
Spouse/partner:	
Other support (please specify):	

Name: Patrick Maisonneuve

Signature:

Date: 25/02/2025

European Pancreatic Club
Founded 1965

DISCLOSURE STATEMENT

EPC POSITION STATEMENT ON FATTY PANCREAS

Type of affiliation / financial interest during the past 3 years:

☒ I have no potential conflict of interests to report.

☐ I have the following potential conflict(s) of interest to report:

Receipt of grants/research supports:	
Receipt of advisory, honoraria or consultation fees:	
Participation in a company sponsored speaker's bureau:	
Stock shareholder:	
Spouse/partner:	
Other support (please specify):	

Name: Giovanni Marchegiani

Signature:

Date: 29/1/2025

DISCLOSURE STATEMENT

EPC POSITION STATEMENT ON FATTY PANCREAS

Type of affiliation / financial interest during the past 3 years:

☐ I have no potential conflict of interests to report.

☒ I have the following potential conflict(s) of interest to report:

Receipt of grants/research supports:	no
Receipt of advisory, honoraria or consultation fees:	yes, Amount: about 5000.-€ / year
Participation in a company sponsored speaker's bureau:	no
Stock shareholder:	no
Spouse/partner:	no
Other support (please specify):	no

Name: Marc E. Martignoni

Signature: Marc Martignoni

Date: 2025-04-25

DISCLOSURE STATEMENT

EPC POSITION STATEMENT ON FATTY PANCREAS

Type of affiliation / financial interest during the past 3 years:

☐x I have no potential conflict of interests to report.

☐ I have the following potential conflict(s) of interest to report:

Receipt of grants/research supports:	
Receipt of advisory, honoraria or consultation fees:	
Participation in a company sponsored speaker's bureau:	
Stock shareholder:	
Spouse/partner:	
Other support (please specify):	

Name: ETNA MASIP SIMO

Signature: ETNA| MASIP| SIMO Firmado digitalmente por ETNA|MASIP|SIMO Fecha: 2025.02.04 20:02:04 +01'00'

Date: 04/02/2025

DISCLOSURE STATEMENT

EPC POSITION STATEMENT ON FATTY PANCREAS

Type of affiliation / financial interest during the past 3 years:

☑ I have no potential conflict of interests to report.

☐ I have the following potential conflict(s) of interest to report:

Receipt of grants/research supports:	
Receipt of advisory, honoraria or consultation fees:	
Participation in a company sponsored speaker's bureau:	
Stock shareholder:	
Spouse/partner:	
Other support (please specify):	

Name: Ryotaro Matsumoto

Signature: Ryotaro Matsumoto

Date: 2.5.2025

E·P·C
European Pancreatic Club
Founded 1965

DISCLOSURE STATEMENT

EPC POSITION STATEMENT ON FATTY PANCREAS

Type of affiliation / financial interest during the past 3 years:

☑ I have no potential conflict of interests to report.

☐ I have the following potential conflict(s) of interest to report:

Receipt of grants/research supports:	
Receipt of advisory, honoraria or consultation fees:	
Participation in a company sponsored speaker's bureau:	
Stock shareholder:	
Spouse/partner:	
Other support (please specify):	

CHA 의과학대학교
분당차병원

Name: Min Je Sung

Signature: [signature]

Date: March. 20th. 2025

2025-03-20 22:36:42 | A2****5

DISCLOSURE STATEMENT

EPC POSITION STATEMENT ON FATTY PANCREAS

Type of affiliation / financial interest during the past 3 years:

☒ I have no potential conflict of interests to report.

☐ I have the following potential conflict(s) of interest to report:

Receipt of grants/research supports:	
Receipt of advisory, honoraria or consultation fees:	
Participation in a company sponsored speaker's bureau:	
Stock shareholder:	
Spouse/partner:	
Other support (please specify):	

Name: ANDERS MOLVEN

Signature: Anders Molven

Date: Bergen, 28.01.2025

European Pancreatic Club (EPC) | Wickenburggasse 1 | 1080 Vienna, Austria
General Secretary: Dr. Gabriele Capurso | Treasurer: Professor Heiko Witt | President 2025: Professor Irene Esposito
Email: info@europeanpancreaticclub.org | www.europeanpancreaticclub.org | Registered Austrian Association: ZVR 160 370 6974
Erste Bank: IBAN AT11 2011 1842 1568 3400 | BIC GIBAATWWXXX | Account name: European Pancreatic Club

DISCLOSURE STATEMENT

EPC POSITION STATEMENT ON FATTY PANCREAS

Type of affiliation / financial interest during the past 3 years:

☑ I have no potential conflict of interests to report.

☐ I have the following potential conflict(s) of interest to report:

Receipt of grants/research supports:	No
Receipt of advisory, honoraria or consultation fees:	No
Participation in a company sponsored speaker's bureau:	No
Stock shareholder:	No
Spouse/partner:	No
Other support (please specify):	

Name: Tetiana Mozhyna

Signature: [signature]

Date: 19/02/2025

E•P•C
European Pancreatic Club
Founded 1965

DISCLOSURE STATEMENT

EPC POSITION STATEMENT ON FATTY PANCREAS

Type of affiliation / financial interest during the past 3 years:

☐ I have no potential conflict of interests to report.

☒ I have the following potential conflict(s) of interest to report:

Receipt of grants/research supports:	DFG, German Cancer Aid, Sander
Receipt of advisory, honoraria or consultation fees:	Amgen, Boston, Falk, Takeda
Participation in a company sponsored speaker's bureau:	/
Stock shareholder:	/
Spouse/partner:	/
Other support (please specify):	/

Name: Albrecht Neesse

Signature: [signature]

Date: 29/1/25

UNIVERSITÄTSMEDIZIN GÖTTINGEN
GEORG-AUGUST-UNIVERSITÄT
Prof. Dr. Dr. Albrecht Neeße, Oberarzt
Klinik für Gastroenterologie, gastrointestinale Onkologie und Endokrinologie
Robert-Koch-Str. 40, 37075 Göttingen
Tel. 0551/39-64292, Fax 0551/39-67074

DISCLOSURE STATEMENT

EPC POSITION STATEMENT ON FATTY PANCREAS

Type of affiliation / financial interest during the past 3 years:

☑ I have no potential conflict of interests to report.

☐ I have the following potential conflict(s) of interest to report:

Receipt of grants/research supports:	
Receipt of advisory, honoraria or consultation fees:	
Participation in a company sponsored speaker's bureau:	
Stock shareholder:	
Spouse/partner:	
Other support (please specify):	

Name: MUDr. Lenka Nosáková, PhD.

Signature:

Date: 1.2.2025

E·P·C
European Pancreatic Club
Founded 1965

DISCLOSURE STATEMENT

EPC POSITION STATEMENT ON FATTY PANCREAS

Type of affiliation / financial interest during the past 3 years:

☑ I have no potential conflict of interests to report.

☐ I have the following potential conflict(s) of interest to report:

Receipt of grants/research supports:	
Receipt of advisory, honoraria or consultation fees:	
Participation in a company sponsored speaker's bureau:	
Stock shareholder:	
Spouse/partner:	
Other support (please specify):	

Name: Verena Obmann

Signature: [illegible]

Date: 3.3.2025

DISCLOSURE STATEMENT

EPC POSITION STATEMENT ON FATTY PANCREAS

Type of affiliation / financial interest during the past 3 years:

☒ I have no potential conflict of interests to report.

☐ I have the following potential conflict(s) of interest to report:

Receipt of grants/research supports:	no
Receipt of advisory, honoraria or consultation fees:	no
Participation in a company sponsored speaker's bureau:	no
Stock shareholder:	no
Spouse/partner:	no
Other support (please specify):	

Name: Johann Ockenga

Signature: Johann Ockenga

Date: 04.02.2025

E·P·C
European Pancreatic Club
Founded 1965

DISCLOSURE STATEMENT

EPC POSITION STATEMENT ON FATTY PANCREAS

Type of affiliation / financial interest during the past 3 years:

☒ I have no potential conflict of interests to report.

☐ I have the following potential conflict(s) of interest to report:

Receipt of grants/research supports:	
Receipt of advisory, honoraria or consultation fees:	
Participation in a company sponsored speaker's bureau:	
Stock shareholder:	
Spouse/partner:	
Other support (please specify):	

Name: DANIEL ÖHLUND

Signature:

Date: 25-FEB-2025

DISCLOSURE STATEMENT

EPC POSITION STATEMENT ON FATTY PANCREAS

Type of affiliation / financial interest during the past 3 years:

☒ I have no potential conflict of interests to report.

☐ I have the following potential conflict(s) of interest to report:

Receipt of grants/research supports:	
Receipt of advisory, honoraria or consultation fees:	
Participation in a company sponsored speaker's bureau:	
Stock shareholder:	
Spouse/partner:	
Other support (please specify):	

Name: Kasper Overbeek

Signature: [signature]

Date: 30-01-2025

DISCLOSURE STATEMENT

EPC POSITION STATEMENT ON FATTY PANCREAS

Type of affiliation / financial interest during the past 3 years:

☒ I have no potential conflict of interests to report.

☐ I have the following potential conflict(s) of interest to report:

Receipt of grants/research supports:	
Receipt of advisory, honoraria or consultation fees:	
Participation in a company sponsored speaker's bureau:	
Stock shareholder:	
Spouse/partner:	
Other support (please specify):	

Name: Mario Pelaez-Luna, MD, MSc

Signature:

Date: February 7th 2025

European Pancreatic Club (EPC) | Wickenburggasse 1 | 1080 Vienna, Austria
General Secretary: Dr. Gabriele Capurso | Treasurer: Professor Heiko Witt | President 2025: Professor Irene Esposito
Email: info@europeanpancreaticclub.org | www.europeanpancreaticclub.org | Registered Austrian Association: ZVR 160 370 6974
Erste Bank: IBAN AT11 2011 1842 1568 3400 | BIC GIBAATWWXXX | Account name: European Pancreatic Club

E•P•C
European Pancreatic Club
Founded 1965

DISCLOSURE STATEMENT

EPC POSITION STATEMENT ON FATTY PANCREAS

Type of affiliation / financial interest during the past 3 years:

☒ I have no potential conflict of interests to report.

☐ I have the following potential conflict(s) of interest to report:

Receipt of grants/research supports:	None
Receipt of advisory, honoraria or consultation fees:	None
Participation in a company sponsored speaker's bureau:	None -
Stock shareholder:	None
Spouse/partner:	None
Other support (please specify):	

Name: SANJAY PANDANABOYANA

Signature: [signature]

Date: 29/1/25

E·P·C
European Pancreatic Club
Founded 1965

DISCLOSURE STATEMENT

EPC POSITION STATEMENT ON FATTY PANCREAS

Type of affiliation / financial interest during the past 3 years:

☒ I have no potential conflict of interests to report.

☐ I have the following potential conflict(s) of interest to report:

Receipt of grants/research supports:	
Receipt of advisory, honoraria or consultation fees:	
Participation in a company sponsored speaker's bureau:	
Stock shareholder:	
Spouse/partner:	
Other support (please specify):	

Name: Nikola Pavić

Signature:

Date: 06.02.2025

European Pancreatic Club (EPC) | Wickenburggasse 1 | 1080 Vienna, Austria
General Secretary: Dr. Gabriele Capurso | Treasurer: Professor Heiko Witt | President 2025: Professor Irene Esposito
Email: info@europeanpancreaticclub.org | www.europeanpancreaticclub.org | Registered Austrian Association: ZVR 160 370 6974
Erste Bank: IBAN AT11 2011 1842 1568 3400 | BIC GIBAATWWXXX | Account name: European Pancreatic Club

E·P·C
European Pancreatic Club
Founded 1965

DISCLOSURE STATEMENT

EPC POSITION STATEMENT ON FATTY PANCREAS

Type of affiliation / financial interest during the past 3 years:

☐ I have no potential conflict of interests to report.

☑ I have the following potential conflict(s) of interest to report:

Receipt of grants/research supports:	Funded for research from NIH, DoD & AbbVie.
Receipt of advisory, honoraria or consultation fees:	No
Participation in a company sponsored speaker's bureau:	No
Stock shareholder:	No
Spouse/partner:	No
Other support (please specify):	No

Name: GEORGIOS PAPACHRISTOU

Signature: [signature]

Date: 2/26/2025

European Pancreatic Club
Founded 1965

DISCLOSURE STATEMENT

EPC POSITION STATEMENT ON FATTY PANCREAS

Type of affiliation / financial interest during the past 3 years:

☐ I have no potential conflict of interests to report.

☑ I have the following potential conflict(s) of interest to report:

Receipt of grants/research supports:	
Receipt of advisory, honoraria or consultation fees:	
Participation in a company sponsored speaker's bureau:	
Stock shareholder:	
Spouse/partner:	
Other support (please specify):	speaker (on pancreatic exocrine insufficiency) fees from Abbott Argentina

Name: Analia Pasqua

Signature:

Date: February, 18th. 2025

DISCLOSURE STATEMENT

EPC POSITION STATEMENT ON FATTY PANCREAS

Type of affiliation / financial interest during the past 3 years:

☐ I have no potential conflict of interests to report.

☒ I have the following potential conflict(s) of interest to report:

Receipt of grants/research supports:	
Receipt of advisory, honoraria or consultation fees:	
Participation in a company sponsored speaker's bureau:	Pentax Medical, Boston Scientific, Olympus
Stock shareholder:	
Spouse/partner:	
Other support (please specify):	

Name: Katarzyna Pawlak

Signature: Pawlak Katarzyna

Date: 26.02.2025

DISCLOSURE STATEMENT

EPC POSITION STATEMENT ON FATTY PANCREAS

Type of affiliation / financial interest during the past 3 years:

☐ I have no potential conflict of interests to report.

☐ I have the following potential conflict(s) of interest to report:

Receipt of grants/research supports:	
Receipt of advisory, honoraria or consultation fees:	
Participation in a company sponsored speaker's bureau:	
Stock shareholder:	
Spouse/partner:	
Other support (please specify):	

Name: Daniele Piovani

Signature: Daniele Piovani

Date: 30/01/2025

DISCLOSURE STATEMENT

EPC POSITION STATEMENT ON FATTY PANCREAS

Type of affiliation / financial interest during the past 3 years:

☐ I have no potential conflict of interests to report.

X I have the following potential conflict(s) of interest to report

Receipt of grants/research supports:	Institut national de lutte contre le cancer ; Ligue contre le cancer
Receipt of advisory, honoraria or consultation fees:	No
Participation in a company sponsored speaker's bureau:	No
Stock shareholder:	No
Spouse/partner:	No
Other support (please specify):	No

Name: Pr REBOURS Vinciane

Signature:

Date: 26 feb 2025

European Pancreatic Club (EPC) | Wickenburggasse 1 | 1080 Vienna, Austria
General Secretary: Dr. Gabriele Capurso | Treasurer: Professor Heiko Witt | President 2025: Professor Irene Esposito
Email: info@europeanpancreaticclub.org | www.europeanpancreaticclub.org | Registered Austrian Association: ZVR 160 370 6974
Erste Bank: IBAN AT11 2011 1842 1568 3400 | BIC GIBAATWWXXX | Account name: European Pancreatic Club

DISCLOSURE STATEMENT

EPC POSITION STATEMENT ON FATTY PANCREAS

Type of affiliation / financial interest during the past 3 years:

☒ I have no potential conflict of interests to report.

☐ I have the following potential conflict(s) of interest to report:

Receipt of grants/research supports:	
Receipt of advisory, honoraria or consultation fees:	
Participation in a company sponsored speaker's bureau:	
Stock shareholder:	
Spouse/partner:	
Other support (please specify):	

Name: Ivonne Regel

Signature:

Date: January 31, 2025

E·P·C
European Pancreatic Club
Founded 1965

DISCLOSURE STATEMENT

EPC POSITION STATEMENT ON FATTY PANCREAS

Type of affiliation / financial interest during the past 3 years:

X I have no potential conflict of interests to report.

☐ I have the following potential conflict(s) of interest to report:

Receipt of grants/research supports:	
Receipt of advisory, honoraria or consultation fees:	
Participation in a company sponsored speaker's bureau:	
Stock shareholder:	
Spouse/partner:	
Other support (please specify):	

Name: Sara Regnér

Signature: [signature]

Date: 12th of February 2025

DISCLOSURE STATEMENT

EPC POSITION STATEMENT ON FATTY PANCREAS

Type of affiliation / financial interest during the past 3 years:

☑ I have no potential conflict of interests to report.

☐ I have the following potential conflict(s) of interest to report:

Receipt of grants/research supports:	
Receipt of advisory, honoraria or consultation fees:	
Participation in a company sponsored speaker's bureau:	
Stock shareholder:	
Spouse/partner:	
Other support (please specify):	

Name: Stuart Robinson

Signature: Stuart Robinson

Date: 29/01/2025

DISCLOSURE STATEMENT

EPC POSITION STATEMENT ON FATTY PANCREAS

Type of affiliation / financial interest during the past 3 years:

☐ I have no potential conflict of interests to report.

☑ I have the following potential conflict(s) of interest to report:

Receipt of grants/research supports:	
Receipt of advisory, honoraria or consultation fees:	Triveni, Amgen, Viatris, Nordmark, Alexion
Participation in a company sponsored speaker's bureau:	
Stock shareholder:	
Spouse/partner:	
Other support (please specify):	

Name: Rosendahl, Jonas

Signature: Rosendahl Jonas

Digital signiert von: Rosendahl Jonas
Name: CN = Rosendahl Jonas email = Jonas.rosendahl@uk-halle.de OU = UKH, KV KLINIKEN, KIM I, Benutzer
Datum: 2025.03.18 17:49:28 +01'00'

Date: 18th March 2025

DISCLOSURE STATEMENT

EPC POSITION STATEMENT ON FATTY PANCREAS

Type of affiliation / financial interest during the past 3 years:

☐ I have no potential conflict of interests to report.

☒ I have the following potential conflict(s) of interest to report:

Receipt of grants/research supports:	Grant from Fondation pour la Recherche Scientifique (FNRS)
Receipt of advisory, honoraria or consultation fees:	
Participation in a company sponsored speaker's bureau:	
Stock shareholder:	
Spouse/partner:	
Other support (please specify):	

Name: Isabelle Scheers

Signature:

Date: 29Jan2025

DISCLOSURE STATEMENT

EPC POSITION STATEMENT ON FATTY PANCREAS

Type of affiliation / financial interest during the past 3 years:

☑ I have no potential conflict of interests to report.

☐ I have the following potential conflict(s) of interest to report:

Receipt of grants/research supports:	
Receipt of advisory, honoraria or consultation fees:	
Participation in a company sponsored speaker's bureau:	
Stock shareholder:	
Spouse/partner:	
Other support (please specify):	

Name: Prof. Dr. Andrada Seicean

Signature:

Date: 21.02.2025

EPC
European Pancreatic Club
Founded 1965

DISCLOSURE STATEMENT

EPC POSITION STATEMENT ON FATTY PANCREAS

Type of affiliation / financial interest during the past 3 years:

☐ I have no potential conflict of interests to report.

☑ I have the following potential conflict(s) of interest to report:

Receipt of grants/research supports:	NIH, DOD, Arrivo Biopharma
Receipt of advisory, honoraria or consultation fees:	—
Participation in a company sponsored speaker's bureau:	—
Stock shareholder:	Arrio Biopharma
Spouse/partner:	—
Other support (please specify):	—

Name: VIJAY P. SINGH

Signature: [signature]

Date: 02/21/2025

DISCLOSURE STATEMENT

EPC POSITION STATEMENT ON FATTY PANCREAS

Type of affiliation / financial interest during the past 3 years:

☐ I have no potential conflict of interests to report.

☒ I have the following potential conflict(s) of interest to report:

Receipt of grants/research supports:	Novo Nordisk
Receipt of advisory, honoraria or consultation fees:	Novo Nordisk
Participation in a company sponsored speaker's bureau:	
Stock shareholder:	
Spouse/partner:	
Other support (please specify):	

Name: Mark M. Smits

Signature:

Date: 3 February 2025

DISCLOSURE STATEMENT

EPC POSITION STATEMENT ON FATTY PANCREAS

Type of affiliation / financial interest during the past 3 years:

☐ I have no potential conflict of interests to report.

☒ I have the following potential conflict(s) of interest to report:

Receipt of grants/research supports:	
Receipt of advisory, honoraria or consultation fees:	Honoraria for a training course in EUS organized by Pentax (February 2025)
Participation in a company sponsored speaker's bureau:	
Stock shareholder:	
Spouse/partner:	
Other support (please specify):	

Name: Matteo Tacelli

Signature: [illegible]

Date: 31/01/2025

DISCLOSURE STATEMENT

EPC POSITION STATEMENT ON FATTY PANCREAS

Type of affiliation / financial interest during the past 3 years:

☐ I have no potential conflict of interests to report.

☒ I have the following potential conflict(s) of interest to report:

Receipt of grants/research supports:	-
Receipt of advisory, honoraria or consultation fees:	Advisory fees from Fast 800.
Participation in a company sponsored speaker's bureau:	Academic lectures sponsored by Lilly, Abbott and Novo Nordisk
Stock shareholder:	
Spouse/partner:	
Other support (please specify):	Author of books

Name: Roy Taylor

Signature: Roy Taylor

Date: 27th Feb 2025

DISCLOSURE STATEMENT

EPC POSITION STATEMENT ON FATTY PANCREAS

Type of affiliation / financial interest during the past 3 years:

☒ I have no potential conflict of interests to report.

☐ I have the following potential conflict(s) of interest to report:

Receipt of grants/research supports:	
Receipt of advisory, honoraria or consultation fees:	
Participation in a company sponsored speaker's bureau:	
Stock shareholder:	
Spouse/partner:	
Other support (please specify):	

Name: BRIGITTA TEUTSCH

Signature:

Date: 30.01.2025

DISCLOSURE STATEMENT

EPC POSITION STATEMENT ON FATTY PANCREAS

Type of affiliation / financial interest during the past 3 years:

☐ I have no potential conflict of interests to report.

☐ I have the following potential conflict(s) of interest to report:

Receipt of grants/research supports:	
Receipt of advisory, honoraria or consultation fees:	
Participation in a company sponsored speaker's bureau:	Reckitt, Gilead, Servier, Astra Zeneca
Stock shareholder:	
Spouse/partner:	
Other support (please specify):	

Name: Udrescu Mihaela

Signature:

Date: 30.01.2025

DISCLOSURE STATEMENT

EPC POSITION STATEMENT ON FATTY PANCREAS

Type of affiliation / financial interest during the past 3 years:

☒ I have no potential conflict of interests to report.

☐ I have the following potential conflict(s) of interest to report:

Receipt of grants/research supports:	
Receipt of advisory, honoraria or consultation fees:	
Participation in a company sponsored speaker's bureau:	
Stock shareholder:	
Spouse/partner:	
Other support (please specify):	

Name: Caroline Verbeke

Signature: Caroline Verbeke

Date: 28.01.2025

DISCLOSURE STATEMENT

EPC POSITION STATEMENT ON FATTY PANCREAS

Type of affiliation / financial interest during the past 3 years:

☐ I have no potential conflict of interests to report.

☑ I have the following potential conflict(s) of interest to report:

Receipt of grants/research supports:	
Receipt of advisory, honoraria or consultation fees:	Abbott, Viatris, Nordmark, Amgen
Participation in a company sponsored speaker's bureau:	
Stock shareholder:	
Spouse/partner:	
Other support (please specify):	

Name: Miroslav Vujasinovic

Signature: Miroslav Vujasinovic

Date:2025-02-05

DISCLOSURE STATEMENT

EPC POSITION STATEMENT ON FATTY PANCREAS

Type of affiliation / financial interest during the past 3 years:

☐ I have no potential conflict of interests to report.

☒ I have the following potential conflict(s) of interest to report:

Receipt of grants/research supports:	
Receipt of advisory, honoraria or consultation fees:	Sanofi, NovoNordisk, Eli Lilly
Participation in a company sponsored speaker's bureau:	Boehringer-Ingelheim, Synlab
Stock shareholder:	
Spouse/partner:	
Other support (please specify):	

Name: Robert Wagner

Signature:

Date: Feb 18, 2025

E·P·C
European Pancreatic Club
Founded 1965

DISCLOSURE STATEMENT

EPC POSITION STATEMENT ON FATTY PANCREAS

Type of affiliation / financial interest during the past 3 years:

☐ I have no potential conflict of interests to report.

☒ I have the following potential conflict(s) of interest to report:

Receipt of grants/research supports:	VERTEX PHARMACEUTICALS
Receipt of advisory, honoraria or consultation fees:	ANAGRAM THERAPEUTICS
Participation in a company sponsored speaker's bureau:	
Stock shareholder:	
Spouse/partner:	
Other support (please specify):	

Name: MICHAEL WILSCHANSKI

Signature: [signature]

Date: FEB 17 2025

Prof. Michael Wilschanski, M.D.
Director, Division of Gastroenterology
Department of Pediatrics
Hadassah University Hospital

European Pancreatic Club
Founded 1965

DISCLOSURE STATEMENT

EPC POSITION STATEMENT ON FATTY PANCREAS

Type of affiliation / financial interest during the past 3 years:

x I have no potential conflict of interests to report.

☐ I have the following potential conflict(s) of interest to report:

Receipt of grants/research supports:	
Receipt of advisory, honoraria or consultation fees:	
Participation in a company sponsored speaker's bureau:	
Stock shareholder:	
Spouse/partner:	
Other support (please specify):	

Name: Prof Dr Heiko Witt

Signature: Prof. Dr. Heiko Witt Digital unterschrieben von Prof. Dr. Heiko Witt Datum: 2025.01.29 09:55:09 +01'00'

Date: 2025-01-29

E·P·C
European Pancreatic Club
Founded 1965

DISCLOSURE STATEMENT

EPC POSITION STATEMENT ON FATTY PANCREAS

Type of affiliation / financial interest during the past 3 years:

☒ I have no potential conflict of interests to report.

☐ I have the following potential conflict(s) of interest to report:

Receipt of grants/research supports:	
Receipt of advisory, honoraria or consultation fees:	
Participation in a company sponsored speaker's bureau:	
Stock shareholder:	
Spouse/partner:	
Other support (please specify):	

Name: GIULIA ZAMBONI

Signature: Giulia Zamboni

Date: 20/2/2025
