# Supplementary material for: International Multidisciplinary Consensus Report on Definitions, Diagnostic Criteria, and Management of Fatty Pancreas: A Joint Statement Endorsed by EPC, APA, EASD, EASL, ESGAR, ESGE, ESP, ESPCG, ESPEN, ESPGHAN, IAP, JPS, KPBA, LAPSG, and UEG
Source: United European Gastroenterol J. 2026 Feb 14;14(1):e70185. doi: 10.1002/ueg2.70185 (PMC12906299; doi:10.1002/ueg2.70185)
Supplement: Supplementary file 2 — Supporting Information S2 [file UEG2-14-e70185-s015.docx]

**Supplement Document 2**

**Meta-analysis**

We performed a systematic literature search initially using PubMed ("pancreatic fat" OR "pancreatic lipomatosis" OR "fatty pancreas" OR "lipomatous pseudohypertrophy" OR "fatty replacement in pancreas" OR "fatty infiltration in pancreas" OR "fatty infiltration of the pancreas" OR "nonalcoholic fatty pancreas disease" OR "nonalcoholic fatty steatopancreatitis" OR "intrapancreatic fat" OR "intra-pancreatic fat" OR "pancreatic lipids" OR "pancreatic steatosis" OR "pancreatic triacylglycerol" OR "pancreatic intracellular lipid" OR "pancreatic triglyceride" OR "intra-pancreatic triglyceride" OR "pancreatic adipocytes" OR "pancreas fat" OR “fat-laden pancreas” OR “fat infiltration in the pancreas” OR “fat deposition in the pancreas” OR “fat accumulation in the pancreas” OR “pancreatic fat accumulation”) AND (epidemiology OR prevalence), with no language or other restrictions, to find papers referring to the epidemiology or prevalence of fatty pancreas.

We also used other databases such as the Cochrane library or Web of Science. Finally, we scrutinized the list of references from key articles and reviews. We did not look for abstracts or unpublished studies. Independent searches were performed by 3 reviewers, and 62 papers were deemed appropriate based on information contained in the abstract (PMIDs 716899, 5964381, 6745910, 7335549, 17666465, 17879305, 19370785, 20225188, 21270204, 21521567, 23671610, 23688357, 24475851, 24492753, 24572250, 24671510, 25491010, 25700304, 25715922, 25740696, 26201937, 26652175, 26954660, 27050733, 27422578, 28285638, 28520813, 28906401, 29040194, 29477252, 29953011, 30483555, 30732577, 30767421, 31593012, 31664423, 31986155, 31992455, 33090101, 33318573, 33485792, 33651254, 34329319, 34731128, 34974993, 35333778, 35848656, 36163226, 36438020, 36919831, 37088949, 37303245, 37598866, 37600695, 37691570, 37862954, 38379586, 38587286, 39128120, 39589806, 39657903, 39816430).

We reviewed the full text of the 62 independent reports: 30 studies were excluded including 4 reviews (PMIDs 28285638, 29040194, 30767421, 39589806), 4 studies conducted in children or adolescent (PMIDs 26201937, 26954660, 39657903, 21270204), 10 studies lacking prevalence information (PMIDs 7335549, 6745910, 23671610, 24475851, 27422578, 31992455, 33318573, 37862954, 25700304 34329319), and 12 studies conducted in peculiar populations (with obesity, diabetes, NAFLD, MASH-MALD, IBD, hypothyroidism, coronavirus or post pancreaticojejunostomy)(PMIDs 17666465, 31664423, 36919831, 37691570, 38379586, 37600695, 35333778, 36163226, 39816430, 33485792, 30483555, 25715922) and 32 were included in the systematic review. Six of the studies did not provided information about prevalence (PMIDs 17879305, 20225188, 24671510, 25740696, 28520813, 31986155). Majority of the remaining 26 studies were conducted between 2009 and 2024 with two older studies based on autopsy material.

We calculated 95% confidence intervals (CIs) of prevalence estimates in single studies using the Clopper–Pearson Exact binomial method. For the meta-analysis, we used random-effects models, with maximum likelihood estimates and the Freeman–Tukey double arcsine transform method to stabilize variances. The percentage of total variation across studies due to heterogeneity was evaluated by the I^2^ measure. We produced forest plots including the study specifics and the overall prevalence estimates. Probability of publication bias was assessed using funnel plots with the Begg’s test. Statistical analyses were performed using the meta package in R. the metaprop function was used to calculate the overall and subgroup proportion of FP in representative samples of the general population.
