# Supplementary material for: International Multidisciplinary Consensus Report on Definitions, Diagnostic Criteria, and Management of Fatty Pancreas: A Joint Statement Endorsed by EPC, APA, EASD, EASL, ESGAR, ESGE, ESP, ESPCG, ESPEN, ESPGHAN, IAP, JPS, KPBA, LAPSG, and UEG
Source: United European Gastroenterol J. 2026 Feb 14;14(1):e70185. doi: 10.1002/ueg2.70185 (PMC12906299; doi:10.1002/ueg2.70185)
Supplement: Supplementary file 3 — Supporting Information S3 [file UEG2-14-e70185-s004.docx]

**Supplement Figure 1**. Begg test of the funnel plot asymmetry.


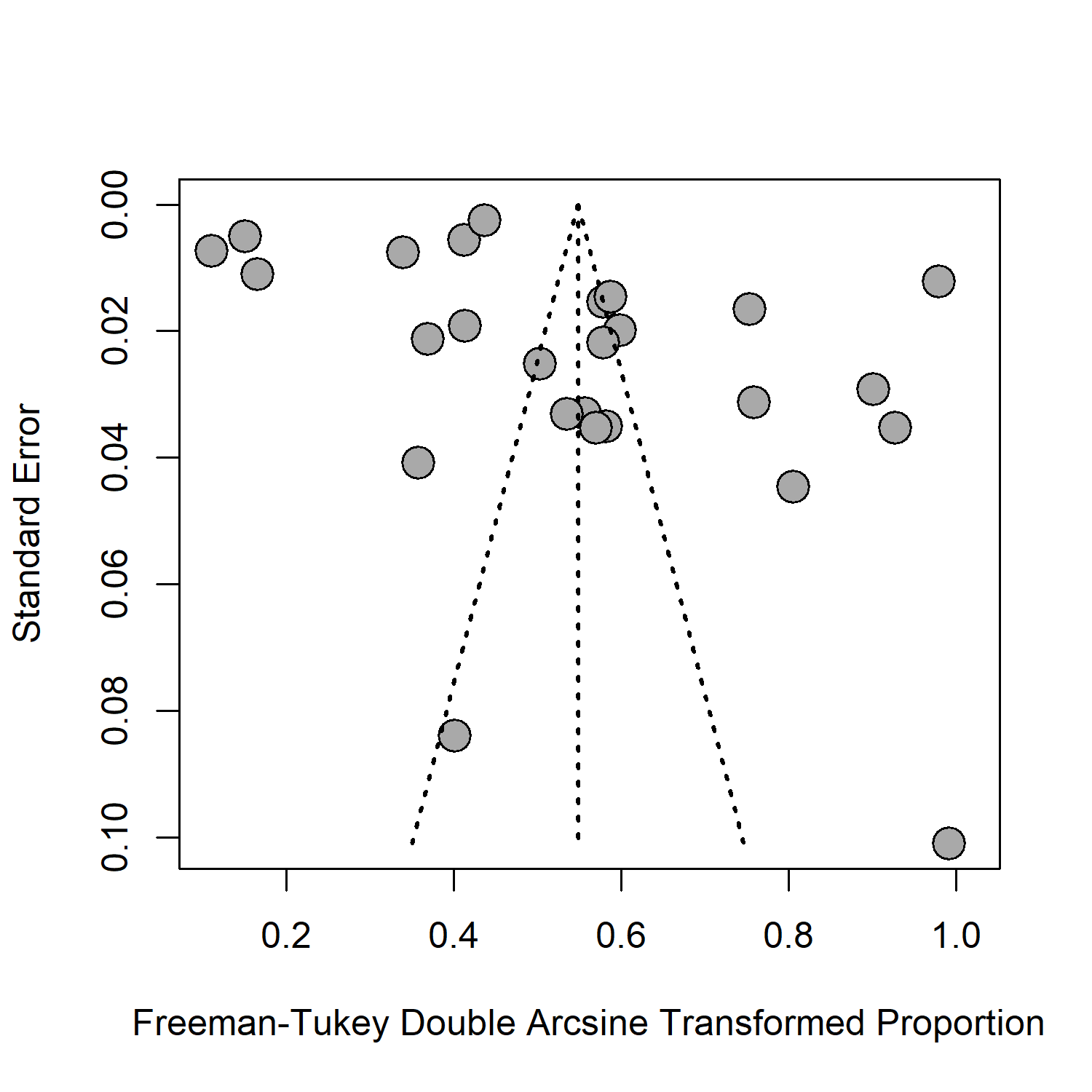


**Supplement Figure 2.** Forest plots of the prevalence of fatty pancreas according to the method of diagnosis used.


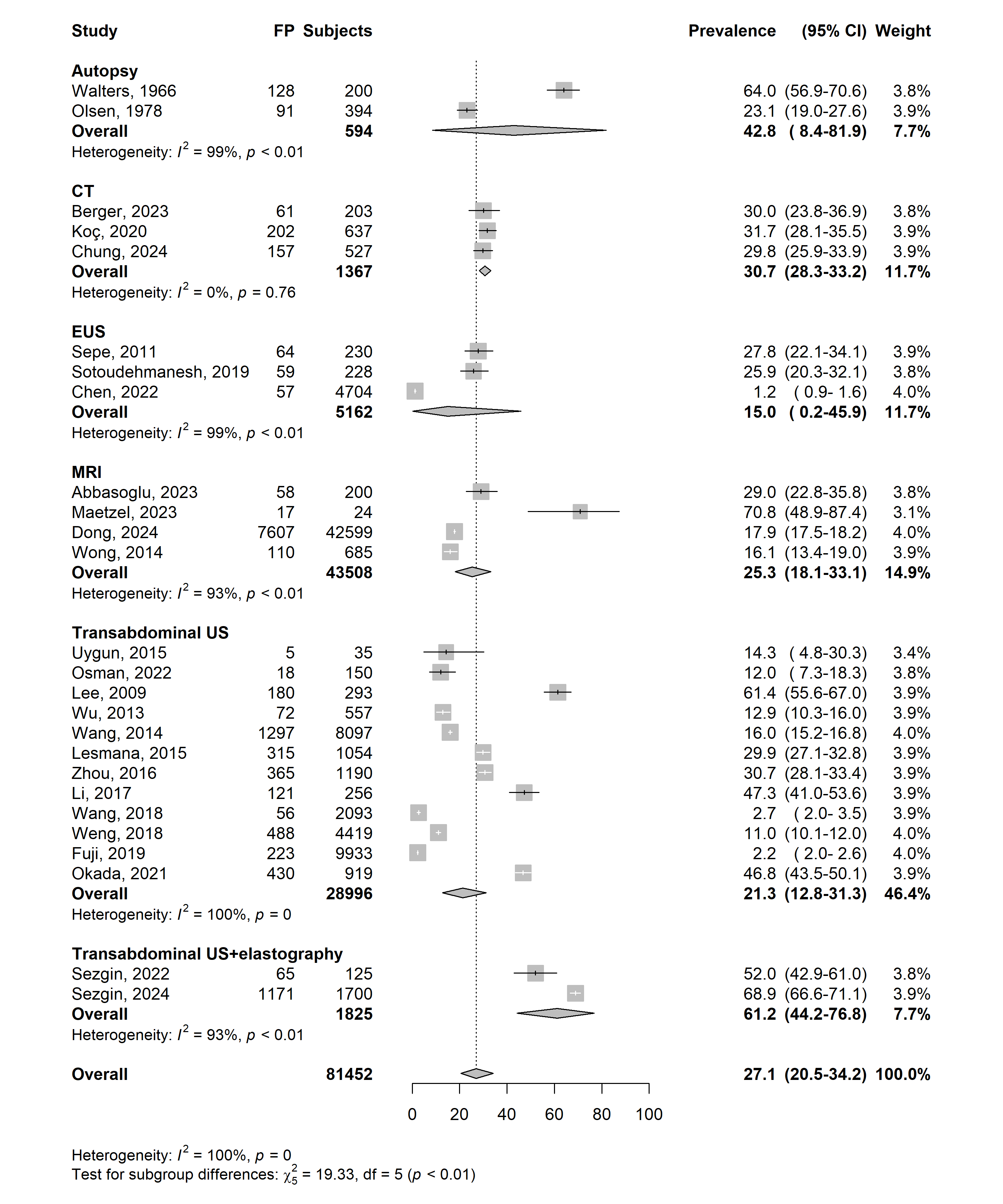


Abbreviations: FP=fatty pancreas; US=ultrasound; CT=computed tomography; EUS=endoscopic ultrasound; MRI=magnetic resonance imaging.

**Supplement Figure 3.** Forest plots of the prevalence of fatty pancreas according to region.


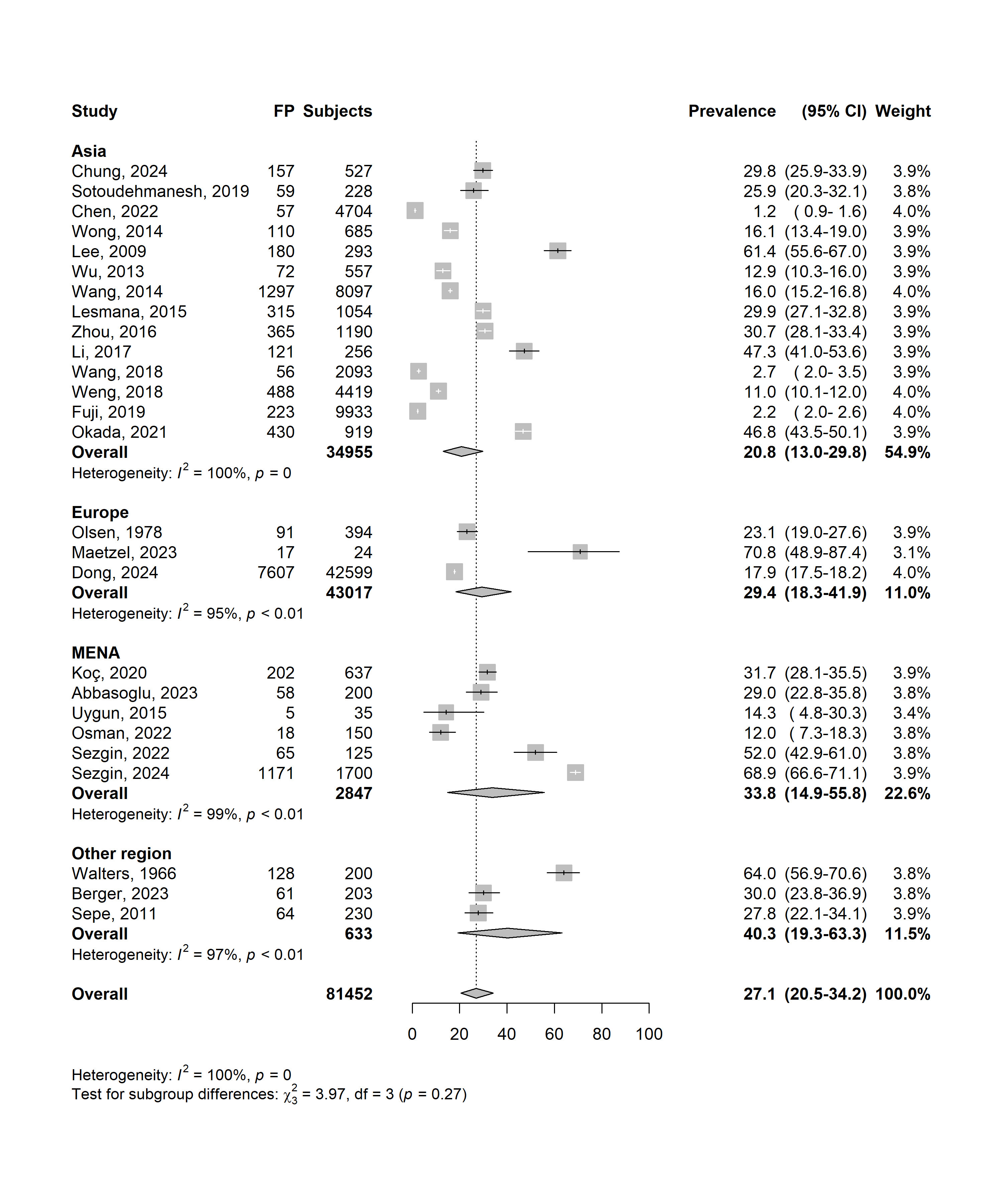


Abbreviations: FP=fatty pancreas; MENA= Middle East and North Africa

Other region includes 1 study from the USA, 1 from Australia and 1 from Chile
