# Supplementary figures and images for: International Multidisciplinary Consensus Report on Definitions, Diagnostic Criteria, and Management of Fatty Pancreas: A Joint Statement Endorsed by EPC, APA, EASD, EASL, ESGAR, ESGE, ESP, ESPCG, ESPEN, ESPGHAN, IAP, JPS, KPBA, LAPSG, and UEG
Source: United European Gastroenterol J. 2026 Feb 14;14(1):e70185. doi: 10.1002/ueg2.70185 (PMC12906299; doi:10.1002/ueg2.70185)

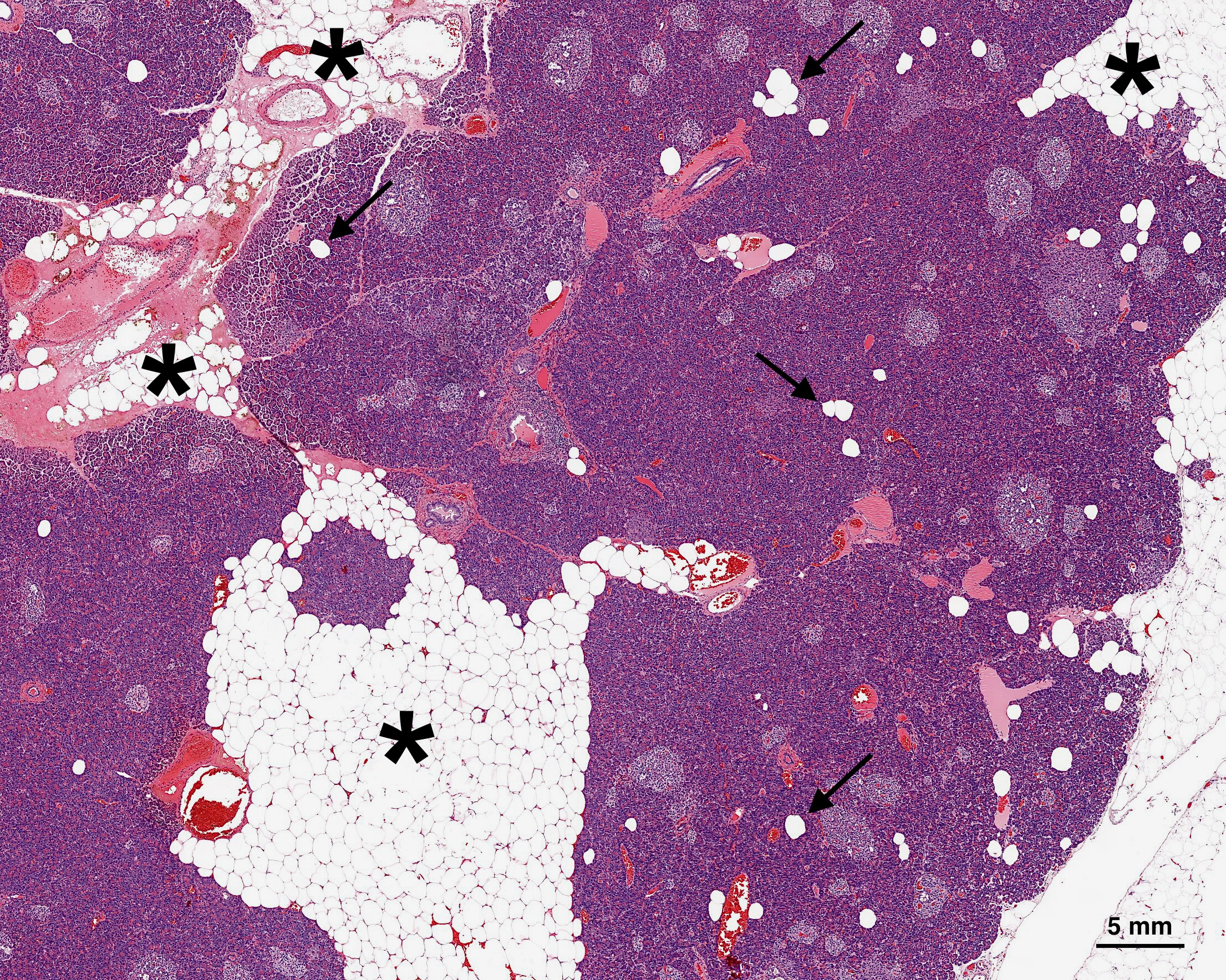

Supplement: Supplementary file 4 — Figure S4: Accumulation of adipocytes both within lobules (intralobular; arrows) and in the interlobular space (extralobular; asterisks). [file UEG2-14-e70185-s020.tiff]

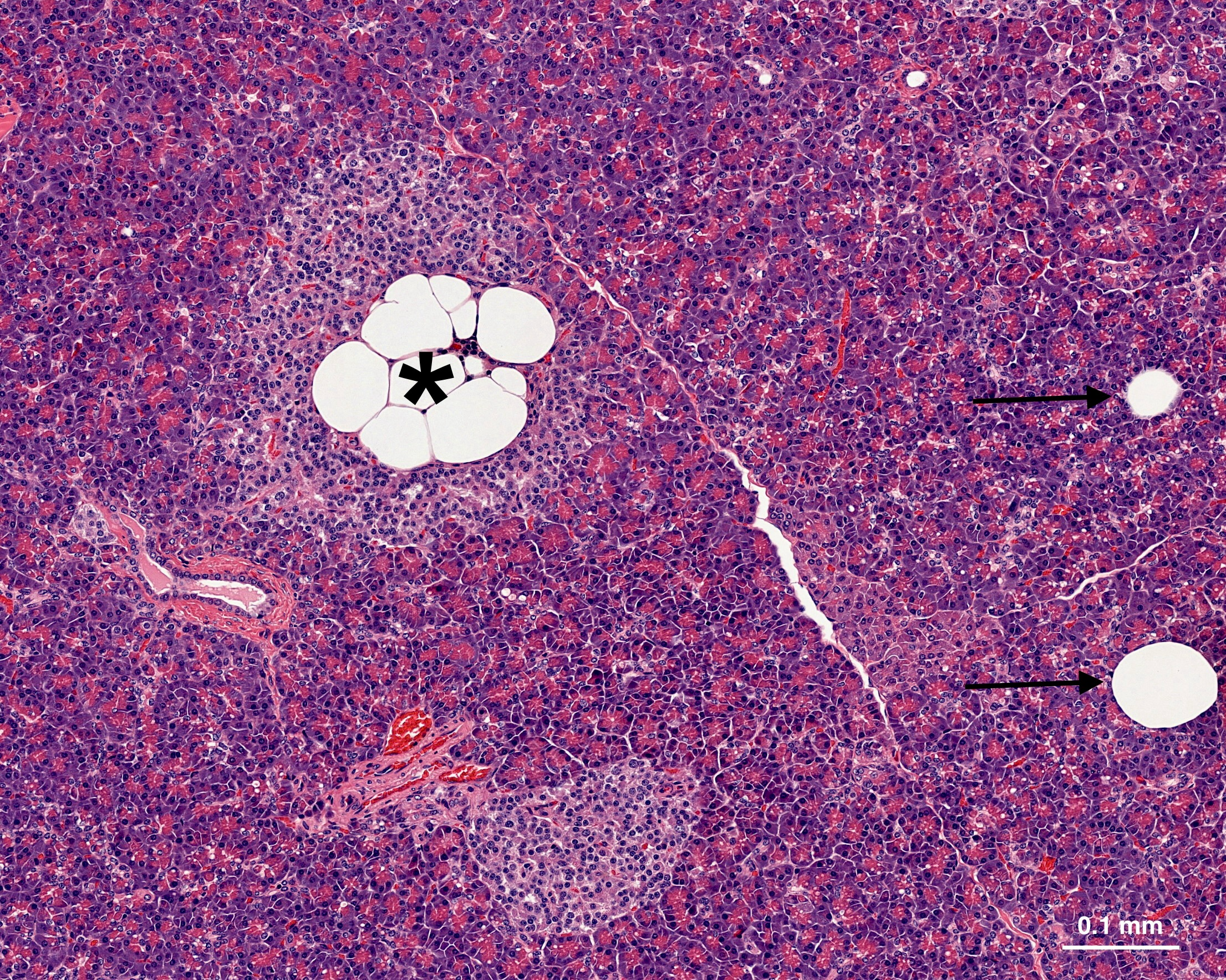

Supplement: Supplementary file 5 — Figure S5: Rare occurrence of adipocytes inside an islet of Langerhans (asterisk). Some intralobular adipocytes (arrows) are also present. [file UEG2-14-e70185-s016.tiff]

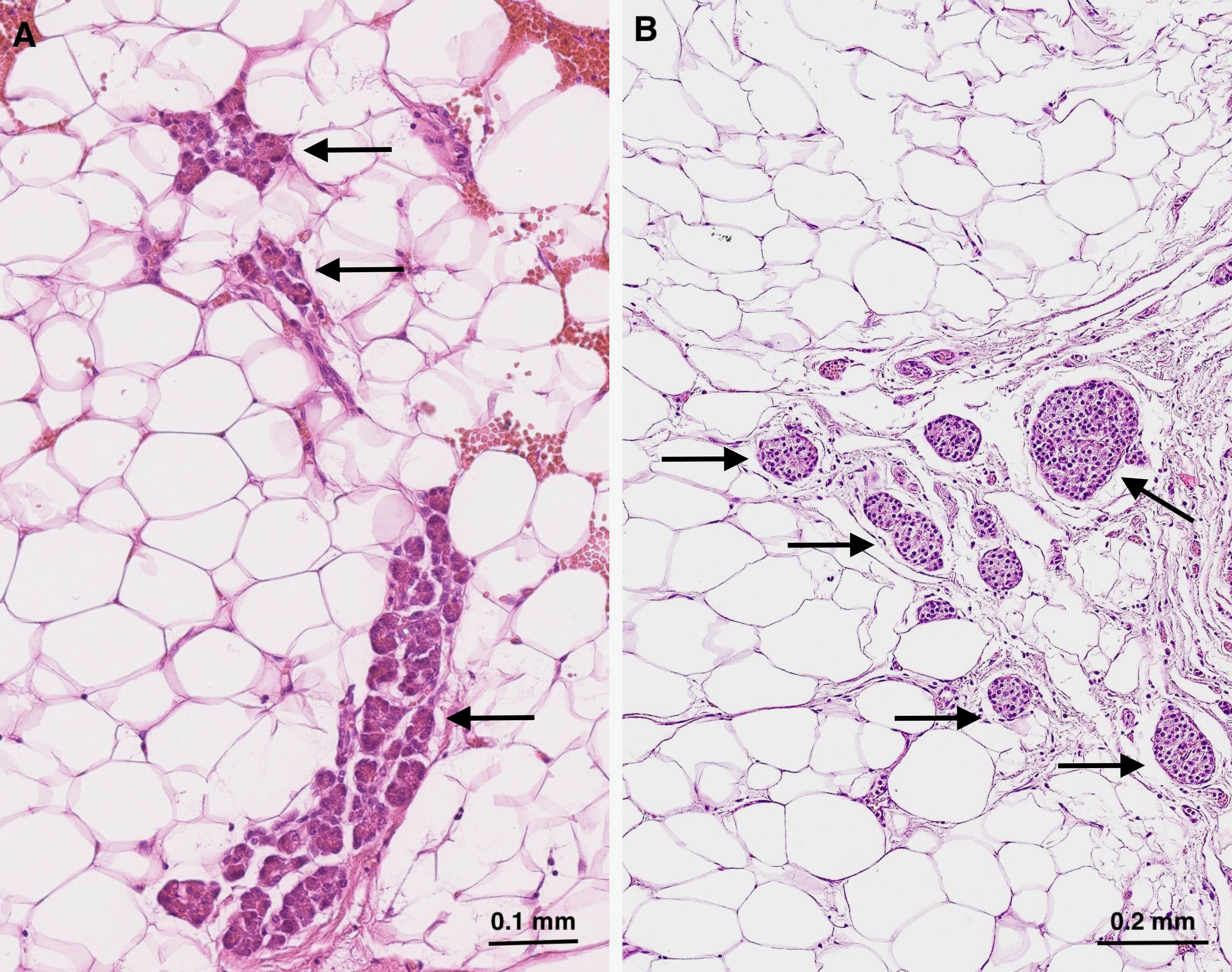

Supplement: Supplementary file 6 — Figure S6: Sparse remnants of acinar parenchyma (a; arrows) and islets of Langerhans (b; arrows) amid sheets of adipocytes in advanced fatty pancreas. [file UEG2-14-e70185-s019.tiff]

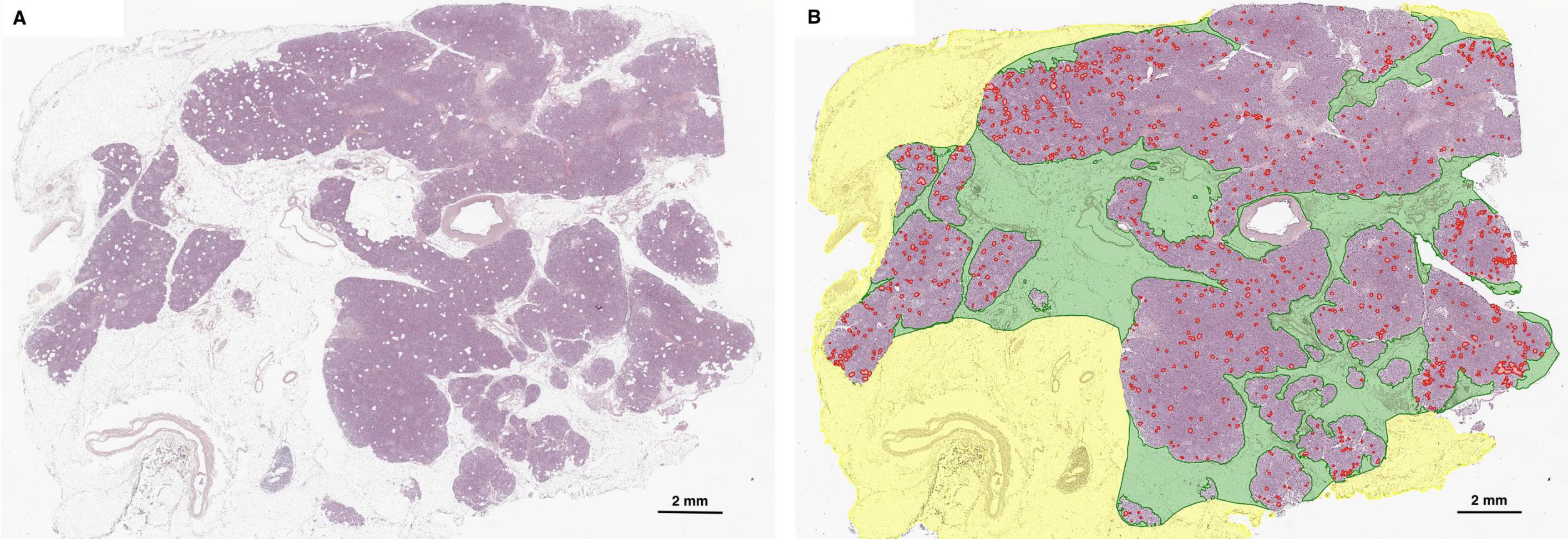

Supplement: Supplementary file 7 — Figure S7: Pancreatic parenchyma with fatty pancreas‐related changes and surrounding peripancreatic fat (a). A line connecting the most peripheral (remnants of) parenchyma demarcates peripancreatic fat (yellow) from extralobular fat (green; intralobular fat: red) (b). [file UEG2-14-e70185-s022.tiff]

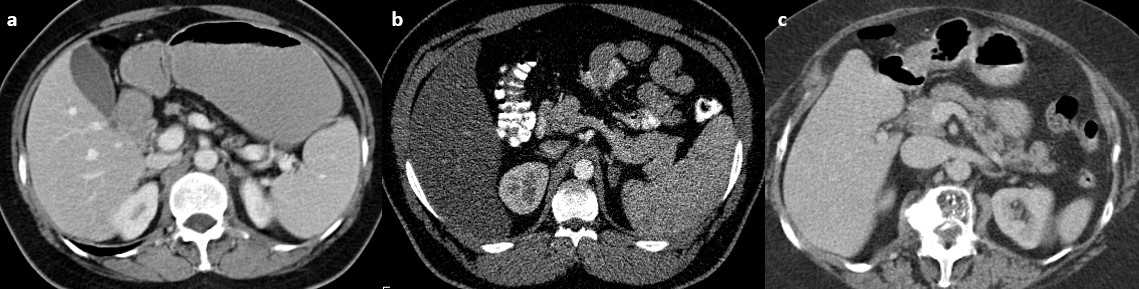

Supplement: Supplementary file 8 — Figure S8: Different types of fatty pancreas in some patients: (a) more pronounced at the head and body, (b) due to focal pancreatitis sequela in the body, (c) patchy nodular fat in the distal pancreas. [file UEG2-14-e70185-s005.tiff]

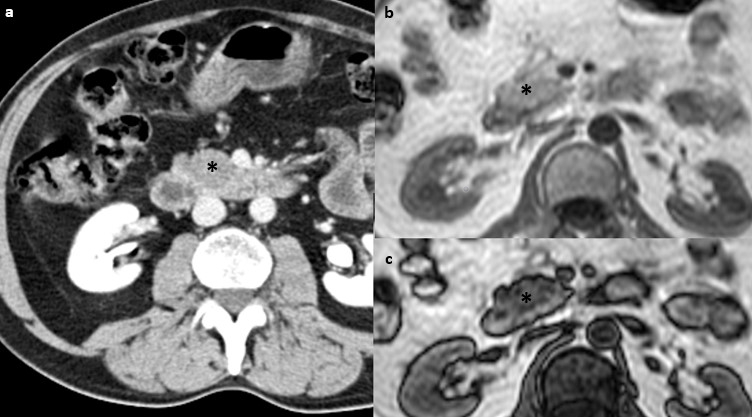

Supplement: Supplementary file 9 — Figure S9: Focal fat in the pancreatic head (*) simulating a mass on computed tomography (a), in‐phase (b), and opposed‐phase (c) images. A signal drop is consistent with the presence of focal fat. [file UEG2-14-e70185-s003.tiff]

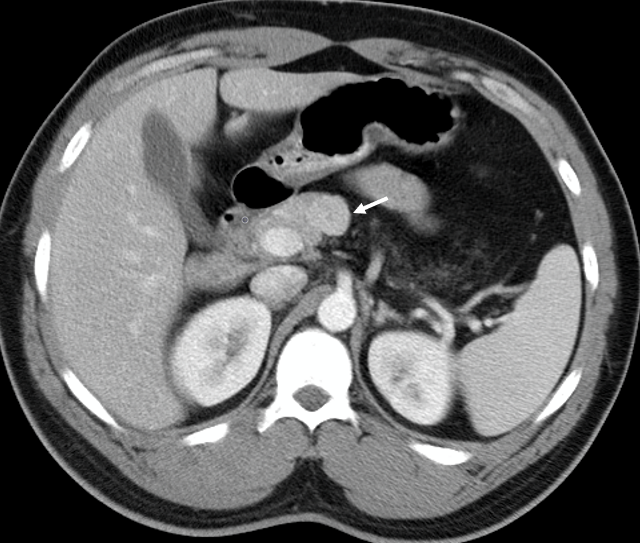

Supplement: Supplementary file 10 — Figure S10: Distal fatty pancreas due to a proximal neuroendocrine tumor in the pancreatic body (white arrow). [file UEG2-14-e70185-s013.tiff]

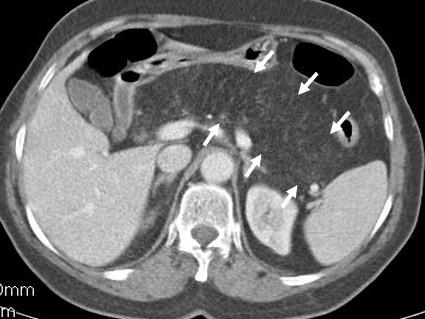

Supplement: Supplementary file 11 — Figure S11: Lipomatous pseudohypertrophy of the pancreas with enlargement of the pancreatic tissue with adipose tissue (white arrows). [file UEG2-14-e70185-s021.tiff]

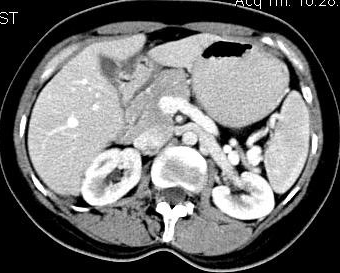

Supplement: Supplementary file 12 — Figure S12: Distal pancreatic agenesis and a dependent stomach. The splenic vein is shown touching the stomach. [file UEG2-14-e70185-s002.tiff]

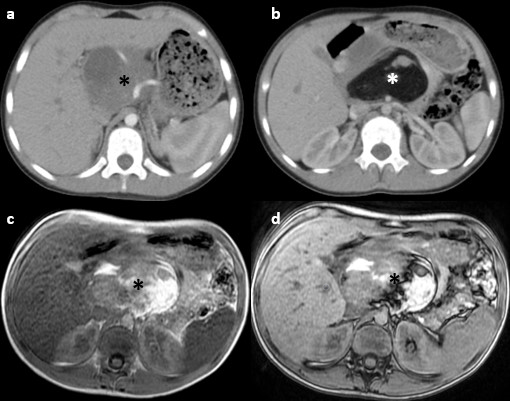

Supplement: Supplementary file 13 — Figure S13: Patient with a pancreatic fat‐containing mass (*) on computed tomography (a, b) with a solid component diagnosed with pancreatoblastoma. In in‐phase (c) and opposed‐phase (d) images, there is no signal drop due to the presence of microscopic fat. [file UEG2-14-e70185-s018.tiff]

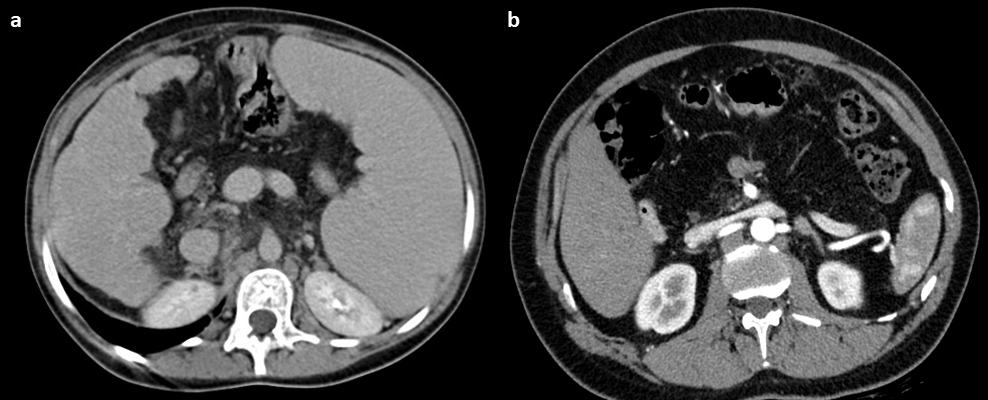

Supplement: Supplementary file 14 — Figure S14: Two patients with cystic fibrosis (a) and Schwachman–Diamond Syndrome (b) with diffuse fatty pancreas imaged using computed tomography. [file UEG2-14-e70185-s012.tiff]

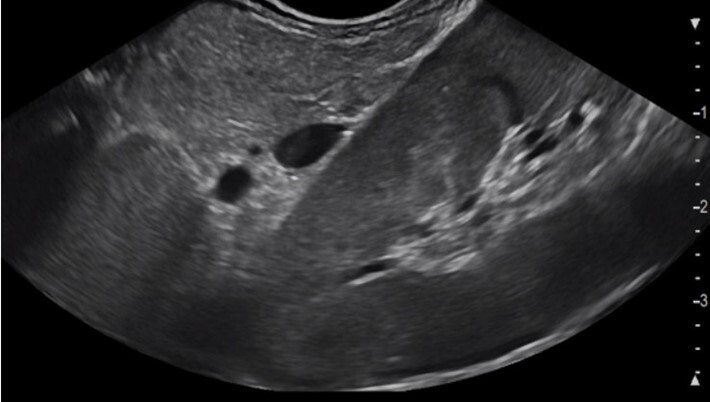

Supplement: Supplementary file 15 — Figure S15a: Shown are (a) endoscopic ultrasound (EUS)‐normal pancreas, (e) EUS‐mild‐to‐moderate fatty pancreas (body and tail). [file UEG2-14-e70185-s007.jpg]

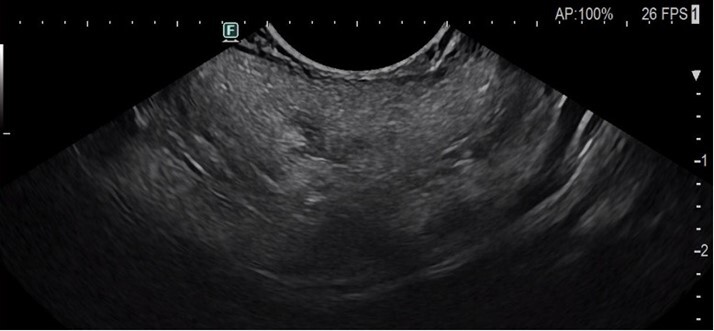

Supplement: Supplementary file 16 — Figure S15b: (b) EUS‐mild‐to‐moderate fatty pancreas (head). [file UEG2-14-e70185-s010.jpg]

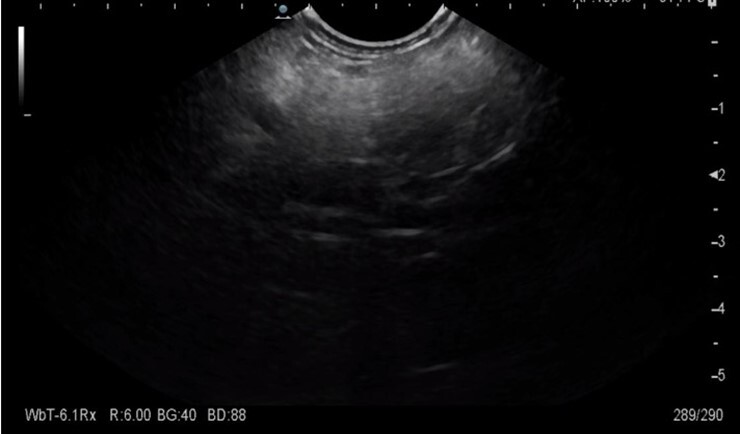

Supplement: Supplementary file 17 — Figure S15c: (c) EUS‐severe fatty pancreas (head). [file UEG2-14-e70185-s017.jpg]

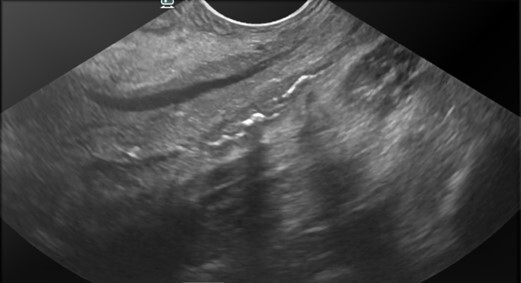

Supplement: Supplementary file 18 — Figure S15d: (d) EUS‐normal pancreas. [file UEG2-14-e70185-s006.jpg]

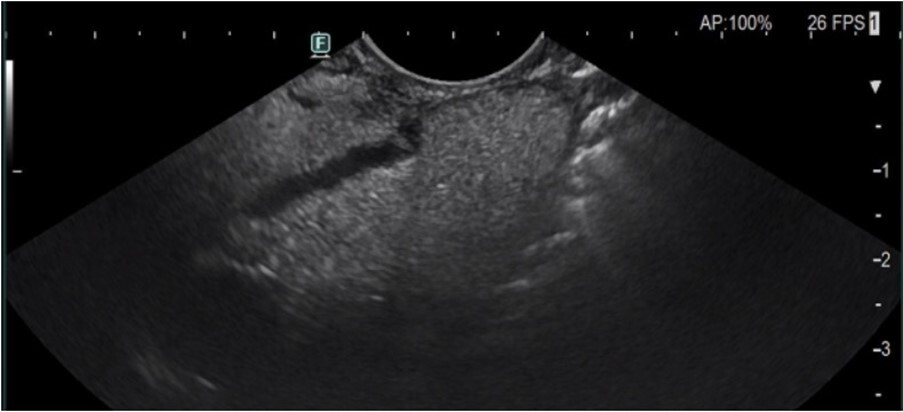

Supplement: Supplementary file 19 — Figure S15e: (e) EUS‐mild‐to‐moderate fatty pancreas (body and tail). [file UEG2-14-e70185-s014.jpg]

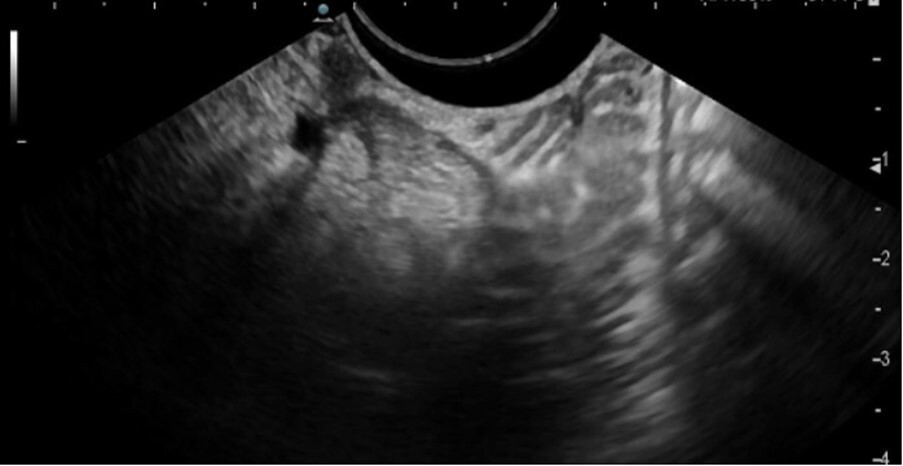

Supplement: Supplementary file 20 — Figure S15f: (f) EUS‐severe fatty pancreas (body and tail). [file UEG2-14-e70185-s009.jpg]
