# Supplementary material for: International Multidisciplinary Consensus Report on Definitions, Diagnostic Criteria, and Management of Fatty Pancreas: A Joint Statement Endorsed by EPC, APA, EASD, EASL, ESGAR, ESGE, ESP, ESPCG, ESPEN, ESPGHAN, IAP, JPS, KPBA, LAPSG, and UEG
Source: United European Gastroenterol J. 2026 Feb 14;14(1):e70185. doi: 10.1002/ueg2.70185 (PMC12906299; doi:10.1002/ueg2.70185)
Supplement: Supplementary file 21 — Table S1: Participating societies. [file UEG2-14-e70185-s001.docx]

**Supplement Table 1.** Participating societies and patient associations.

| European Pancreatic Club (EPC) |
| --- |
| American Pancreatic Association (APA) |
| European Association for the Study of Diabetes (EASD) |
| European Association for the Study of the Liver (EASL) |
| European Society of Gastrointestinal and Abdominal Radiology (ESGAR) |
| European Society of Gastrointestinal Endoscopy (ESGE) |
| European Society of Pathology (ESP) |
| European Society for Primary Care Gastroenterology (ESPCG) |
| European Society for Clinical Nutrition and Metabolism (ESPEN) |
| European Society for Pediatric Gastroenterology, Hepatology, and Nutrition (ESPGHAN) |
| International Association of Pancreatology (IAP) |
| Japan Pancreas Society (JPS) |
| Korean Pancreatobiliary Association (KPBA) |
| Latin American Pancreas Study Group (LAPSG) |
| United European Gastroenterology (UEG) |
| Arbeitskreis der Pankreatektomierten e.V. (Germany) |
| Digestive Cancers Europe |
| PALEMA (Sweden) |
| Pancreatic Cancer Europe |
