# Supplementary material for: International Multidisciplinary Consensus Report on Definitions, Diagnostic Criteria, and Management of Fatty Pancreas: A Joint Statement Endorsed by EPC, APA, EASD, EASL, ESGAR, ESGE, ESP, ESPCG, ESPEN, ESPGHAN, IAP, JPS, KPBA, LAPSG, and UEG
Source: United European Gastroenterol J. 2026 Feb 14;14(1):e70185. doi: 10.1002/ueg2.70185 (PMC12906299; doi:10.1002/ueg2.70185)
Supplement: Supplementary file 22 — Table S2: Overview of the working groups. [file UEG2-14-e70185-s011.docx]

**Supplement Table 2.** Overview of the working groups.

| 1. The name of the condition |
| --- |
| 2. Etiology of fatty pancreas |
| 3. Roles of alcohol and smoking in fatty pancreas |
| 4. Epidemiology of fatty pancreas |
| 5. Pathology of fatty pancreas |
| 6. Radiology of fatty pancreas |
| 7. Endoscopic ultrasound and fatty pancreas |
| 8. Acute pancreatitis and fatty pancreas |
| 9. Chronic pancreatitis and pancreatic exocrine insufficiency in fatty pancreas |
| 10. Intraductal papillary mucinous neoplasms and fatty pancreas |
| 11. Pancreatic cancer and fatty pancreas |
| 12. Pancreas surgery complications and fatty pancreas |
| 13. Children and the fatty pancreas |
| 14. Metabolic dysfunction-associated steatotic liver disease and fatty pancreas |
| 15. Beta-cell function and glucose homeostasis in fatty pancreas |
| 16. Treatment and monitoring of patients with fatty pancreas |
